# Stereochemical Assignment of the Protein–Protein Interaction Inhibitor JBIR-22 by Total Synthesis**

**DOI:** 10.1002/anie.201411141

**Published:** 2015-02-04

**Authors:** Alan R Healy, Miho Izumikawa, Alexandra M Z Slawin, Kazuo Shin-ya, Nicholas J Westwood

**Affiliations:** School of Chemistry and Biomedical Sciences Research Complex, University of St Andrews and EaStCHEMNorth Haugh, St Andrews, Fife (UK); Japan Biological Informatics Consortium (JBIC) 2-4-7 Aomi, Koto-kuTokyo 135-0064 (Japan); National Institute of Advanced Industrial Science and Technology (AIST)2-4-7 Aomi, Koto-ku, Tokyo 135-0064 (Japan)

**Keywords:** natural products, stereochemistry, tetramic acids, total synthesis, unnatural amino acids

## Abstract

Recent reports have highlighted the biological activity associated with a subfamily of the tetramic acid class of natural products. Despite the fact that members of this subfamily act as protein–protein interaction inhibitors that are of relevance to proteasome assembly, no synthetic work has been reported. This may be due to the fact that this subfamily contains an unnatural 4,4-disubstitued glutamic acid, the synthesis of which provides a key challenge. A highly stereoselective route to a masked form of this unnatural amino acid now enabled the synthesis of two of the possible diastereomers of JBIR-22 and allowed the assignment of its relative and absolute stereochemistry.

Natural products that contain the tetramic acid motif have been studied extensively, and their complexity and biological profiles have led to several total syntheses.[1] For example, equisetin, a close structural analogue of the compounds studied here, has been prepared.[1a–c] However, the synthesis of members of a subfamily that contain an unnatural 4,4-disubstituted glutamic acid unit (**1**–**4**, Figure 1) is an unmet challenge.[2] The biological activity displayed by members of this subfamily justifies the development of a concise and general approach for their synthesis.

**Figure 1:**
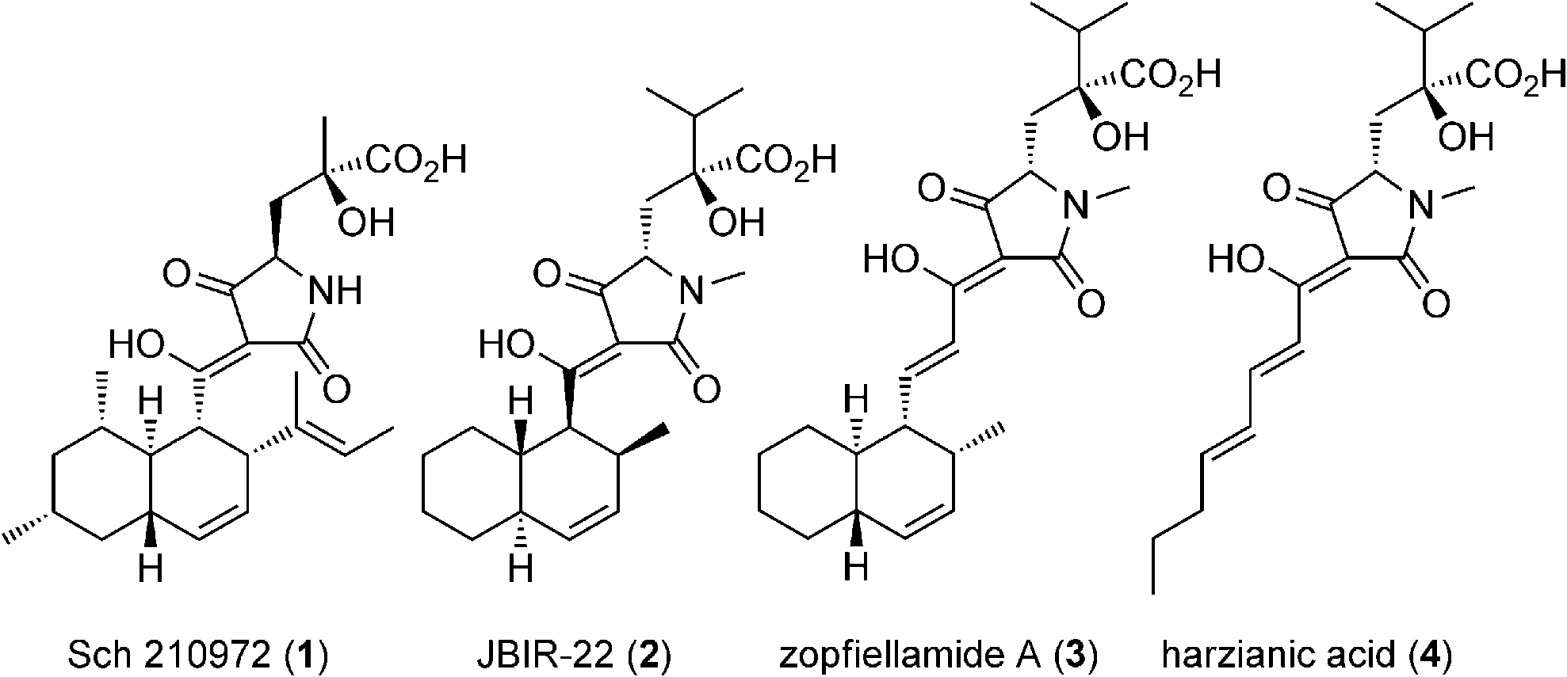
A subfamily of tetramic acid natural products containing an unnatural 4,4-disubstituted glutamic acid unit. Syntheses of 1–4 have not been reported thus far. The shown relative and absolute stereochemistry of 2 was assigned by our study.

Examples of the important activity shown by this subfamily include the inhibition of the CCR5 receptor by Sch210972 (**1**).[2a,b] A number of CCR5 receptor antagonists are in clinical trials or in use as antiretroviral drugs.[3], [4] In addition, JBIR-22 (**2**) is the first example of a tetramic acid that acts as a protein–protein interaction (PPI) inhibitor.[2c,d] Compound **2** inhibits the homodimerization of the proteasome assembly chaperone 3 (PAC3), an important protein involved in the formation of the proteasomal machinery. The clinical success of bortexomib,[5] a proteasome inhibitor, supports the study of compounds that target the proteasome or its formation. The fact that the stereochemical assignment of **2**[2c] was incomplete when our work began further highlights the need for synthetic studies on this subfamily of tetramic acids.

Although chemical[6] and enzymatic[7] syntheses of 4-hydroxy-4-methylglutamic acid have been developed, a synthesis of 4-hydroxy-4-*iso*-propylglutamic acid has not yet been reported, which could be a factor in the lack of synthetic work done on this subfamily. Here we report a short, stereoselective synthesis of a 4,4-disubstituted glutamic acid derivative and the application of this methodology to the first total synthesis of **2**. Our studies enabled the assignment of the relative and absolute stereochemistry of **2**.

Our initial synthetic plan was based on the synthesis of 1,3-amino alcohols (e.g. **5**). This methodology involved the diastereoselective addition of a metalloenamine **6** to an aldehyde followed by diastereoselective imine reduction (Scheme 1).[8] We proposed that the reaction of **7** with ethyl dimethylpyruvate could establish the required stereogenic center of the tertiary alcohol. Subsequent diastereoselective reduction of the resulting β-hydroxy-*N*-sulfinyl ketimine **8** could give **9**, a precursor of a protected form of the unnatural amino acid **10** (Scheme 1). If accessible, **10** could potentially be used in the synthesis of **2** in an analogous manner to that previously demonstrated for other tetramic acids containing natural amino acids, such as equisetin.[1a–c, 9] It also seemed plausible that the tertiary alcohol in **8** or **9** may cyclize to generate a lactone (e.g. **11** from **9**). If this occurred, *N*-methylation of **11** and removal of the *N*-sulfinyl group could give the masked 4,4-disubstituted glutamic acid derivative **12**. Conversion of **12** to members of this subfamily was considered achievable.

**Scheme 1:**
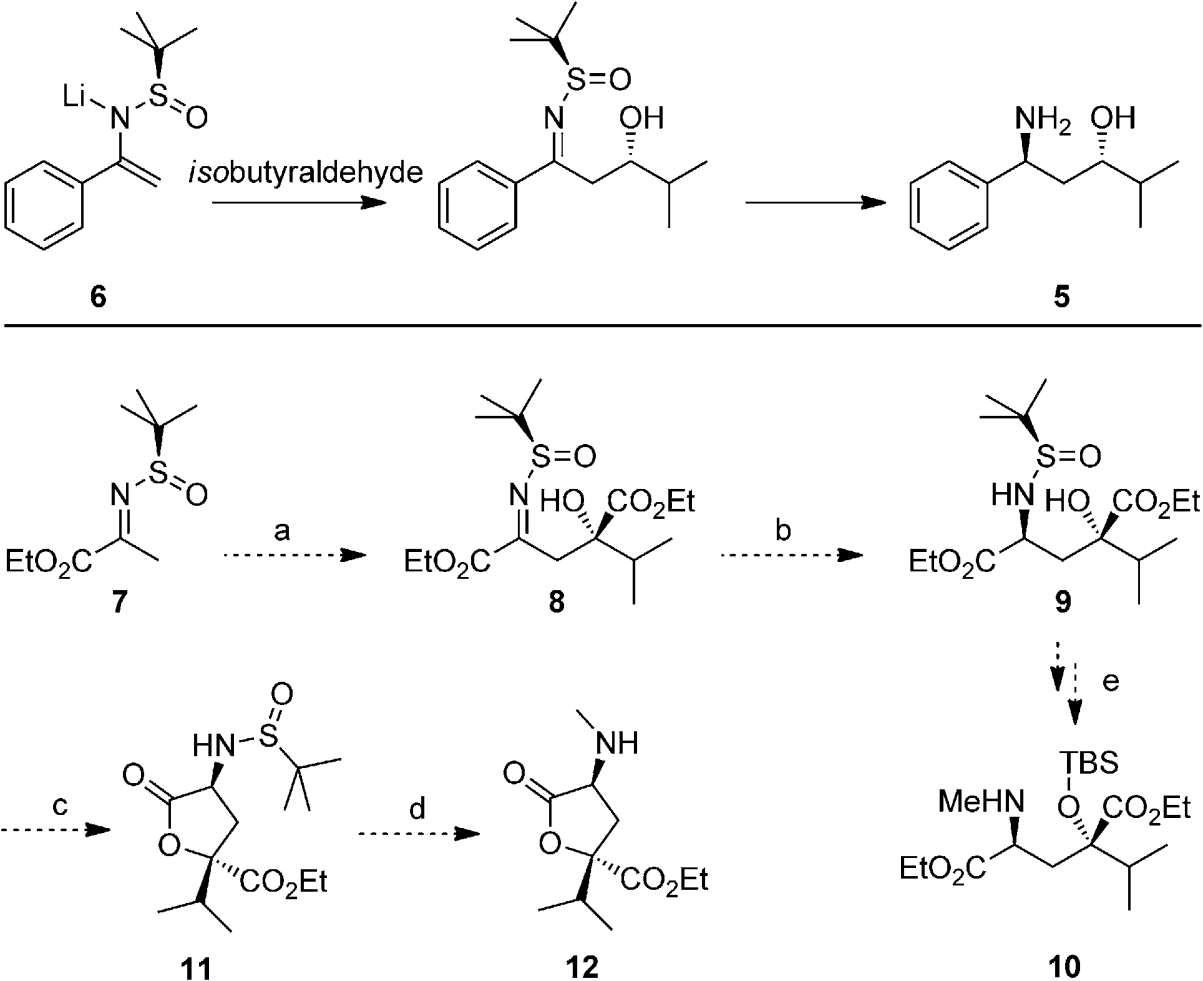
Ellman’s stereoselective synthesis of 1,3-amino alcohols.[8] A possible synthesis of the required unnatural amino acid or a cyclized version. Reagents and conditions: a) diastereoselective aldol reaction; b) diastereoselective reduction; c) lactonization; d) *N*-methylation and cleavage of the *N*-sulfinyl group; e) *N*-methylation and protecting group manipulation.

The synthesis of **7** was achieved by condensation of (*R_S_*)-*tert*-butanesulfinamide with ethyl pyruvate (**8**). Using the reported conditions,[10] **7** was obtained in only 30 % yield with the major product being lactone **13** (**13**:**7**=5:3, Scheme 2). The formation of **13** likely occurred in situ through an Ti(OEt)_4_-catalyzed aldol reaction of **7** with ethyl pyruvate (**8**) followed by lactonization (Scheme S1). Although **13** was not required for the preparation of **2**, it could be used in a future synthesis of **1**. Optimization of the synthesis of **7** resulted in its isolation in 60 % yield (Table S1). Reaction of **7** with ethyl dimethylpyruvate gave the related lactone **14** (Scheme 2) with excellent diastereoselectivity and yield. As expected, **14** was confirmed as the (*Rs*,2*S*) diastereomer by X-ray analysis (Scheme 2).[8], [11] *N*-methylation of **14** proceeded in high yield to provide **15**. While an initial screening of reducing agents gave only recovered lactone **15**, the use of NaBH_3_CN with HCl (4 n in dioxane) resulted in the diastereoselective (d.r.>98 %) reduction of **15** with cleavage of the *N*-sulfinyl group to give **12** (Scheme 2). The stereochemistry of **12** was assigned by NOE analysis (Scheme 2 and Figure S1). Further analysis suggested that this reaction proceeded by acid deprotection of the *N*-sulfinyl group followed by the reduction with NaBH_3_CN (Scheme S2). The observed diastereoselectivity was rationalized based on the preferred approach of the reducing agent from the same side as the ester. This efficient route provided the masked 4-hydroxy-4-*iso*propyl glutamic acid **12** in just four steps from **8**.

**Scheme 2:**
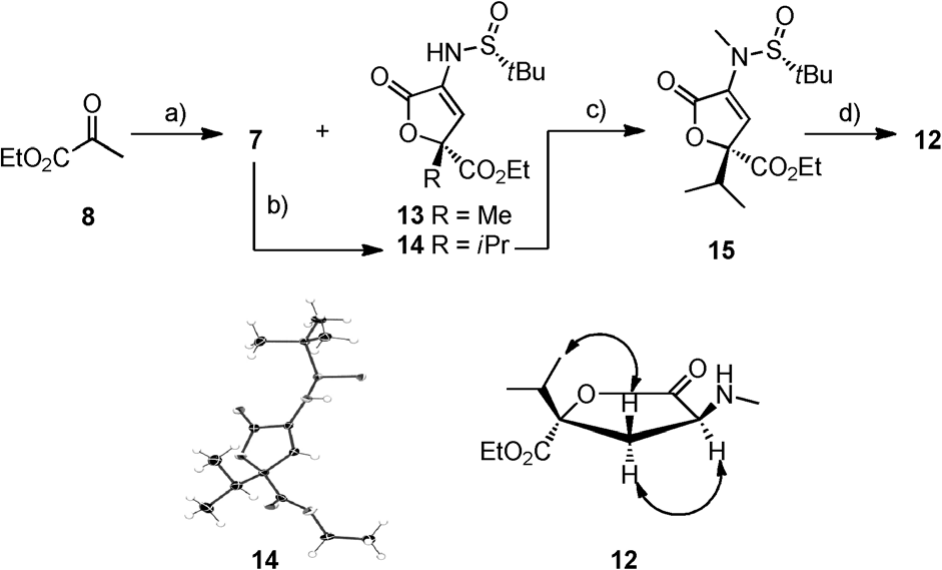
Condensation of 8 and (*R_S_*)-*tert*-butanesulfinamide gave lactone 13 and 7 in a 5:3 ratio. Reagents and conditions: a) (*R_S_*)-*tert*-butanesulfinamide, Ti(OEt)_4_, Table S1 for optimization; b) (i) LDA, THF, 0 °C. (ii) Ethyl dimethylpyruvate, ZnBr_2_, −78 °C, 88 %, d.r.>98 %; c) LiHMDS, iodomethane, DMF, −15 °C→RT, 95 %; d) (i) HCl (4 n in dioxane), THF, 0 °C, 10 min. (ii) NaBH_3_CN, MeOH, 1.5 h, 0 °C, 85 %, d.r.>98 %. X-ray analysis of 14 confirmed the expected (*Rs*,2*S*) stereochemistry. The stereochemistry of 12 was determined using NOE analysis (Figure S1).

With **12** in hand, a synthesis of **2** was attempted because of its unique activity as a PPI inhibitor and the uncertainty associated with its stereochemical assignment. Izumikawa et al. had shown that **2** could be assigned as one of the four stereoisomers shown in Table 1 (diastereomers **2 a** and **2 b** and their enantiomers **2 c** and **2 d**).[2c] Given the relatively large distance between the decalin moiety and the unnatural amino acid stereogenic center in **2**, it is difficult to assign the relative configuration of these two units. A convergent route to access optically enriched samples of diastereomers **2 a** and **2 b** was therefore investigated (Scheme 3).

**Scheme 3:**
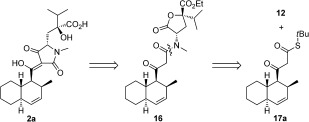
Retrosynthetic analysis of JBIR-22 diastereomer 2 a.

**Table 1:** Stereochemical assignment of four of the possible stereoisomers of 2 (as reported in reference [2c]).^[a]^ The absolute configuration of stereoisomer 2 a is depicted.

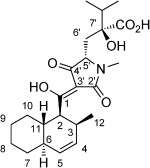	Compound	Glutamic acid side chain	Decalin ring
	**2 a**	5′*S*,7′*S*	2*S*,3*S*,6*R*,11*S*
	**2 b**	5′*S*,7′*S*	2*R*,3*R*,6*S*,11*R*
	**2 c**	5′*R*,7′*R*	2*R*,3*R*,6*S*,11*R*
	**2 d**	5′*R*,7′*R*	2*S*,3*S*,6*R*,11*S*

The tetramic acid core in **2 a** would be formed at a late stage, inspired by the conversion of 3-oxo-homoserine lactones to simple tetramic acids through a Claisen-like intramolecular reaction (Scheme S3).[12] A Lacey–Dieckmann condensation of fragment **16** would form the tetramic acid core and provide the unnatural 4,4-disubstituted glutamic acid side chain in one step. Fragment **16** could be accessible through the coupling of **12** and the β-ketothioester **17 a**. A late-stage convergent step such as this could ultimately facilitate the coupling of alternate β-ketothioesters to enable access to the other members of this subfamily (Figure 1) or novel analogues. We envisaged that the decalin β-ketothioester could be assembled through an asymmetric Diels–Alder cycloaddition followed by manipulation to introduce the thioester functionality (Schemes 4 and 5).

**Scheme 4:**
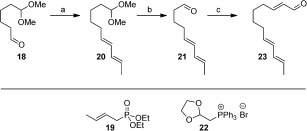
Synthesis of 23. Reagents and conditions: a) KHMDS, diethyl 2-butenylphosphonate (19), DME, −78 °C→RT, 69 %, *E*:*Z*=8:1; b) Aq. HCl, THF, RT, 12 h, 94 %; c) (i) (1,3-dioxolan-2-ylmethyl)triphenylphosphonium bromide (22), *t*BuOK, THF, 0 °C, 3.5 h. (ii) 10 % aq. oxalic acid, RT, 1 h, 89 %.

Assembly of **17 a**/**b** began with an Schreiber ozonolysis[13] of cyclohexene to give acetal **18**. Horner–Wadsworth–Emmons (HWE) olefination of **18** using phosphonate **19** provided **20** (8:1 mixture of inseparable *E*,*E*:*E*,*Z* isomers, Scheme 4). The acid-mediated deprotection of **20** gave dienal **21**, which was reacted with Wittig reagent **22**, followed by acetal hydrolysis to give the trienal **23** (85 % *E*,*E*,*E* geometry). Trienal **23** was then subjected to an organocatalytic intramolecular Diels–Alder (IMDA) reaction using MacMillan’s conditions (Scheme 5).[14] Both enantiomers of **24** were accessed with good enantioselectivities (see Scheme 5 and the Supporting Information for chiral GC analysis). The minor *E*,*Z*,*E* isomer present in the sample of **23** was inert in this IMDA reaction, thus enabling the purification to give either **24 a** or **24 b**, depending on which enantiomer of the organocatalyst was used (Scheme S4).[14] Elaboration of **24 a** and **24 b** to give β-ketothioesters **17 a** and **17 b**, respectively, was achieved through an aldol reaction using *S*-*tert*-butyl thioacetate to give **25 a** or **25 b**, respectively, as an inconsequential mixture of diastereomers, followed by oxidation with Dess–Martin periodinane[15] (Scheme 5).

**Scheme 5:**
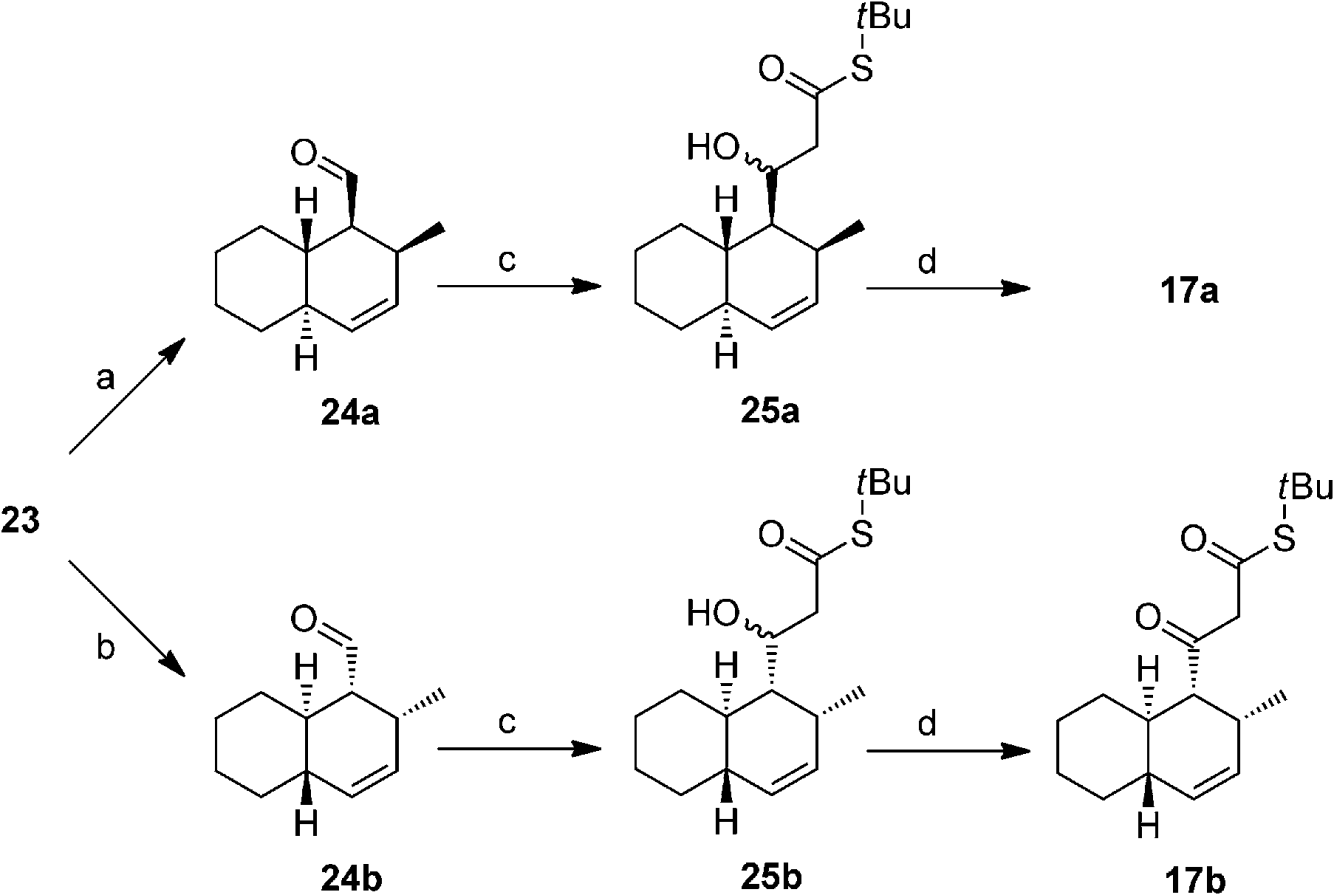
Synthesis of β-ketothioesters 17 a (Scheme 3) and 17 b. Reagents and conditions: a) 20 mol % (*S*,*S*)-imidazolidinone TfOH, MeCN (2 % H_2_O), −5 °C, 48 h, 65 %, 87 % *ee*, d.r. 4:1. b) 20 mol % (*R*,*R*)-imidazolidinone TfOH, MeCN (2 % H_2_O), −5 °C, 48 h, 68 %, 84 % *ee*, d.r. 4:1. c) (i) LDA, *S*-*tert*-butyl-thioacetate, THF, −78 °C, 30 mins. (ii) 24 a 24 b, THF, −78 °C, 2 h, 25 a (66 %); 25 b (69 %). d) Dess–Martin periodinane, DCM, RT, 2 h, 17 a (79 %); 17 b (82 %).

The final stages involved a silver trifluoroacetate mediated coupling of **12** with either enantiomer of fragment **17** to give **26 a** and **26 b**, following the protocol developed by the Ley group for the synthesis of equisetin (Scheme 6).[1b, 16] Finally, cyclization onto the lactone in **26 a** and **26 b** and microwave-assisted ester hydrolysis gave separate samples of the optically enriched diastereomers **2 a** and **2 b**, which were purified by reverse-phase chromatography. No evidence of epimerization at the C5’ position was observed.[17]

**Scheme 6:**
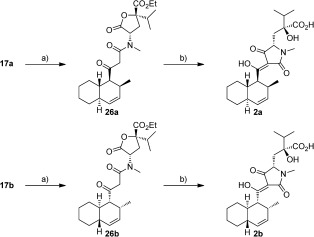
Synthesis of JBIR-22 diastereomers 2 a and 2 b. Reagents and conditions: a) 12, AgCF_3_CO_2_, Et_3_N, THF, 0 °C→RT, 2 h, 25 a—89 %; 25 b—84 %. b) (i) *t*BuOK, THF, 0 °C→RT, 2 h. (ii) Aq. NaOH, EtOH, 110 °C (MW), 20 mins, 2 a—71 %; 2 b—74 % over 2 steps.

The assignment of the relative stereochemistry of **2** was completed by comparison of the reported spectroscopic data[2c] for **2** with those obtained for our synthetic samples of **2 a** and **2 b**. This analysis revealed very similar ^1^H NMR signals, but clear differences in the ^13^C NMR spectra, with the signals reported for the isolated sample of **2** all being within ±0.1 ppm of those obtained for diastereomer **2 a**. In contrast, there were significant differences when the data was compared to that for diastereomer **2 b** (Figure 2 for selected examples and Table S2). Further evidence for the identical relative stereochemistry in **2** and diastereomer **2 a** came from doping experiments using UPLC-TOFMS (Figure 2). These studies showed that upon mixing of a sample of natural **2** (retention time=3.3 min) with **2 a**, an increase in the size of the peak at 3.3 min was observed, whereas doping of natural **2** with **2 b** led to the appearance of a different peak with a retention time of 3.6 min. Comparison of the specific rotation of **2 a** (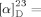
+75.0°, *c*=0.1, MeOH) with that obtained for natural **2** (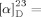
+62.0°, *c*=0.1, MeOH)[18] enabled the assignment of the absolute configuration of **2** as (2*S*, 3*S*, 6*R*, 11*S*, 5′*S*, 7′*S*).

**Figure 2:**
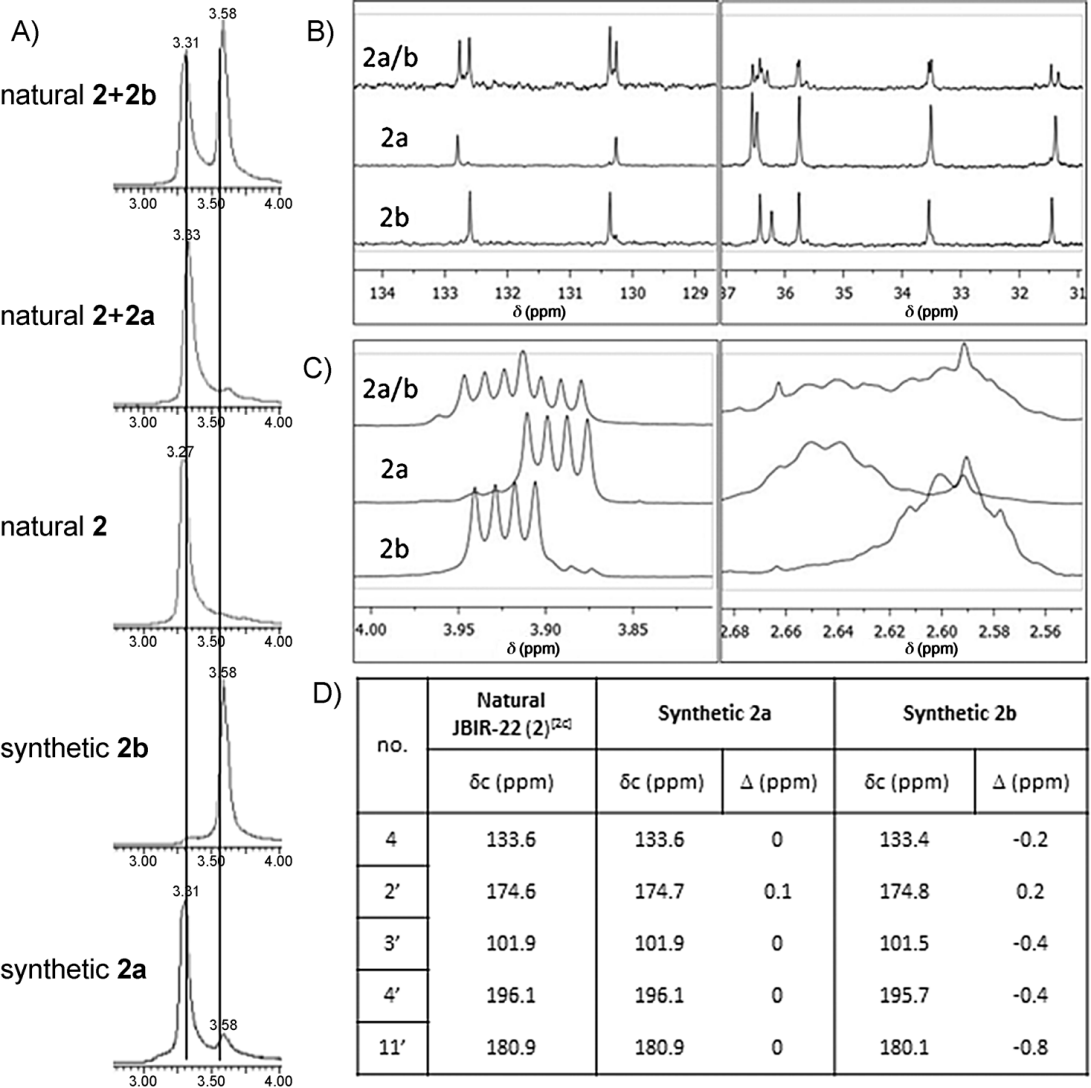
A) UPLC-TOFMS doping experiment. B) Selected ^13^C NMR signals of 2 a and 2 b with 2 a/b (a 1:1 mixture of 2 a and 2 b synthesized following an alternative route, Scheme S5). C) Selected ^1^H NMR signals of 2 a and 2 b with 2 a/b. D) Selected ^13^C NMR chemical shifts of isolated 2[2c] and 2 a and 2 b (see Supporting Information for full table). UPLC-TOFMS=ultra-performance liquid chromatography coupled to time-of-flight mass spectrometry.

In summary, a highly stereoselective synthesis of the masked 4,4-disubstituted glutamic acid **12** enabled the first total synthesis of highly enantioenriched samples of two of the possible diastereomers of JBIR-22 (**2**) by a concise, convergent strategy. The diastereomers **2 a** and **2 b** were synthesized in ten steps (longest linear route from cyclohexene) in 10.1 % and 11.3 % overall yield, respectively. The synthesis of two of the possible stereoisomers facilitated the assignment of both the relative and absolute configuration of the naturally occurring protein–protein interaction inhibitor **2**. The development of a short, stereoselective synthesis of **12** coupled with the convergent nature of this approach should facilitate the future synthesis and biological assessment of all members of this subfamily of natural products as well as novel analogues.
