# Supplementary material for: Stereochemical Assignment of the Protein–Protein Interaction Inhibitor JBIR-22 by Total Synthesis
Source: Angew Chem Int Ed Engl. 2015 Feb 4;54(13):4046–50. doi: 10.1002/anie.201411141 (PMC4441253; doi:10.1002/anie.201411141)
Supplement: Supplementary file 1 [file anie0054-4046-sd1.pdf]

Angewandte Chemie
Eine Zeitschrift der Gesellschaft Deutscher Chemiker

Supporting Information

Stereochemical Assignment of the Protein–Protein Interaction Inhibitor JBIR-22 by Total Synthesis**

*Alan R. Healy, Miho Izumikawa, Alexandra M. Z. Slawin, Kazuo Shin-ya, and Nicholas J. Westwood**

anie_201411141_sm_miscellaneous_information.pdf

Contents

1 Additional experimental information 3

1.1 Proposed formation of **13** 3

1.2 Optimization of the condensation reaction of ethyl pyruvate (**8**) and (R_S)-*tert*-butanesulfinamide 4

1.3 NOE analysis of **12** 5

1.4 Mechanistic investigation into the tandem deprotection-reduction of **15** to give **12**. 6

1.5 Intramolecular Claisen-like reaction of 3-oxo-AHLs to form tetramic acids 6

1.6 NOE analysis of **17** 7

2 Chiral GC analysis 8

3 Synthesis of a 1:1 mixture of JBIR-22 diastereomers **2a** and **2b** 11

4 NMR comparison 12

5 General information 13

6 Experimental procedures 14

7 NMR spectra of novel compounds 30

8 Bibliography 54

1 Additional experimental information

1.1 Proposed formation of 13

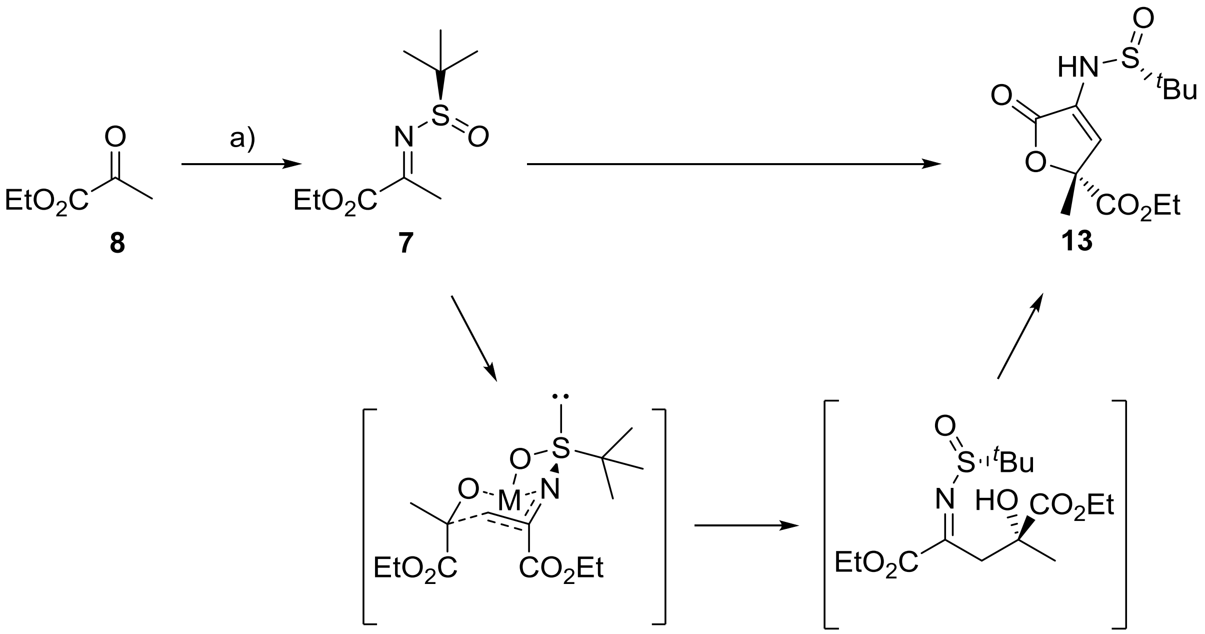

Scheme S1. Lactone **13** likely forms via an in situ aldol reaction of initially formed imine **7** and remaining ethyl pyruvate (**8**) followed by an intramolecular cyclisation to provide **13**. The relative and absolute configuration of **13** is proposed based on X-ray crystallographic analysis of the related compound **12**. The observed diastereoselectivity in the formation of **12** and **13** can be rationalized using a similar Zimmerman-Traxler transition state model to that proposed by Ellman for the condensation of *N*-sulfinyl imines to simple aldehydes.[1,2]

1.2 Optimization of the condensation reaction of ethyl pyruvate (8) and (R_S)-*tert*-butanesulfinamide

Table S1. A selection of the key results obtained from a screen of reaction conditions for the condensation of ethyl pyruvate (**8**) and (R_S)-*tert*-butanesulfinamide. *Reagents and conditions*: (**a**) (R_S)-*tert*-butanesulfinamide, THF. Ratio of **7**:**13** was determined by ^{1}H NMR analysis of the crude reaction mixture.

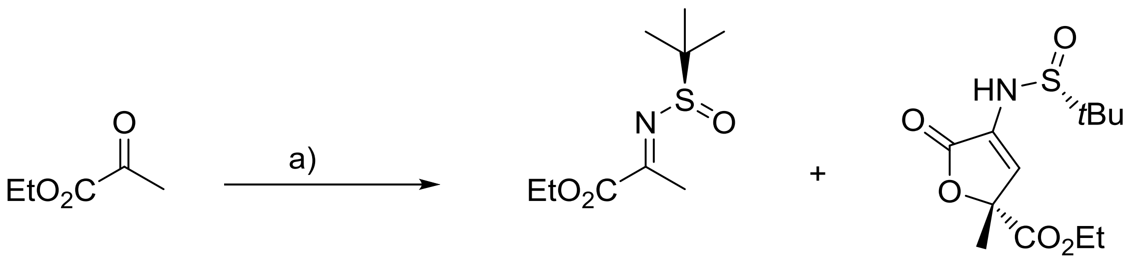

8 **7** **13**

**Entry**	**Lewis acid**	**Temperature (°C)**	**Time (hours)**	**7 (isolated yield)**	**7:13 (% by NMR)**
**1**	$Ti(OEt)_4$	65	6	36	3:5
**2**	$Ti(OEt)_4$	r.t.	6	23	8:1
**3**	$MgSO_4$	65	6	-	-
**4**	$ZnBr_2$	65	6	-	-
**5**	$Ti(O\textit{i}Pr)_4$	65	6	22	2:5
**6**	$TiCl_4$	0 → r.t.	3	-	-
**7**	$Ti(OEt)_4$	40	6	29	4:1
**8**[a]	$Ti(OEt)_4$	65	4	60	3:1

[a] (R_S)-*tert*-butanesulfinamide and $Ti(OEt)_4$ were heated to 65 °C in THF prior to addition of ethyl pyruvate (**8**) in one portion.

1.3 NOE analysis of 12

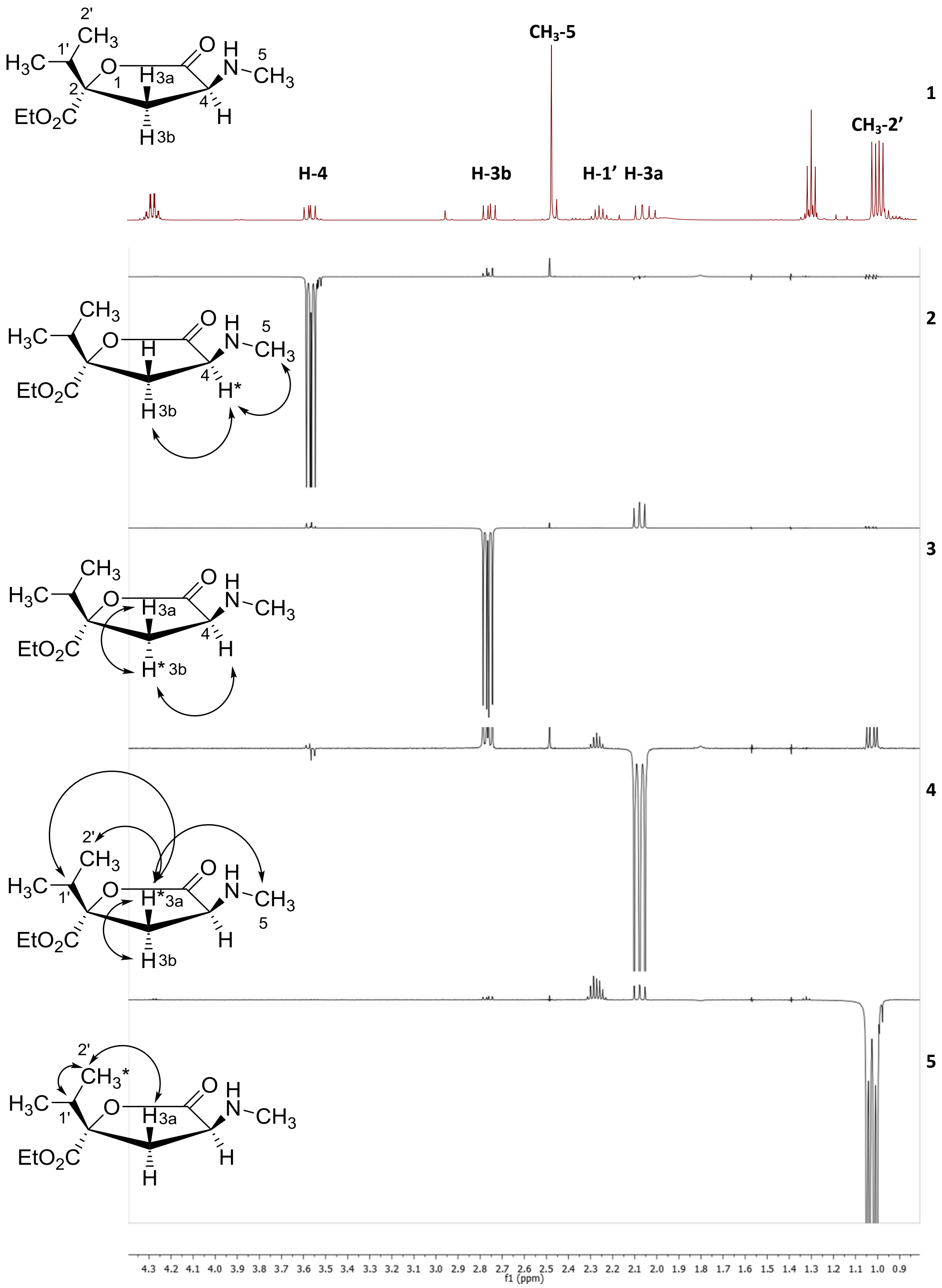

Figure S1. NOE analysis of **12**. (1) ^{1}H NMR spectra of **12**. * denotes irradiated proton(s). (2) An NOE was observed between H-4 and CH$_3$-5/H-3b. (3) An NOE was observed between H-3b and H-3a/H-4. COSY and HSQC experiments revealed that H-3a and H-3b are connected to the same carbon atom (see assignment of spectra for **12** below). (4) strong NOEs were observed between H-3a and H-3b/CH$_3$-5/CH$_3$-2'/H-1'. (5) strong NOEs were observed between CH$_3$-2' and H-1'/H-3a. The above data revealed that the 2° amine and *iso*propyl substituents were on the same face. As the C2 stereogenic centre had previously been assigned as (*S*) by small molecule X-ray crystallographic analysis of **14**, the absolute configuration of **12** was assigned as (2*S*, 4*S*).

1.4 Mechanistic investigation into the tandem deprotection-reduction of 15 to give 12.

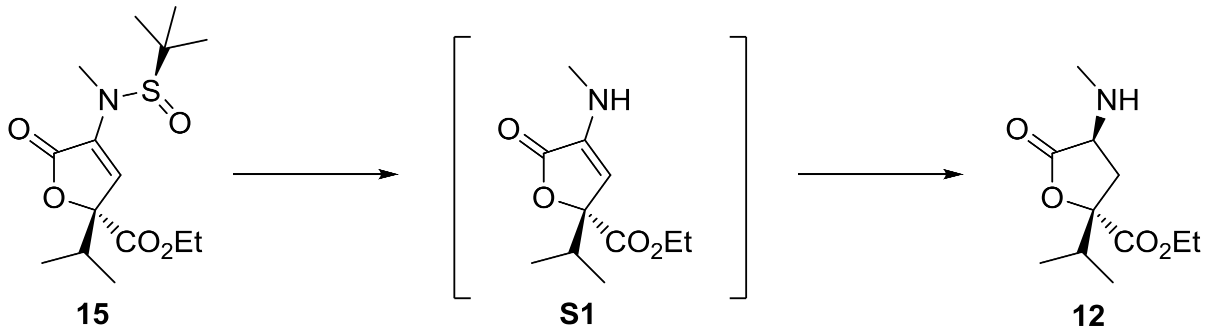

Scheme S2. Mechanistic studies were carried out to elucidate the reaction sequence in the tandem deprotection-reduction. The experimental procedure involved the addition of HCl (4N in dioxane) to **15** in THF at 0 °C followed by stirring for 10 minutes. Work-up of the reaction prior to the addition of the reducing agent and analysis of the crude reaction mixture by ^{1}H NMR (data not shown) revealed that the deprotected enamine **S1** was formed. Subsequent $NaBH_3CN$-mediated reduction of **S1** in the presence of HCl (4N in dioxane) provided the desired product **12** with similar diastereoselectivity but diminished yield when compared to the one-pot process. As the chiral auxiliary was cleaved prior to the addition of the reducing agent, the observed diastereoselectivity appears to be purely substrate controlled with the hydride attacking from the same side as the ester substituent. The high level of diastereocontrol would suggest that it may not be purely sterically induced and could be a result of coordination of the reducing agent by the ester, directing the attack of the hydride.

1.5 Intramolecular Claisen-like reaction of 3-oxo-AHLs to form tetramic acids

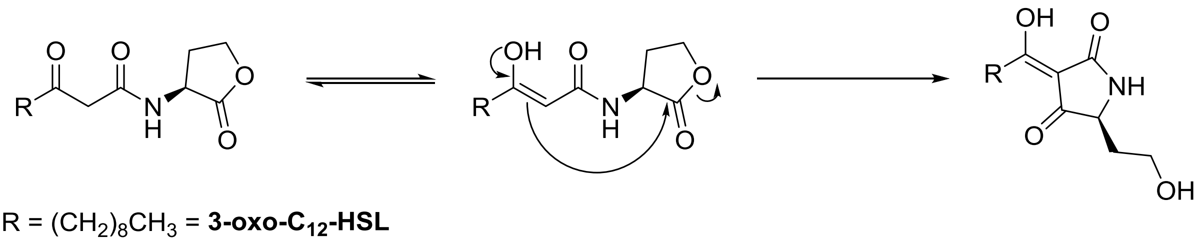

R = $(CH_2)_8CH_3$ = **3-oxo-C$_{12}$-HSL**
R = $(CH_2)_{10}CH_3$ = **3-oxo-C$_{14}$-HSL**

Scheme S3. Previously reported conversion of 3-oxo-AHLs to the corresponding tetramic acids via an intramolecular Claisen-like reaction.[3–5]

1.6 NOE analysis of 17

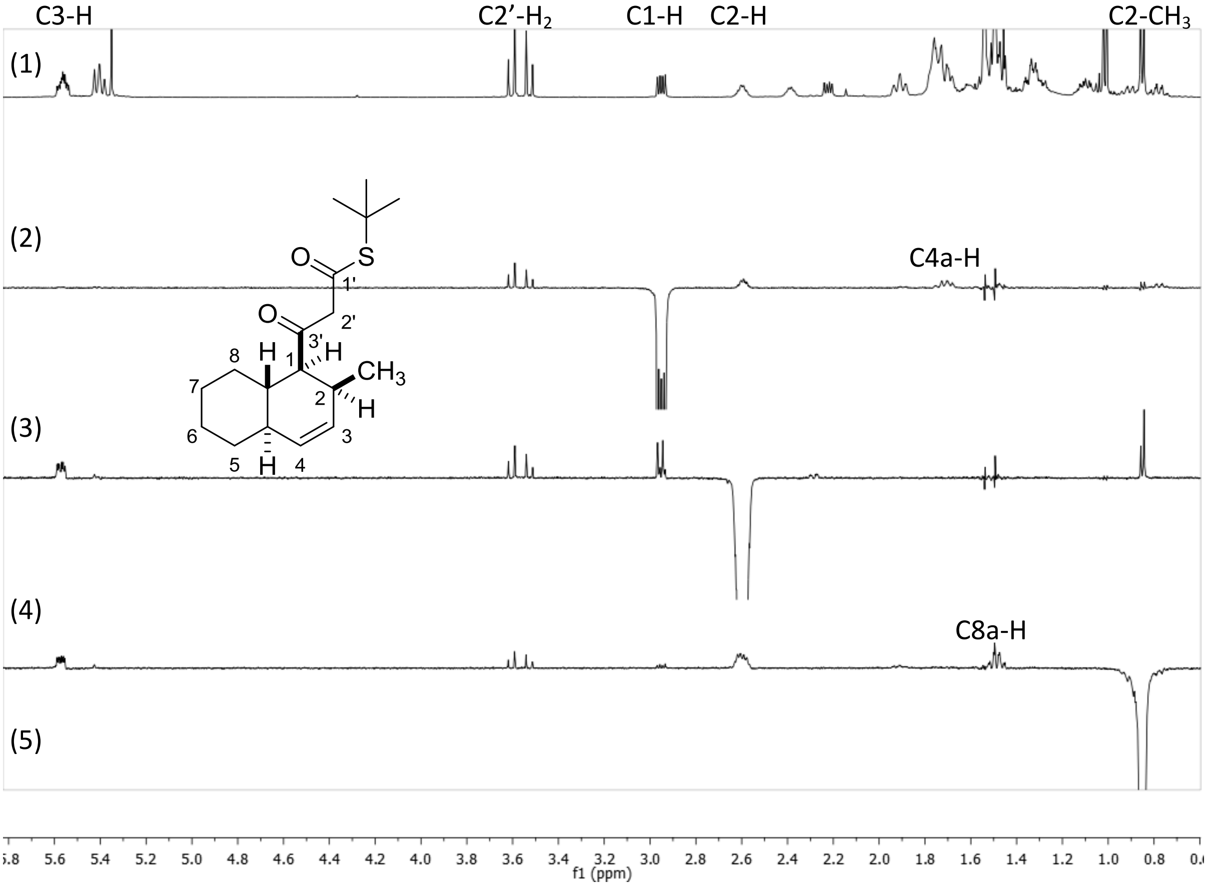

(1) In $CDCl_3$ at room temperature the **17** exists as a (3 : 1) *keto* : *enol* mixture. NOE analysis of the major *keto* tautomer is shown. (2) An NOE was observed between C1-H and C2’-H_2, C2-H and C4a-H, demonstrating C1-H, C2-H and C4a-H are cofacial. (3) An NOE was observed between C2-H and C1-H, C2’-H_2, C2-CH_3 and C3-H, confirming the presence of C1-H and C2-H on the same face of the decalin ring. (4) An NOE was observed between C2-CH_3 and C2-H, C2’-H_2, C8a-H and C3-H, demonstrating C2-CH_3 and C8a-H are cofacial. The NOE analysis confirms the expected relative stereochemistry of **17**.

2 Chiral GC analysis

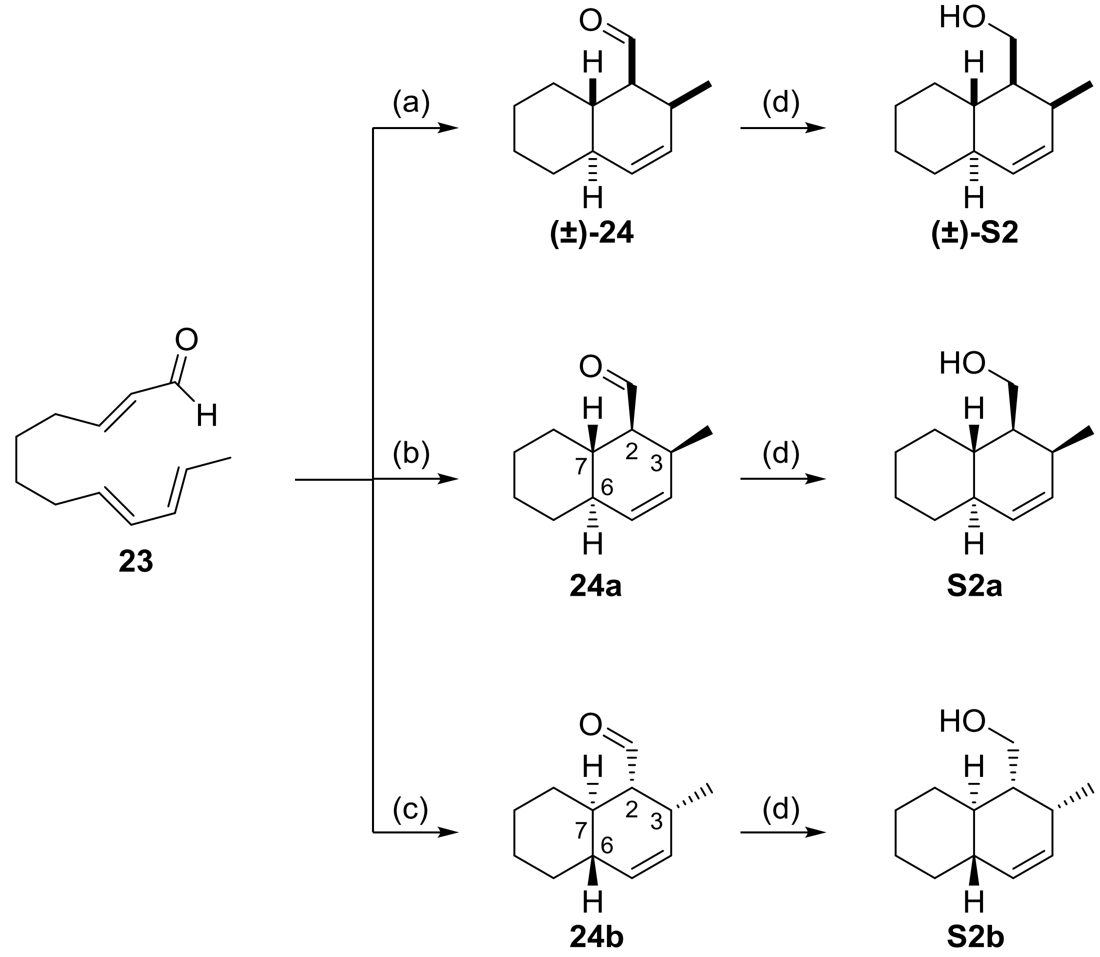

Scheme S4. (A) Synthesis of racemic decalin **(±)-24** and enantiomerically enriched decalins (2*S*,3*S*,6*R*,7*S*)-**24a** and (2*R*,3*R*,6*S*,7*R*)-**24b**. *Reagents and conditions*: (a) BF_3.Et_2O, DCM, -78 °C → 0 °C, 3 h, 74%. (b) 20 mol% (*S*,*S*)-imidazolidinone TfOH, MeCN (2% H_2O), -5 °C, 48 h, 65%, *dr* 4:1, 87% ee. (c) 20 mol% (*R*,*R*)-imidazolidinone TfOH, MeCN (2% H_2O), -5 °C, 48 h, 68%, *dr* 4:1, 84% ee. (d) $NaBH_4$, EtOH, 0 °C, 1 h, **(±)-S2** - 86%; **S2a** – 88%; **S2b** – 91%.

Trienal **23** was subjected to an organocatalytic intramolecular Diels-Alder (IMDA) reaction using MacMillan's conditions.[6] The moderate diastereoselectivity observed in the formation of **24a** and **24b** is in agreement with the findings of Christmann *et al.*[7] for the same transformation. This reduction in diastereoselectivity may be due to epimerisation of the C2 position which was also observed by MacMillan for similar substrates.[6] Throughout subsequent steps in the synthesis, the amounts of products resulting from the minor diastereomer reduced (on purification) although it was not possible to remove these minor products completely until the final step. The aldehydes **24a** and **24b** were converted to the corresponding alcohols **S2a** and **S2b** via $NaBH_4$ mediated reduction

for determination of their enantiomeric purity. A racemic standard was obtained by a $BF_3.OEt_2$ catalysed cycloaddition of **23** to provide (±)-**24** as a single diastereomer, which was subsequently reduced to the corresponding alcohol (±)-**S2**. Enantiomeric excesses were obtained by chiral GC analysis using an Agilent Cyclosil-B (isotherm, 140 °C, see below). The determined enantiomeric purity of **24a** (87% ee) and **24b** (84% ee) are in agreement with the reported values for this reaction (~80-90% ee).[6–8] The enantiomeric purity could be improved if required by recrystallization at the alcohol oxidation state and subsequent reoxidation.[9]

The minor diastereomers of **S2a** and **S2b** can be observed with retention times at approximately 21-22 minutes.

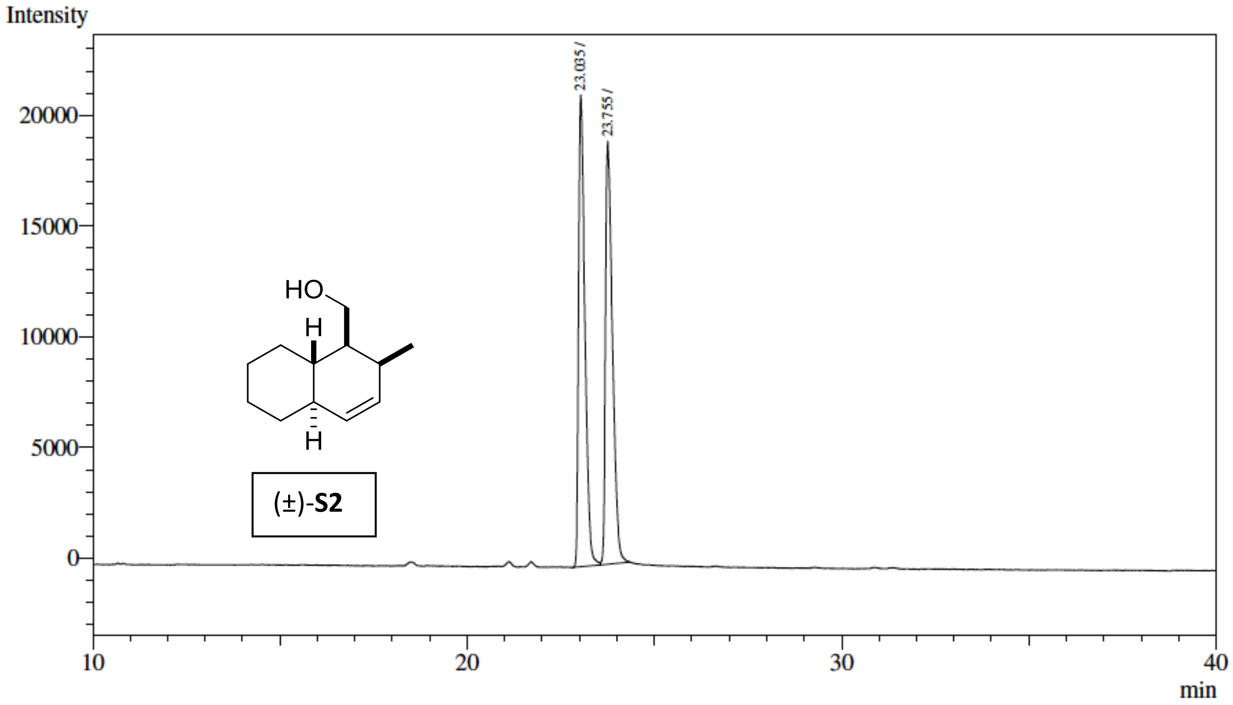

Peak#	Ret.Time	Area	Area%
1	23.035	247722	50.0871
2	23.755	246861	49.9129
Total		494583	100.0000

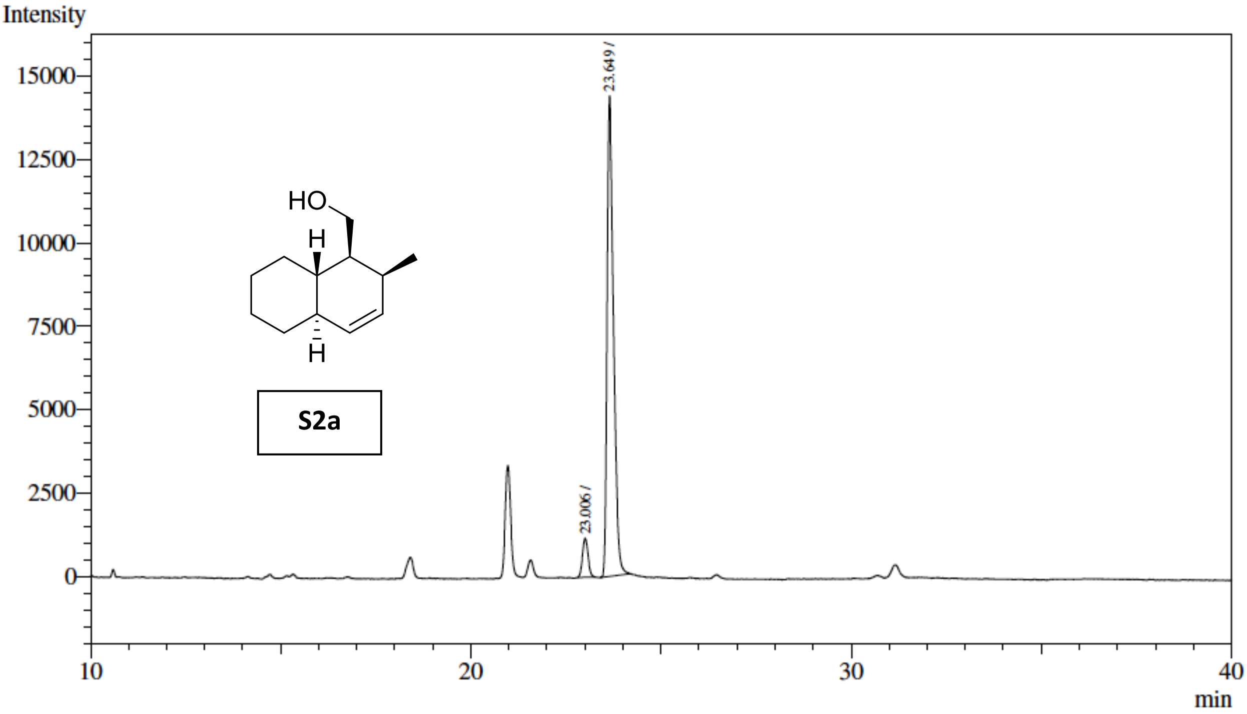

Peak#	Ret.Time	Area	Area%
1	23.006	11848	6.5768
2	23.649	168304	93.4232
**Total**		180152	100.0000

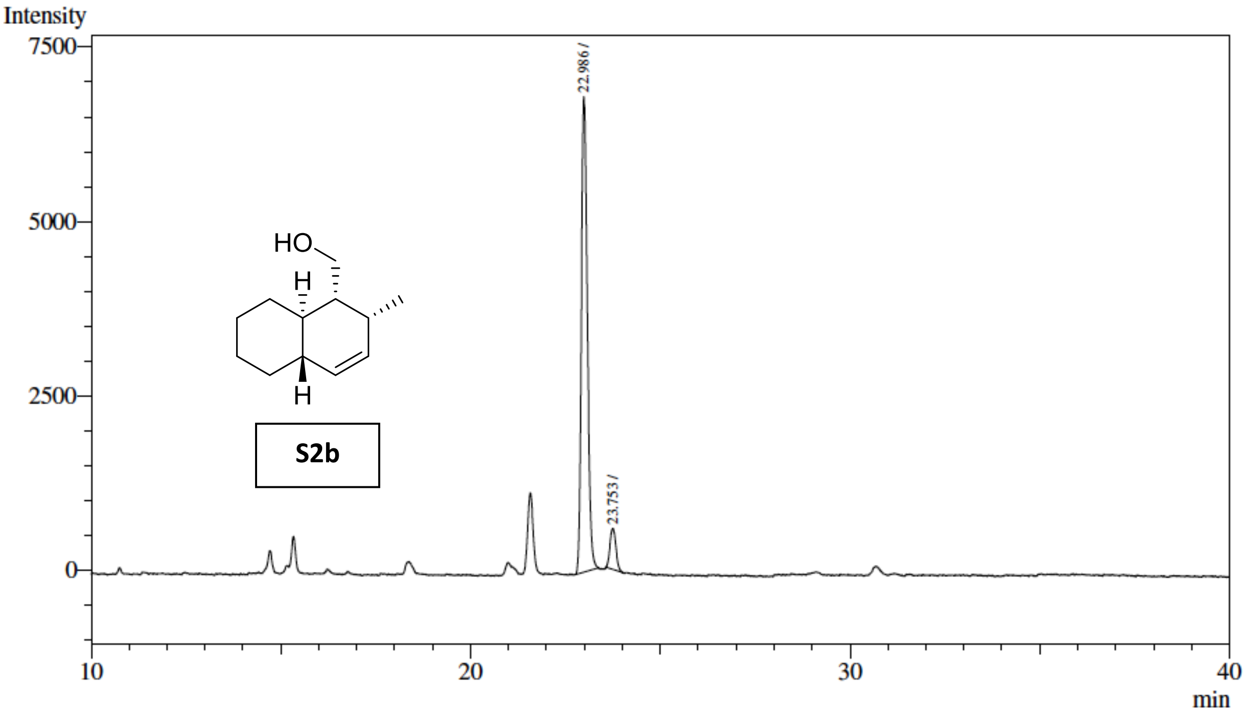

Peak#	Ret.Time	Area	Area%
1	22.986	74556	92.1743
2	23.753	6330	7.8257
**Total**		80886	100.0000

3 Synthesis of a 1:1 mixture of JBIR-22 diastereomers 2a and 2b

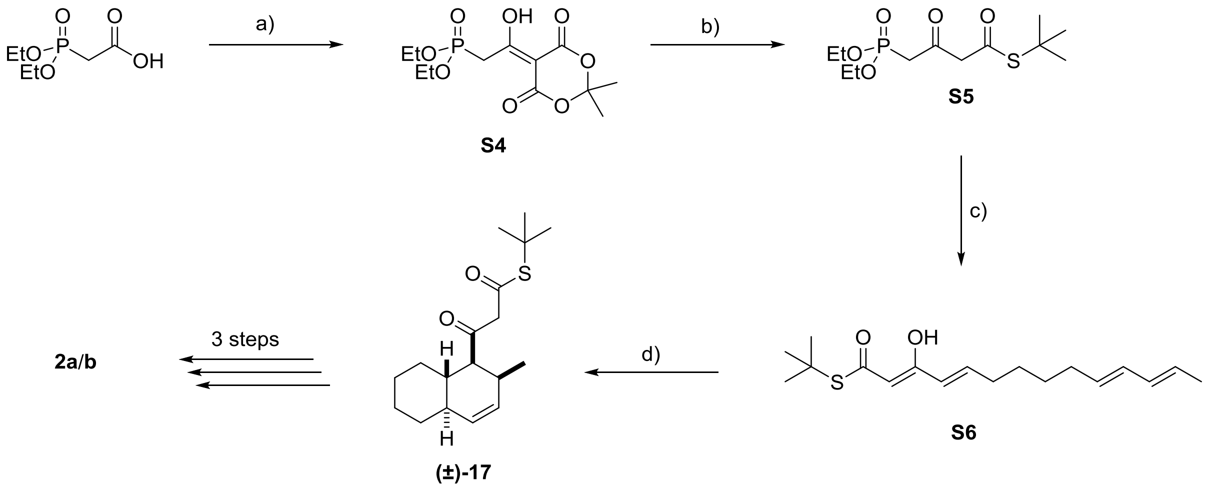

Scheme S5. Synthesis of **2a/b** (a 1:1 mixture of diastereomers **2a** and **2b**). a) (i) $SOCl_2$, rt., 3 h. (ii) Meldrum's acid (2,2-dimethyl-1,3-dioxane-4,6-dione), pyridine, DCM, 0 °C → rt., quantitative; b) 2-Methyl-2-propanethiol, MeCN, reflux, 3 h, 85%; c) LiHMDS, **21**, THF, -78 °C → 0 °C, 3 h, 72%; d) $BF_3.Et_2O$, DCM, -78 °C → 0 °C, 73%.

4 NMR comparison

Table S2. Comparison of the reported physico-chemical and spectroscopic properties of isolated JBIR-22 (**2**) and synthesised JBIR-22 diastereomers **2a** and **2b**. ^{13}C NMR signals obtained for **2a** and **2b** which are different by ±0.1 ppm to that reported for the natural sample of JBIR-22 (**2**) are highlighted.

JBIR-22 (2)[10] (reported for isolated sample)			2a (synthesised)		2b (synthesised)	
$[\alpha]_D^{23}$	+62.0 (*c* 0.1, MeOH[a])		+75.0 (*c* 0.1, MeOH)		-42.0 (*c* 0.1, MeOH)	
**HRMS**	420.2385 [M+H]$^+$		418.2226 [M-H]$^-$		418.2226 [M-H]$^-$	
**Position**	δ_C (ppm)	δ_H (ppm)	δ_C (ppm)	δ_H (ppm)	δ_C (ppm)	δ_H (ppm)
	(**Acetone-d$_6$**, 600 MHz)		(**Acetone-d$_6$**, 500 MHz)		(**Acetone-d$_6$**, 500 MHz)	
**1**	196.4		196.3		196.4	
**2**	51.0	3.90	51.0	3.90	51.0	3.92
**3**	32.2	2.64	32.2	2.64	32.3	2.60
**4**	133.6	5.53	133.6	5.54	133.4	5.52
**5**	131.1	5.30	131.1	5.30	131.2	5.30
**6**	43.4	1.68	43.4	1.68	43.3	1.69
**7**	34.3	1.69<br>1.03	34.4	1.70<br>1.03	34.4	1.71<br>1.03
**8**	27.6	1.65<br>1.28	27.6	1.65<br>1.28	27.7	1.69<br>1.28
**9**	27.7	1.65<br>1.28	27.7	1.65<br>1.28	27.7	1.69<br>1.28
**10**	31.0	1.97<br>0.71	31.1	1.95<br>0.71	31.0	2.00<br>0.71
**11**	37.4	1.46	37.4	1.46	37.3	1.45
**12**	18.3	0.76	18.4	0.77	18.3	0.75
**2'**	174.6		174.7		174.8	
**3'**	101.9		101.9		101.5	
**4'**	196.1		196.1		195.7	
**5'**	63.3	3.36	63.3	3.41	63.2	3.36
**6'**	37.3	2.30<br>1.60	37.3	2.31<br>1.62	37.1	2.31<br>1.64
**7'**	80		80.0		79.9	
**8'**	36.6	2.01	36.6	2.01	36.6	2.00
**9'**	18.7	0.91	18.8	0.91	18.6	0.91
**10'**	26.8	2.71	26.9	2.73	26.8	2.72
**11'**	180.9		180.9		180.1	
**12'**	16.8	0.89	16.9	0.90	16.8	0.91

a The reported specific rotation of natural JBIR-22 (**2**) in this manuscript ($[\alpha]_D^{23}$ = +62.0 ,c 0.1, MeOH) is a correction of the original specific rotation reported in the literature, see Ref [2c].

5 General information

All chemicals and solvents were purchased from Aldrich (UK), Alfa Aesar, or Acros Organics and used without further purification. All reactions involving moisture sensitive reagents were performed in oven or flame dried glassware under a positive pressure of nitrogen. Tetrahydrofuran (THF), dichloromethane (DCM) and hexanes were obtained dry from a solvent purification system (MBraun, SPS-800). Anhydrous *N*,*N*-dimethylformamide (DMF) was purchased from Aldrich. Thin layer chromatography (TLC) analysis was performed using glass plates coated with silica gel (with fluorescent indicator UV_{254}). Developed plates were air dried and analysed under a UV lamp (254/365 nm) or by $KMnO_4$ dip staining. Flash chromatography was performed using silica gel (40-63 µm, Fluorochem). Melting points were recorded in open capillaries using an Electrothermal 9100 melting point apparatus. Values are quoted to the nearest 1 °C and are uncorrected. Fourier Transform infra-red spectra (FT IR) were acquired on a Perkin Elmer paragon 1000 FT spectrophometer (KBr disc) or a Shimadzu IRAffinity-1 FT spectrophotometer with a Pike MIRacle™ (solid or thin film). Absorption maxima are reported in wavenumbers (cm^{-1}). Nuclear magnetic resonance (NMR) spectra were recorded at room temperature on Bruker Avance 500 (^{1}H, 499.9 MHz; ^{13}C, 125.7 MHz), Bruker Avance 400 (^{1}H, 400.1 MHz; ^{13}C, 100.6 MHz) and Bruker Avance 300 (^{1}H, 300.1 MHz; ^{13}C, 75.5 MHz) instruments. NMR spectra were recorded in deuterated solvents and internally referenced to the residual solvent peak, chloroform-*d* (δ_C 77.16, δ_H 7.26 ppm), acetone-d_6 (δ_C 29.8, δ_H 2.04 ppm), DMSO-d_6 (δ_C 39.52, δ_H 2.50 ppm) or methanol-d_4 (δ_C 49.0, δ_H 3.31 ppm). Chemical shifts are expressed as δ in units of ppm. ^{13}C NMR spectra were recorded using the PENDANT sequence mode. Data processing was carried out using the MestReNova 8.1.1 NMR program (Mestrelab Research S.L.). For ^{1}H NMR, the multiplicity used for assignment is indicated by the following abbreviations: s = singlet, d = doublet, t = triplet, q = quartet, p = pentet, h = sextet, hept = heptet, m = multiplet, br = broad. Signals of protons and carbons were assigned, as far as possible, by using the following two dimensional NMR spectroscopy techniques: [^{1}H, ^{1}H] COSY (Correlation Spectroscopy), [^{1}H, ^{13}C] HSQC (Heteronuclear Single Quantum Coherence) and long range [^{1}H, ^{13}C] HMBC (Heteronuclear Multiple Bond Connectivity). Low resolution (LR) and high resolution (HR) electrospray mass spectral (ES-MS) analyses were acquired by electrospray ionisation (ESI). These were acquired by the EPSRC National Mass Spectrometry Service or within the School of Chemistry, University of St Andrews. GC analyses were obtained on a Shimadzu GC consisting of a Shimadzu AOC-20i auto injector and a Shimadzu GC-2025 gas chromatograph. Analysis was performed using Shimadzu GCsolution v2.41 software and separation was achieved using an Agilent Cyclosil-B column. Optical rotations were measured on a polarimeter Perkin-Elmer 341 at 20 °C or a SEPA-300 (HORIBA, Kyoto, Japan) at 23 °C at a wavelength of λ 589.3 nm in a 1 mL quartz cell (1 dm

length). The concentration is given in g/100 mL. The UPLC-TOFMS data was obtained using a Waters Xevo G2 Q-Tof mass spectrometer with Aquity™ UPLC system

6 Experimental procedures

Ethyl (*R*,*E*)-2-((*tert*-butylsulfinyl)imino)propanoate (42)[11]

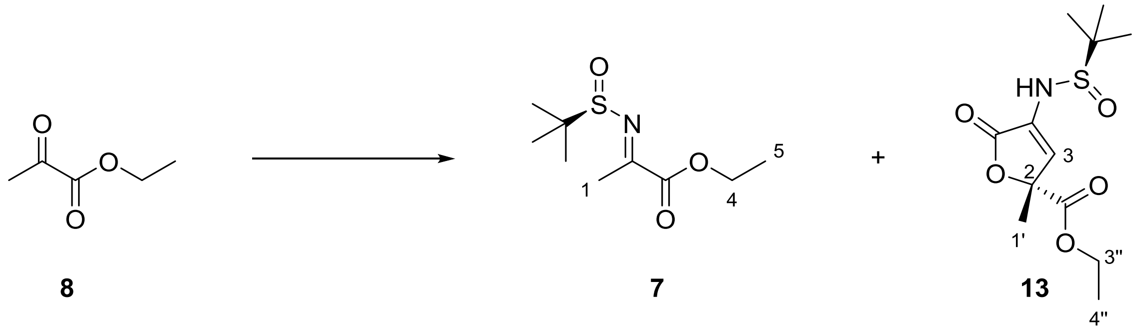

8 **7** **13**

Ethyl pyruvate **8** (4.3 g, 37.1 mmol, 1.5 eq.) was added to a stirred solution of (*R*)-(+)-2-methyl-2-propanesulfinamide (3.0 g, 24.8 mmol, 1.0 eq.) and $Ti(OEt)_4$ (8.5 g, 37.1 mmol, 1.5 eq.) in THF (100 mL) at 65 °C. The reaction was stirred at the same temperature for 4 hours before cooling to room temperature. The crude reaction mixture was poured into brine (100 mL) whilst being vigorously stirred. The resulting suspension was filtered through Celite, and the filter cake was washed with EtOAc (2 × 30 mL). The filtrate was washed with brine (30 mL), and the brine layer was back extracted with EtOAc (3 × 30 mL). The combined organic extracts were dried over $MgSO_4$, filtered, concentrated *in vacuo* and purified *via* the Biotage SP4 (silica-packed SNAP column 180 g; 10-50% EtOAc/hexanes) to give the title product **7** as a pale yellow oil (2.5 g, 45%) and the enamine tautomer as a pale yellow oil (0.8 g, 15%). **7** was isolated in a 3:1 ratio (by analysis of the crude NMR spectrum) to lactone **13** (1.1 g). The NMR analysis of the imine tautomer of **7** was complicated by the existence of rotamers. **^{1}H NMR** (500 MHz, Chloroform-*d*) δ(*major imine-form*) 4.50 – 3.96 (m, 2H, C4-$\underline{H}_2$), 2.46 (d, *J* = 120.8 Hz, 3H, C1-$\underline{H}_3$), 1.37 – 1.32 (m, 3H, C5-$\underline{H}_3$), 1.27 (d, *J* = 27.6 Hz, 9H, $(C\underline{H}_3)_3$); **^{13}C NMR** (126 MHz, Chloroform-*d*) δ(*major imine-form*) 167.8 (C3), 163.6 (C2), 62.4 (d, *J* = 42.6 Hz, C4), 59.4 ($\underline{C}(CH_3)_3$), 25.4 (C1), 22.8 (d, *J* = 37.8 Hz, $(\underline{C}H_3)_3$), 18.5 (C1), 14.1 (d, *J* = 14.9 Hz, C5); **^{1}H NMR** (500 MHz, Chloroform-*d*) δ(*minor enamine-form*) 5.95 (s, 1H, N$\underline{H}$), 5.50 (t, *J* = 1.6 Hz, 1H, C1-$\underline{H}_2$), 5.23 (d, *J* = 1.6 Hz, 1H, C1-$\underline{H}_2$), 4.28 (q, *J* = 7.1, 2H, C4-$\underline{H}_2$), 1.33 (t, *J* = 7.1 Hz, 3H, C5-$\underline{H}_3$), 1.28 (s, 9H, $(C\underline{H}_3)_3$); **^{13}C NMR** (126 MHz, Chloroform-*d*) δ(*minor enamine-form*) 164.0 (C3), 136.2 (C2), 100.7 (C1), 62.4 (C4), 56.7 ($\underline{C}(CH_3)_3$), 22.4 ($(\underline{C}H_3)_3$), 14.3 (C5); ***m/z*** (ES^+) 220.10 ($[M+H]^+$, 100 %); **HRMS** (ES^+) Calcd for $C_9H_{18}NO_3S$ $[M+H]^+$: 220.1002, found 220.0999; $[\alpha]_D^{20}$ = -121.8 (*c* 1.15, $CHCl_3$). (lit. -125.8).[11] Spectroscopic data are in accordance with the literature.[11]

Ethyl (*R*)-4-(((*R*)-*tert*-butylsulfinyl)amino)-2-methyl-5-oxo-2,5-dihydrofuran-2-carboxylate (13)

In $CDCl_3$ at room temperature the title compound **13** exists as a mixture of rotamers. **IR** (KBr) ν_{max}: 2978 (NH), 1769 (C=O), 1748 (C=O), 1651, 1458, 1248, 1126, 1063; **^{1}H NMR** (500 MHz, Chloroform-*d*) δ 6.42 (d, *J* = 2.1 Hz, 1H, C3-H), 5.74 (d, *J* = 4.6 Hz, 1H, NH), 4.22 (qt, *J* = 7.1, 1.8 Hz, 2H, C3''-H_2), 1.72 (d, *J* = 7.3 Hz, 3H, C1'-H_3), 1.29 (s, 9H, $(CH_3)_3$), 1.30 – 1.26 (m, 3H, C4''-H_3); **^{13}C NMR** (126 MHz, Chloroform-*d*) δ 168.5 (d, *J* = 15.8 Hz, CO), 168.0 (C5), 131.1 (d, *J* = 4.1 Hz, C4), 120.3 (d, *J* = 11.9 Hz, C3), 84.7 (d, *J* = 7.0 Hz, C2), 62.6 (d, *J* = 15.4 Hz, C3''), 57.6 (d, *J* = 13.0 Hz, $C(CH_3)_3$), 22.9 (d, *J* = 6.6 Hz, C1'), 22.1 ($(CH_3)_3$), 14.0 (C4''); ***m/z*** (ES^+) 312.09 ($[M+Na]^+$, 100 %); **HRMS** (ES^+) Calcd for $C_{12}H_{19}NO_5SNa$ $[M+Na]^+$: 312.0876, found 312.0869. $[\alpha]_D^{20}$ = -52.9 (*c* 1.0, MeOH).

Ethyl (*R*)-4-(((*R*)-*tert*-butylsulfinyl)amino)-2-isopropyl-5-oxo-2,5-dihydrofuran-2-carboxylate (14)

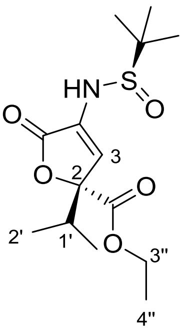

To a solution of *i*-Pr_2NH (1.68 mL, 12.0 mmol, 1.2 eq.) in THF (50 mL) was added *n*-BuLi (5.1 mL, 2.2 M in hexanes, 1.1 eq.) at 0 °C and the mixture was stirred for 30 minutes. The solution was then cooled to -78 °C and **13** (2.20 g, 10.0 mmol, 1.0 eq.) was added. The reaction was stirred for a further 30 minutes at this temperature before the addition of $ZnBr_2$ (4.50 g, 20.0 mmol, 2.0 eq.), followed by ethyl dimethylpyruvate (1.87 g, 13.0 mmol, 1.3 eq.). The reaction was then stirred for 3 hours at -78 °C before the addition of a saturated solution of NH_4Cl (30 mL). The reaction was allowed to slowly warm to room temperature. The mixture was extracted with EtOAc (3 × 30 mL) and the organic layers combined and washed with brine (30 mL), dried over $MgSO_4$, filtered, concentrated *in vacuo* and purified *via* the Biotage SP4 (silica-packed SNAP column 120 g; 20-60% EtOAc/hexanes) to give the title product **14** as a white solid (2.80 g, 88%). **mp** 77-79 °C; **IR** (KBr) ν_{max}: 2965 (NH), 1775 (C=O), 1714 (C=O), 1647, 1366, 1236, 1103, 1051, 1018; **^{1}H NMR** (500 MHz, Chloroform-*d*) δ 6.37 (s, 1H, C3-H), 5.71 (s, 1H, NH), 4.24 (q, *J* = 7.1 Hz, 2H, C3''-H_2), 2.46 (hept, *J* = 6.9 Hz, 1H, C1'-H), 1.34 – 1.21 (m, 12H, $(CH_3)_3$, C4''-H_3), 1.04 (d, *J* = 6.9 Hz, 3H, C2'-H_3), 0.89 (d, *J* = 6.9 Hz, 3H, C2'-H_3); **^{13}C NMR** (126 MHz, Chloroform-*d*) δ 168.5 (CO), 168.3 (C5), 131.3 (C4), 119.2 (C3), 90.7 (C2), 62.5 (C3''), 57.6 ($C(CH_3)_3$), 34.2 (C1'), 22.2 ($(CH_3)_3$), 17.4 (C2'), 15.8 (C2'), 14.3 (C4''); ***m/z*** (ES^+) 318.14 ($[M+H]^+$, 100 %); **HRMS** (ES^+) Calcd for $C_{14}H_{24}NO_5S$ $[M+H]^+$: 318.1370, found 318.1366; $[\alpha]_D^{20}$ = +24.1 (*c* 1.0, MeOH).

Ethyl (*R*)-4-(((*R*)-*tert*-butylsulfinyl)(methyl)amino)-2-isopropyl-5-oxo-2,5-dihydrofuran-2-carboxylate (15)

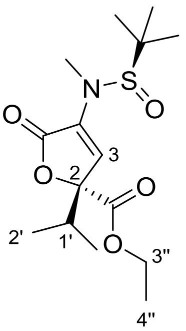

To a solution of **14** (0.98 g, 3.09 mmol, 1.0 eq.) in DMF (12 mL) at -15 °C was added LiHMDS (3.09 mL, 1 M in THF, 1.0 eq.) dropwise and the reaction was stirred for 40 minutes. Iodomethane (0.38 mL, 6.18 mmol, 2.0 eq.) was then added and the reaction was allowed to slowly warm to room temperature before stirring at this temperature for 2 hours. The reaction was diluted with H_2O (15 mL) before being extracted with EtOAc (3 × 20 mL). The organic layers were combined and washed with $Na_2S_2O_3$ (20 mL), brine (20 mL), dried over $MgSO_4$, filtered, concentrated *in vacuo* and purified *via* the Biotage SP4 (silica-packed SNAP column 40 g; 0-30% EtOAc/hexanes) to give the title product **15** as a white solid (0.97 g, 95%). **mp** 70-72 °C; **IR** (KBr) ν_{max}: 2970, 1763 (C=O), 1738 (C=O), 1622, 1248, 1198, 1092, 1038, 1005; **^{1}H NMR** (500 MHz, Chloroform-*d*) δ 6.21 (s, 1H, C3-H), 4.31 – 4.10 (m, 2H, C3''-H_2), 2.94 (s, 3H, NCH_3), 2.45 (hept, *J* = 6.9 Hz, 1H, C1'-H), 1.29 (t, *J* = 7.1 Hz, 3H, C4''-H_3), 1.25 (s, 9H, (CH_3)$_3$), 1.03 (d, *J* = 6.9 Hz, 3H, C2'-H_3), 0.86 (d, *J* = 6.9 Hz, 3H, C2'-H_3); **^{13}C NMR** (126 MHz, Chloroform-*d*) δ 168.7 (CO), 166.9 (C5), 137.0 (C4), 122.7 (C3), 89.3 (C2), 62.5 (C3''), 60.8 ($C(CH_3)_3$), 34.2 (C1'), 29.3 (NCH_3), 23.4 ((CH_3)$_3$), 17.3 (C2'), 15.7 (C2'), 14.3 (C4''); ***m/z*** (ES^+) 332.15 ($[M+H]^+$, 100 %); **HRMS** (ES^+) Calcd for $C_{15}H_{26}NO_5S$ $[M+H]^+$: 332.1526, found 332.1521; $[\alpha]_D^{20}$ = +229.5 (*c* 1.0, MeOH).

6,6-Dimethoxyhexanal (18)[12]

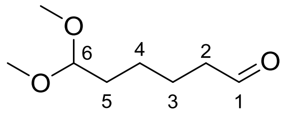

A solution of cyclohexene (5.06 mL, 50.0 mmol, 1.0 eq.) in dry DCM (150 mL)/MeOH (50 mL) at -78 °C was purged with O_2. O_3 was bubbled through the solution until it became a blue colour. The reaction was purged with N_2 until the blue colour disappeared. PTSA (951 mg, 5.00 mmol, 0.1 eq.) was added and the reaction was stirred at room temperature for 1.5 hours before the addition of anhydrous $NaHCO_3$ (16.8 g, 200 mmol, 4.0 eq.). Dimethyl sulfide (6.21 g, 100 mmol, 2.0 eq.) was added after a further 15 minutes and the reaction was stirred at room temperature for 12 hours.

The reaction was concentrated *in vacuo* to approximately 40 mL. The resulting mixture was diluted with DCM (40 mL) and washed with H_2O (3 × 30 mL). The aqueous extracts were combined and extracted with DCM (2 × 20 mL). The organic extracts were combined, washed with brine (30 mL), dried over $MgSO_4$, filtered, concentrated *in vacuo* and purified *via* the Biotage SP4 (silica-packed SNAP column 100 g; 0-40% EtOAc/hexanes) to give the title product **18** as a colourless oil (6.54 g, 82%). **^{1}H NMR** (500 MHz, Chloroform-*d*) δ 9.76 (t, *J* = 1.7 Hz, 1H, C1-H), 4.35 (t, *J* = 5.7 Hz, 1H, C6-H), 3.31 (d, *J* = 2.1 Hz, 6H, $(OCH_3)_2$), 2.44 (td, *J* = 7.3, 1.7 Hz, 2H, C2-H_2), 1.80 – 1.45 (m, 4H, C3-H_2, C5-H_2), 1.48 – 1.26 (m, 2H, C4-H_2); **^{13}C NMR** (126 MHz, Chloroform-*d*) δ 202.7 (C1), 104.4 (C6), 52.9 ($(CH_3)_3$), 44.0 (C2), 32.4 (C5), 24.3 (C4), 22.0 (C3). Spectroscopic data are in accordance with the literature.[12]

(2*E*,4*E*)-10,10-Dimethoxydeca-2,4-diene (20)[13]

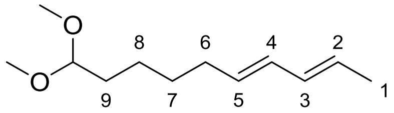

To a solution of diethyl 2-butenylphosphonate **19** (2.96 mL, 15.6 mmol, 1.0 eq.) in DME (60 mL) at -78 °C was added KHMDS (17.2 mmol, 1.1 eq., 1 M in THF) and the reaction was stirred for 30 minutes. A solution of **18** in DME (30 mL) was then added and the reaction was stirred for a further 30 minutes before stirring at 0 °C for 2 hours followed by 2 hours at room temperature. The reaction was diluted with Et_2O (30 mL)/saturated aqueous solution of NH_4Cl (30 mL). The aqueous layer was extracted with Et_2O (3 × 20 mL). The organic extracts were combined, washed with brine (30 mL), dried over $MgSO_4$, filtered, concentrated *in vacuo* and purified *via* the Biotage SP4 (silica-packed SNAP column 80 g; 0-20% EtOAc/hexanes) to give the title product **20** as a colourless oil (1.76 g, 57%, ~8:1 *E*:*Z*). **^{1}H NMR** (500 MHz, Chloroform-*d*) δ 6.05 – 5.93 (m, 2H, C3-H, C4-H), 5.63 – 5.48 (m, 2H, C2-H, C5-H), 4.35 (t, *J* = 5.7 Hz, 1H, C10-H), 3.31 (s, 6H, $(OCH_3)_2$), 2.06 (app. q, *J* = 7.0 Hz, 2H, C6-H_2), 1.72 (d, *J* = 6.1 Hz, 3H, C1-H_3), 1.63 – 1.54 (m, 2H, C9-H_2), 1.46 – 1.29 (m, 4H, C7-H_2, C8-H_2); **^{13}C NMR** (126 MHz, Chloroform-*d*) δ 131.9 (C5), 131.8 (C3), 130.6 (C4), 127.0 (C2), 104.6 (C10), 52.7 ($(OCH_3)_2$), 32.6 (C6), 32.5 (C9), 29.4 (C7), 24.3 (C8), 18.2 (C1); ***m/z*** (ES^+) 216.20 ($[M+NH_4]^+$, 100 %); **HRMS** (ES^+) Calcd for $C_{12}H_{26}NO_2$ $[M+NH_4]^+$: 216.1958, found 216.1954. Spectroscopic data are in accordance with the literature.[13]

(6*E*,8*E*)-Deca-6,8-dienal (21)[14]

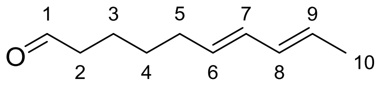

To **20** (1.74 g, 8.77 mmol, 1.0 eq.) in THF (35 mL) was added an aqueous solution of HCl (9.65 mL, 1 N, 1.1 eq.) and the reaction was stirred at room temperature for 12 hours. The reaction was

quenched by the addition of a saturated aqueous solution of $NaHCO_3$ (30 mL). The mixture was extracted with Et_2O (3 × 30 mL) and the organic layers were combined, washed with brine (30 mL), dried over $MgSO_4$, filtered and concentrated *in vacuo*. Title product **21** was isolated as a pale yellow liquid (1.26 g, 94%) which was used in the next step without further purification. **^{1}H NMR** (500 MHz, Chloroform-*d*) δ 9.76 (t, *J* = 1.8 Hz, 1H, C1-H), 6.04 – 5.92 (m, 2H, C7-H, C8-H), 5.64 – 5.45 (m, 2H, C6-H, C9-H), 2.42 (td, *J* = 7.4, 1.8 Hz, 2H, C2-H_2), 2.08 (app. q, *J* = 7.2 Hz, 2H, C5-H_2), 1.72 (d, *J* = 6.6 Hz, 3H, C10-H_3), 1.68 – 1.55 (m, 2H, C3-H_2), 1.47 – 1.37 (m, 2H, C4-H_2); **^{13}C NMR** (126 MHz, Chloroform-*d*) δ 202.7 (C1), 131.5 (C8), 131.0 (C6), 130.8 (C7), 127.2 (C9), 43.8 (C2), 32.2 (C5), 28.9 (C4), 21.6 (C3), 18.0 (C10); ***m/z*** (ES^+) 153.12 ($[M+H]^+$, 100 %); **HRMS** (ES^+) Calcd for $C_{10}H_{17}O$ $[M+H]^+$: 153.1274, found 153.1270. Spectroscopic data are in accordance with the literature.[14]

(2*E*,8*E*,10*E*)-Dodeca-2,8,10-trienal (23)[6]

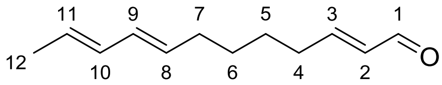

To a solution of (1,3-dioxolan-2-ylmethyl)triphenylphosphonium bromide **22** (368 mg, 0.85 mmol, 1.3 eq.), in anhydrous THF (2 mL) at 0 °C was added ^{t}BuOK (1.05 mL, 1.6 eq., 1 M in THF) dropwise. The mixture was stirred for 30 minutes before the addition of a solution of **21** (100 mg, 0.66 mmol, 1.0 eq.) in anhydrous THF (2.5 mL). The reaction was stirred at 0 °C for 3 hours. A 10% aqueous solution of oxalic acid (5 mL) was added and the reaction was stirred at room temperature for a further 1 hour. The mixture was extracted with Et_2O (4 × 10 mL) and the combined organic layers were washed with a saturated aqueous solution of $NaHCO_3$ (10 mL), brine (10 mL), dried over Na_2SO_4, filtered, concentrated *in vacuo* and purified *via* the Biotage SP4 (silica-packed SNAP column 4 g; 0-2% EtOAc/hexanes) to give the title product **23** as a colourless oil (75 mg, 64%). **^{1}H NMR** (500 MHz, Chloroform-*d*) δ 9.50 (d, *J* = 7.9 Hz, 1H, C1-H), 6.84 (dt, *J* = 15.5, 6.8 Hz, 1H, C3-H), 6.11 (ddt, *J* = 15.5, 7.9, 1.5 Hz, 1H, C2-H), 6.04 – 5.95 (m, 2H, C9-H, C10-H), 5.63 – 5.55 (m, 1H, C11-H), 5.56 – 5.47 (m, 1H, C8-H), 2.39 – 2.29 (m, 2H, C4-H_2), 2.08 (td, *J* = 7.1, 7.1 Hz, 2H, C7-H_2), 1.73 (d, *J* = 6.2 Hz, 3H, C12-H_3), 1.58 – 1.47 (m, 2H, C5-H_2), 1.49 – 1.39 (m, 2H, C6-H_2); **^{13}C NMR** (126 MHz, Chloroform-*d*) δ 194.3 (C1), 158.9 (C3), 133.2 (C2), 131.6 (C8), 131.2 (C10), 130.9 (C9), 127.4 (C11), 32.7 (C7), 32.3 (C4), 29.0 (C6), 27.4 (C5), 18.2 (C12). Spectroscopic data are in accordance with the literature.[6]

2-Methyl-1,2,4a,5,6,7,8,8a-octahydronaphthalene-1-carbaldehyde (24)[6,7]

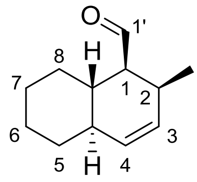

24a
(1*S*, 2*S*, 4a*R*, 8a*S*)

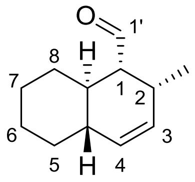

24b
(1*R*, 2*R*, 4a*S*, 8a*R*)

To **23** (1.25 g, 7.01 mmol, 1.0 eq.; 85% *E*,*E*-diene geometry) in a 2% v/v solution of H_2O/MeCN (23 mL) at -5 °C was added a solution of (2*S*,5*S*)-5-benzyl-2-*tert*butyl-3-methylimidazolidin-4-one trifluoromethanesulfonic acid salt (1.4 mL, 0.2 eq., 1M in MeCN). The reaction was stirred at -5 °C for 48 hours before being concentrated *in vacuo* and purified *via* the Biotage SP4 (silica-packed SNAP column 4 g; 0-2% EtOAc/hexanes) to give **24a** (0.69 g, 65%,[1] *dr* 4:1,[2] 87% ee[3]) as a colourless oil. [1]Yields reported based on the conversion of the *E*,*E*-diene substrate to IMDA product. [2]Diastereomeric ratios were determined by ^{1}H NMR analysis. [3]Enantiomeric purity was determined by chiral GC analysis of the corresponding alcohols (**S2a**).

To **23** (1.45 g, 8.13 mmol, 1.0 eq.; 85% *E*,*E*-diene geometry) in a 2% v/v solution of H_2O/MeCN (27 mL) at -5 °C was added a solution of (2*R*,5*R*)-5-benzyl-2-*tert*butyl-3-methylimidazolidin-4-one trifluoromethanesulfonic acid salt (1.63 mL, 0.2 eq., 1M in MeCN). The reaction was stirred at -5 °C for 48 hours before being concentrated *in vacuo* and purified *via* the Biotage SP4 (silica-packed SNAP column 4 g; 0-2% EtOAc/hexanes) to give **24b** (0.84 g, 68%,[1] *dr* 4:1,[2] 84% ee[3]) as a colourless oil. [1]Yields reported based on the conversion of the *E*,*E*-diene substrate to IMDA product. [2]Diastereomeric ratios were determined by ^{1}H NMR analysis. [3]Enantiomeric purity was determined by chiral GC analysis of the corresponding alcohols (**S2b**).

^{1}H NMR (500 MHz, Chloroform-*d*) δ 9.75 (d, *J* = 4.3 Hz, 1H, C1'-H), 5.54 (ddd, *J* = 9.9, 4.5, 2.6 Hz, 1H, C3-H), 5.42 (d, *J* = 9.9 Hz, 1H, C4-H), 2.66 – 2.51 (m, 1H, C2-H), 2.38 (ddd, *J* = 11.4, 6.0, 4.3 Hz, 1H, C1-H), 1.85 – 1.71 (m, 4H, C5-H_2, C6-H_2, C7-H_2, C8-H_2), 1.74 – 1.64 (m, 1H, C4a-H), 1.67 – 1.58 (m, 1H, C8a-H), 1.39 – 1.29 (m, 2H, C6-H_2, C7-H_2), 1.17 – 1.07 (m, 1H, C5-H_2), 1.04 (d, *J* = 7.1 Hz, 3H, CH_3), 1.02 – 0.93 (m, 1H, C8-H_2); **^{13}C NMR** (126 MHz, Chloroform-*d*) δ 207.2 (C1'), 131.4 (C4), 131.1 (C3), 55.6 (C1), 42.2 (C4a), 35.7 (C8a), 33.1 (C5), 31.9 (C2), 30.3 (C8), 26.7 (CH_2), 26.6 (CH_2), 17.1 (CH_3). Spectroscopic data are in accordance with the literature.[6],[7]

2-Methyl-1,2,4a,5,6,7,8,8a-octahydronaphthalen-1-yl)methanol (S2)[7]

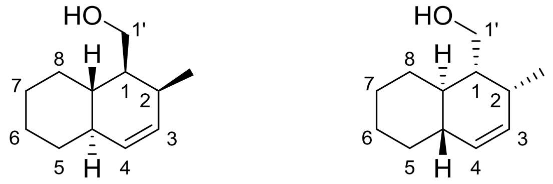

S2a
(1*S*, 2*S*, 4a*R*, 8a*S*)

S2b
(1*R*, 2*R*, 4a*S*, 8a*R*)

$NaBH_4$ (1.5 eq.) was added slowly to a solution of **24a** (40 mg, 0.22 mmol, 1.0 eq.) or **24b** (83 mg, 0.47 mmol, 1.0 eq.) in EtOH (5 mL/mmol) at 0 °C, and the reaction was stirred for 1 hour. The reaction was quenched by addition of a saturated aqueous solution of NH_4Cl and extracted with Et_2O. The organic layers were combined, washed with H_2O, brine, dried over Na_2SO_4, filtered, concentrated *in vacuo* and purified *via* the Biotage SP4 (silica-packed SNAP column; 0-10% EtOAc/hexanes) to give **S2a** (35 mg, 88%, dr 4:1) and **S2b** (77 mg, 91%, dr 4:1) as white solids. **S2a** - Chiral GC analysis Agilent Cyclosil-B (length: 30 m, thickness: 0.250 mm, film thickness: 0.25 µm), carrier gas: He, linear velocity: 40 cm/sec, temperature: 140 °C, tR minor (1*R,* 2*R*, 4a*S*, 8a*R*) 23.0 min, tR major (1*S,* 2*S*, 4a*R*, 8a*S*) 23.6 min, 87% ee; **S2b** - Chiral GC analysis Agilent Cyclosil-B (length: 30 m, thickness: 0.250 mm, film thickness: 0.25 µm), carrier gas: He, linear velocity: 40 cm/sec, temperature: 140 °C, tR major (1*R,* 2*R*, 4a*S*, 8a*R*) 23.0 min, tR minor (1*S,* 2*S*, 4a*R*, 8a*S*) 23.8 min, 84% ee; **^{1}H NMR** (400 MHz, Chloroform-*d*) δ 5.59 (ddd, *J* = 9.9, 4.8, 2.6 Hz, 1H, C3-H), 5.36 (d, *J* = 9.9 Hz, 1H, C4-H), 3.83 (dd, *J* = 10.7, 5.4 Hz, 1H, C1'-H_2), 3.53 (dd, *J* = 10.7, 9.3 Hz, 1H, C1'-H_2), 2.49 – 2.33 (m, 1H, C2-H), 1.81 – 1.64 (m, 6H, C1-H, C4a-H, C5-H_2, C6-H_2, C7-H_2, C8-H_2), 1.36 – 1.18 (m, 2H, C6-H_2, C7-H_2), 1.16 – 1.06 (m, 1H, C8a-H), 1.06 – 0.95 (m, 2H, C5-H_2, C8-H_2), 0.92 (d, *J* = 7.0 Hz, 3H, CH_3); **^{13}C NMR** (101 MHz, Chloroform-*d*) δ 132.4 (C3), 131.4 (C4), 63.2 (C1'), 44.3 (C1), 43.7 (C4a), 37.8 (C8a), 33.4 (C5), 31.6 (C2), 29.4 (C8), 26.9 (CH_2), 26.7 (CH_2), 15.6 (CH_3). Spectroscopic data is in accordance with the literature.[7]

S-(*tert*-Butyl) 3-hydroxy-3-(2-methyl-1,2,4a,5,6,7,8,8a-octahydronaphthalen-1-yl)propanethioate (25)

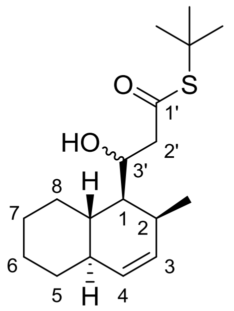

25a
(1*S*, 2*S*, 4a*R*, 8a*S*)

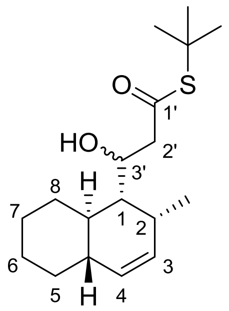

25b
(1*R*, 2*R*, 4a*S*, 8a*R*)

To a solution of *i*-Pr_2NH (1.3 eq.) in anhydrous THF (4 mL/mmol) was added *n*-BuLi (1.2 eq., 2.3 M in hexanes) at 0 °C and the mixture was stirred for 30 minutes. The solution was then cooled to -78 °C and a solution of *S-tert*-butyl-thioacetate (1.5 eq.) in anhydrous THF (2 mL/mmol) was added. After a further 30 minutes at -78 °C, a solution of **24a** (0.34 g, 1.9 mmol, 1.0 eq.) or **24b** (0.5 g, 2.8 mmol, 1.0 eq.) in anhydrous THF (2 mL/mmol) was added, and the reaction was stirred for 2 hours. The reaction was quenched by the addition of a saturated aqueous solution of NH_4Cl, warmed to room temperature and extracted with EtOAc. The organic layers were combined, washed with H_2O, brine, dried over Na_2SO_4, filtered, concentrated *in vacuo* and purified *via* the Biotage SP4 (silica-packed SNAP column; 0-5% EtOAc/hexanes) to give **25a** (0.38 g, 66%) and **25b** (0.60 g, 69%) as colourless oils. **25a** and **25b** were obtained as an inconsequential 7:3 mixture of diastereomers. **IR** (thin film) ν_{max}: 2920, 2851, 1680, 1670, 1456, 1364, 1030; **^{1}H NMR** (400 MHz, Chloroform-*d*) δ(*major diastereomer*) 5.53 (ddd, *J* = 9.9, 4.9, 2.5 Hz, 1H, C3-H), 5.33 (d, *J* = 9.9 Hz, 1H, C4-H), 4.25 – 4.11 (m, 1H, C3'-H), 2.79 (dd, *J* = 15.8, 9.3 Hz, 1H, C2'-H_2), 2.70 (dd, *J* = 15.8, 2.9 Hz, 1H, C2'-H_2), 2.52 (d, *J* = 3.8 Hz, 1H, OH), 2.33 – 2.22 (m, 1H, C2-H), 2.08 – 1.97 (m, 1H, C8-H_2), 1.85 – 1.67 (m, 4H, C4a-H, C5-H_2, C6-H_2, C7-H_2), 1.66 – 1.58 (m, 1H, C1-H), 1.47 (s, 9H, $(CH_3)_3$), 1.34 – 1.23 (m, 3H, C8a-H, C6-H_2, C7-H_2), 1.18 – 1.03 (m, 2H, C5-H_2, C8-H_2), 0.99 (d, *J* = 7.0 Hz, 3H, CH_3); **^{13}C NMR** (101 MHz, Chloroform-*d*) δ(*major diastereomer*) 201.1 (C1'), 132.4 (C3), 131.5 (C4), 70.7 (C3'), 50.4 (C2'), 46.9 (C1), 44.5 (C4a), 38.4 (C8a), 33.9 (C2), 33.7 (C5), 31.3 (C8), 29.9 ($(CH_3)_3$), 27.2 (CH_2), 26.7 (CH_2), 17.0 (CH_3); **^{1}H NMR** (400 MHz, Chloroform-*d*) δ(*minor diastereomer*) 5.51 (ddd, *J* = 9.9, 5.0, 2.6 Hz, 1H, C3-H), 5.32 (d, *J* = 9.9 Hz, 1H, C4-H), 4.46 – 4.33 (m, 1H, C3'-H), 2.81 (dd, *J* = 15.7, 10.0 Hz, 1H, C2'-H_2), 2.59 (dd, *J* = 15.7, 2.5 Hz, 1H, C2'-H_2), 2.39 (d, *J* = 3.2 Hz, 1H, OH), 2.36 – 2.25 (m, 1H, C2-H), 2.09 – 1.99 (m, 1H, C8-H_2), 1.87 – 1.64 (m, 4H, C4a-H, C5-H_2, C6-H_2, C7-H_2), 1.47 (s, 9H, $(CH_3)_3$), 1.45 – 1.41 (m, 1H, C1-H), 1.42 – 1.34 (m, 1H, C8a-H), 1.34 – 1.21 (m, 2H, C6-H_2, C7-H_2), 1.14 – 1.02 (m, 1H, C5-H_2), 1.07 (d, *J* = 7.0 Hz, 3H, CH_3), 1.04 – 0.94 (m, 1H, C8-H_2); **^{13}C NMR** (101 MHz, Chloroform-*d*) δ(*minor diastereomer*) 201.2 (C1'), 132.8 (C3), 130.9 (C4), 68.1 (C3'), 49.3 (C2'), 46.4 (C1), 44.6 (C4a), 36.9

(C8a), 33.5 (C5), 31.6 (C2), 29.9 ((C̲H$_3$)$_3$), 29.3 (C8), 26.9 (C̲H$_2$), 26.8 (C̲H$_2$), 18.3 (C̲H$_3$); ***m/z*** (ES$^+$) 333.19 ([M+Na]$^+$, 100 %); **HRMS** (ES$^+$) Calcd for $C_{18}H_{30}O_2SNa$ [M+Na]$^+$: 333.1864, found 333.1855.

***S*-(*tert*-Butyl) 3-(2-methyl-1,2,4a,5,6,7,8,8a-octahydronaphthalen-1-yl)-3-oxopropanethioate (17)**

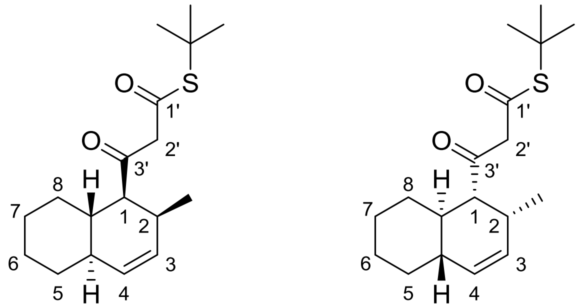

17a (1*S*, 2*S*, 4a*R*, 8a*S*) **17b** (1*R*, 2*R*, 4a*S*, 8a*R*)

To a solution of **25a** (0.29 g, 0.93 mmol, 1.0 eq.) or **25b** (0.58 g, 1.88 mmol, 1.0 eq.) in DCM (10 mL/mmol) at room temperature was added Dess-Martin Periodinane (1.2 eq.). The reaction was stirred for 2 hours before being quenched by addition of a 1:1 (v:v) saturated sodium thiosulfate: saturated sodium bicarbonate solution (10 mL/mmol). The mixture was extracted with DCM. The organic extracts were combined, washed with brine, dried over Na_2SO_4, filtered, concentrated *in vacuo* and purified *via* the Biotage SP4 (silica-packed SNAP column; 0-1% EtOAc/hexanes) to give **17a** (0.23 g, 79%) and **17b** (0.48 g, 82%). In $CDCl_3$ at room temperature the title compound exists as a (3 : 1) *keto* : *enol* mixture. **IR** (thin film) ν_{max}: 2920, 2851, 1717, 1670 (C=O), 1609, 1071; **^{1}H NMR** (500 MHz, Chloroform-*d*) δ(*major keto-form*) 5.54 (ddd, *J* = 10.0, 4.6, 2.6 Hz, 1H, C3-H̲), 5.39 (dd, *J* = 10.0, 1.8 Hz, 1H, C4-H̲), 3.63 – 3.42 (m, 2H, C2'-H̲$_2$), 2.92 (dd, *J* = 11.3, 5.8 Hz, 1H, C1-H̲), 2.63 – 2.51 (m, 1H, C2-H̲), 1.93 – 1.83 (m, 1H, C8-H̲$_2$), 1.78 – 1.69 (m, 3H, C5-H̲$_2$, C6-H̲$_2$, C7-H̲$_2$), 1.69 – 1.64 (m, 1H, C4a-H̲), 1.47 (s, 9H, (CH̲$_3$)$_3$), 1.47 – 1.44 (m, 1H, C8a-H̲), 1.36 – 1.21 (m, 2H, C5-H̲$_2$, C6-H̲$_2$), 1.12 – 1.01 (m, 1H, C7-H̲$_2$), 0.82 (d, *J* = 7.1 Hz, 2H, CH̲$_3$), 0.81 – 0.69 (m, 1H, C8-H̲$_2$); **^{13}C NMR** (126 MHz, Chloroform-*d*) δ(*major keto-form*) 203.6 (C3'), 192.3 (C1'), 131.4 (C4), 130.6 (C3), 59.4 (C2'), 56.0 (C1), 49.2 (C), 42.2 (C4a), 36.3 (C8a), 33.2 (C5), 31.6 (C2), 29.8 ((C̲H$_3$)$_3$), 29.7 (C8), 26.8 (C6), 26.6 (C7), 17.8 (C̲H$_3$); **^{1}H NMR** (500 MHz, Chloroform-*d*) δ(*minor enol-form*) 12.96 (d, *J* = 1.3 Hz, 1H, OH̲), 5.57 – 5.49 (m, 1H, C3-H̲), 5.39 – 5.35 (m, 1H, C4-H̲), 5.32 (s, 1H, C2'-H̲), 2.40 – 2.32 (m, 1H, C2-H̲), 2.20 (ddd, *J* = 11.0, 5.6, 1.0 Hz, 1H, C1-H̲), 1.93 – 1.83 (m, 1H, C8-H̲$_2$), 1.77 – 1.68 (m, 3H, C5-H̲$_2$, C6-H̲$_2$, C7-H̲$_2$), 1.69 – 1.64 (m, 1H, C4a-H̲), 1.51 (s, 9H, (CH̲$_3$)$_3$), 1.46 – 1.43 (m, 1H, C8a-H̲), 1.36 – 1.21 (m, 2H, C5-H̲$_2$, C6-H̲$_2$), 1.13 – 1.01 (m, 1H, C7-H̲$_2$), 0.99 (d, *J* = 7.1 Hz, 3H, CH̲$_3$), 0.81 – 0.69 (m, 1H, C8-H̲$_2$); **^{13}C NMR** (126 MHz, Chloroform-*d*) δ(*minor enol-form*) 196.10 (C1'), 178.15 (C3'), 131.62 (C4), 130.85 (C3), 101.60 (C2'), 49.84 (C1), 48.29 (C), 43.01 (C4a), 36.40 (C8a), 35.05 (C2), 33.29 (C5), 30.36 ((C̲H$_3$)$_3$), 30.19 (C8),

26.82 (C6), 26.70 (C7), 18.03 (C̲H3); ***m/z*** (ES+) 331.17 ([M+Na]+, 100 %); **HRMS** (ES+) Calcd for $C_{18}H_{28}O_2SNa$ [M+Na]+: 331.1708, found 331.1698.

Ethyl (2*S*,4*S*)-2-*iso*propyl-4-(*N*-methyl-3-((1*S*,2*S*,4a*R*,8a*S*)-2-methyl-1,2,4a,5,6,7,8,8a-octahydronaphthalen-1-yl)-3-oxopropanamido)-5-oxotetrahydrofuran-2-carboxylate (26a)

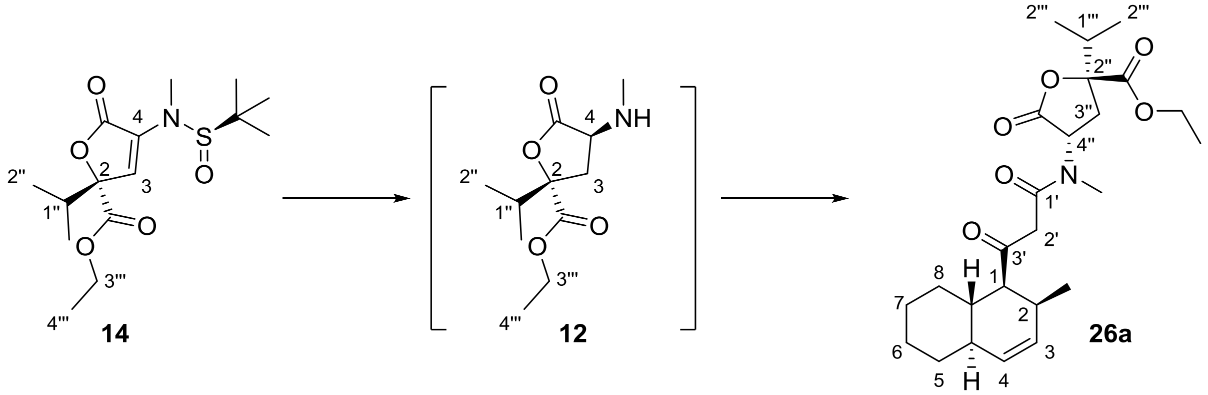

To a solution of **14** (0.32 g, 0.97 mmol, 1.0 eq.) in THF (2.5 mL) at 0 °C was added HCl (0.97 mL, 4 eq., 4 N in dioxane). The reaction was stirred for 10 minutes at this temperature before the addition of a solution of $NaBH_3CN$ (0.18 g, 2.92 mmol, 3.0 eq.) in MeOH (5 mL). The reaction was stirred for a further 1 hour at 0 °C before being concentrated *in vacuo*. The residue was partitioned between a saturated aqueous solution of $NaHCO_3$ (5 mL) and EtOAc (5 mL). The aqueous layer was extracted with EtOAc (3 × 5 mL) and the combined organic extracts were washed with brine (10 mL), dried over Na_2SO_4, filtered and concentrated *in vacuo* to yield the crude free amine **12**. **^{1}H NMR** (500 MHz, Chloroform-*d*) δ 4.25 (qd, *J* = 7.1, 2.2 Hz, 2H, C3'''-H̲2), 3.54 (dd, *J* = 11.5, 8.4 Hz, 1H, C4-H̲), 2.74 (dd, *J* = 12.8, 8.4 Hz, 1H, C3-H̲2), 2.46 (s, 3H, NHCH̲3), 2.25 (hept, *J* = 6.9 Hz, 1H, C1''-H̲), 2.05 (dd, *J* = 12.8, 11.5 Hz, 1H, C3-H̲2), 1.78 (br s, 1H, NH̲CH3), 1.30 (t, *J* = 7.1 Hz, 3H, C4'''-H̲3), 1.02 (d, *J* = 6.9 Hz, 3H, C2'-H̲3), 0.99 (d, *J* = 6.9 Hz, 1H, C2'-H̲3); **^{13}C NMR** (126 MHz, Chloroform-*d*) δ 175.9 (C1), 171.2 (C2), 87.3 (C2), 62.2 (C3'''), 58.1 (C4), 35.9 (C3), 34.4 (NC̲H3), 34.3 (C1''), 17.0 (C2'), 16.4 (C2'), 14.3 (C4''').
To a solution of **12** in anhydrous THF (12 mL) at 0 °C was added **17a** (0.27 g, 0.88 mmol, 0.9 eq.) followed by Et_3N (0.54 mL, 3.89 mmol, 4.0 eq.) and CF_3CO_2Ag (0.43 g, 1.95 mmol, 2.0 eq.). The reaction was then stirred for 1 hour at 0 °C followed by 1 hour at room temperature. The reaction was concentrated *in vacuo* and purified *via* the Biotage SP4 (silica-packed SNAP column 12 g; 0-40% EtOAc/hexanes) to give **26a** as a pale yellow oil (0.30 g, 76%). **IR** (thin film) ν_{max}: 2963, 2922, 2853, 1786, 1734 (C=O), 1717, 1647 (C=O), 1260, 1022; **^{1}H NMR** (500 MHz, Chloroform-*d*) δ 5.55 (ddd, *J* = 9.9, 4.7, 2.6 Hz, 1H, C3-H̲), 5.39 (d, *J* = 9.9 Hz, 1H, C4-H̲), 4.76 (dd, *J* = 11.5, 9.1 Hz, 1H, C4''-H̲), 4.33 – 4.19 (m, 2H, CO2CH̲2CH3), 3.60 (d, *J* = 15.1 Hz, 1H, C2'-H̲2), 3.53 (d, *J* = 15.1 Hz, 1H, C2'-H̲2), 3.02 (s, 3H, NCH̲3), 3.00 – 2.93 (m, 1H, C1-H̲), 2.68 (dd, *J* = 12.8, 9.1 Hz, 1H, C3''-H̲2), 2.65 – 2.57 (m, 1H, C2-H̲), 2.48 (dd, *J* = 12.8, 11.5 Hz, 1H, C3''-H̲2), 2.39 – 2.30 (m, 1H, C1'''-H̲), 1.93 – 1.82 (m, 1H, C8-H̲2), 1.79 –

1.66 (m, 4H, C4a-H, C5-H_2, C6-H_2, C7-H_2), 1.52 – 1.42 (m, 1H, C8a-H), 1.37 – 1.27 (m, 2H, C6-H_2, C7-H_2), 1.32 (t, *J* = 7.2 Hz, 3H, $CO_2CH_2CH_3$), 1.31 – 1.22 (m, 1H, C8-H_2), 1.09 – 0.99 (m, 1H, C5-H_2), 1.05 (d, *J* = 7.1 Hz, 3H, C2′′′-H_3), 1.03 (d, *J* = 6.8 Hz, 3H, C2′′′-H_3), 0.83 (d, *J* = 7.1 Hz, 3H, CH_3); **^{13}C NMR** (126 MHz, Chloroform-*d*) δ 205.7 (C3′), 172.1 (C1′′), 171.0 (CO_2Et), 167.2 (C1′), 131.4 (C4), 130.5 (C3), 87.2 (C2′′), 62.4 ($CO_2CH_2CH_3$), 56.8 (C4′′), 55.8 (C1), 50.3 (C2′), 42.1 (C4a), 36.3 (C8a), 35.6 (NCH_3), 34.2 (C1′′′), 33.2 (C5), 31.7 (C2), 31.4 (C3′′), 29.6 (C8), 26.7 (CH_2), 26.6 (CH_2), 17.8 (CH_3), 17.0 (C2′′′), 16.5 (C2′′′), 14.3 ($CO_2CH_2CH_3$); ***m/z*** (ES$^+$) 470.25 ([M+Na]$^+$, 100 %); **HRMS** (ES$^+$) Calcd for $C_{25}H_{37}O_6NNa$ [M+Na]$^+$: 470.2513, found 470.2507.

Ethyl (2*S*,4*S*)-2-*iso*propyl-4-(*N*-methyl-3-((1*R*,2*R*,4a*S*,8a*R*)-2-methyl-1,2,4a,5,6,7,8,8a-octahydronaphthalen-1-yl)-3-oxopropanamido)-5-oxotetrahydrofuran-2-carboxylate (26b)

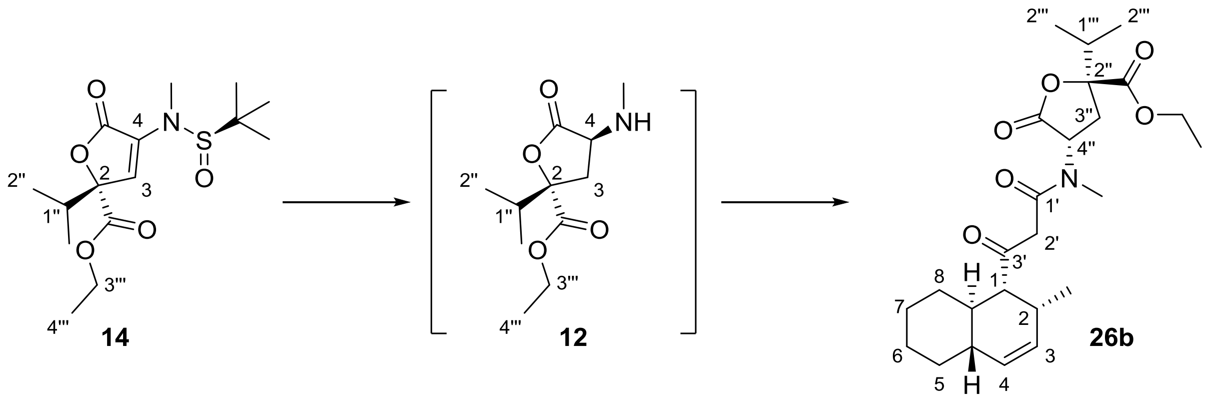

To a solution of **14** (0.31 g, 0.95 mmol, 1.0 eq.) in THF (2.5 mL) at 0 °C was added HCl (0.95 mL, 4 eq., 4 N in dioxane). The reaction was stirred for 10 minutes at this temperature before the addition of a solution of $NaBH_3CN$ (0.18 g, 2.85 mmol, 3.0 eq.) in MeOH (5 mL). The reaction was stirred for a further 1 hour at 0 °C before being concentrated *in vacuo*. The residue was partitioned between a saturated aqueous solution of $NaHCO_3$ (5 mL) and EtOAc (5 mL). The aqueous layer was extracted with EtOAc (3 × 5 mL) and the combined organic extracts were washed with brine (10 mL), dried over Na_2SO_4, filtered and concentrated *in vacuo* to yield the crude free amine **12**. **^{1}H NMR** (500 MHz, Chloroform-*d*) δ 4.25 (qd, *J* = 7.1, 2.2 Hz, 2H, C3′′′-H_2), 3.54 (dd, *J* = 11.5, 8.4 Hz, 1H, C4-H), 2.74 (dd, *J* = 12.8, 8.4 Hz, 1H, C3-H_2), 2.46 (s, 3H, NHCH_3), 2.25 (hept, *J* = 6.9 Hz, 1H, C1′′-H), 2.05 (dd, *J* = 12.8, 11.5 Hz, 1H, C3-H_2), 1.78 (br s, 1H, NHCH_3), 1.30 (t, *J* = 7.1 Hz, 3H, C4′′′-H_3), 1.02 (d, *J* = 6.9 Hz, 3H, C2′-H_3), 0.99 (d, *J* = 6.9 Hz, 1H, C2′-H_3); **^{13}C NMR** (126 MHz, Chloroform-*d*) δ 175.9 (C1), 171.2 (C2), 87.3 (C2), 62.2 (C3′′′), 58.1 (C4), 35.9 (C3), 34.4 (NCH_3), 34.3 (C1′′), 17.0 (C2′), 16.4 (C2′), 14.3 (C4′′′). To a solution of **12** in anhydrous THF (12 mL) at 0 °C was added **17b** (0.26 g, 0.86 mmol, 0.9 eq.) followed by Et_3N (0.53 mL, 3.82 mmol, 4.0 eq.) and CF_3CO_2Ag (0.42 g, 1.91 mmol, 2.0 eq.). The reaction was then stirred for 1 hour at 0 °C followed by 1 hour at room temperature. The reaction was concentrated *in vacuo* and purified *via* the Biotage SP4 (silica-packed SNAP column 12 g; 0-40%

EtOAc/hexanes) to give **26b** as a pale yellow oil (0.27 g, 71%). **IR** (thin film) ν $_{max}$: 2924, 2853, 1790, 1734 (C=O), 1717, 1647 (C=O), 1624, 1261, 1022; **^{1}H NMR** (500 MHz, Chloroform-*d*) δ 5.55 (ddd, *J* = 9.9, 4.7, 2.6 Hz, 1H, C3-H), 5.39 (d, *J* = 9.9 Hz, 1H, C4-H), 5.01 (dd, *J* = 11.6, 9.1 Hz, 1H, C4''-H), 4.33 – 4.20 (m, 2H, $CO_2C\underline{H}_2CH_3$), 3.59 (s, 2H, C2'-$\underline{H}_2$), 2.99 (s, 3H, $NC\underline{H}_3$), 2.97 – 2.91 (m, 1H, C1-H), 2.71 (dd, *J* = 12.9, 9.1 Hz, 1H, C3''-$\underline{H}_2$), 2.66 – 2.57 (m, 1H, C2-H), 2.42 (dd, *J* = 12.9, 11.6 Hz, 1H, C3''-$\underline{H}_2$), 2.38 – 2.30 (m, 1H, C1'''-H), 1.90 – 1.81 (m, 1H, C8-$\underline{H}_2$), 1.79 – 1.65 (m, 4H, C4a-H, C5-$\underline{H}_2$, C6-$\underline{H}_2$, C7-$\underline{H}_2$), 1.53 – 1.42 (m, 1H, C8a-H), 1.32 (t, *J* = 7.1 Hz, 3H, $CO_2CH_2C\underline{H}_3$), 1.37 – 1.27 (m, 3H, C6-$\underline{H}_2$, C7-$\underline{H}_2$, C8-$\underline{H}_2$), 1.05 (d, *J* = 6.9 Hz, 3H, C2'''-$\underline{H}_3$), 1.09 – 0.99 (m, 1H, C5-$\underline{H}_2$), 1.03 (d, *J* = 6.8 Hz, 3H, C2'''-$\underline{H}_3$), 0.84 (d, *J* = 7.1 Hz, 3H, $C\underline{H}_3$); **^{13}C NMR** (126 MHz, Chloroform-*d*) δ 205.7 (C3'), 172.3 (C1''), 170.9 ($\underline{C}O_2Et$), 167.4 (C1'), 131.4 (C4), 130.5 (C3), 87.1 (C2''), 62.4 ($CO_2\underline{C}H_2CH_3$), 55.9 (C4'', C1), 50.4 (C2'), 42.2 (C4a), 36.4 (C8a), 34.7 ($N\underline{C}H_3$), 34.3 (C1'''), 33.2 (C5), 31.7 (C2), 31.5 (C3''), 29.7 (C8), 26.8 ($\underline{C}H_2$), 26.6 ($\underline{C}H_2$), 17.8 ($\underline{C}H_3$), 17.0 (C2'''), 16.4 (C2'''), 14.3 ($CO_2CH_2\underline{C}H_3$); ***m/z*** (ES^{+}) 470.25 ([M+Na]$^{+}$, 100 %); **HRMS** (ES^{+}) Calcd for $C_{25}H_{37}O_6NNa$ [M+Na]$^{+}$: 470.2513, found 470.2506.

(1*S*,2*S*,4a*R*,8a*S*,5'*S*,7'*S*) – JBIR-22 (2a)

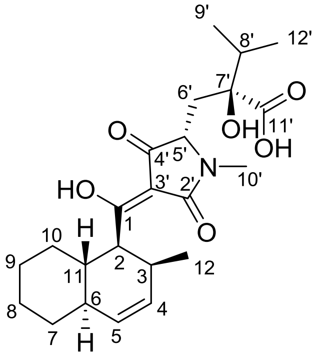

To a solution of **26a** (226 mg, 0.50 mmol, 1.0 eq.) in THF (10 mL) at 0 °C was added ^{t}BuOK (62 mg, 0.56 mmol, 1.1 eq.) and the reaction was stirred for 1 hour. The reaction was slowly warmed to room temperature and stirred at this temperature for 1 hour. The reaction was concentrated *in vacuo* and the residue partitioned between DCM (10 mL) and an aqueous solution of HCl (10 mL, 1N). The aqueous layer was separated and extracted with DCM (3 × 10 mL). The organic extracts were combined, washed with an aqueous solution of HCl (5 mL, 1 N), brine (5 mL), dried over Na_2SO_4, filtered and concentrated *in vacuo* to give JBIR-22 ethyl ester **S3a** as a colourless oil (226 mg, 100%), which was used in the next step without further purification. To a solution of **S3a** (215 mg, 0.48 mmol, 1.0 eq.) in EtOH (4 mL) was added an aqueous solution of NaOH (3 mL, 2N) and the reaction was heated to 110 °C under microwave irradiation for 20 minutes. The reaction was diluted with an aqueous solution of HCl (5 mL, 1N) and extracted with DCM (3 × 5 mL). The organic extracts were combined, washed with brine (10 mL), dried over Na_2SO_4, filtered, concentrated *in vacuo* and

purified *via* the Biotage SP4 (Reverse-phase silica-packed SNAP column 4 g; 20-100% H_2O/(MeOH:MeCN)) to give the title product **2a** as an orange solid (143 mg, 71%). A portion of **2a** was treated with Et_2N to form the salt as a colourless amorphous solid. Spectroscopic data was obtained for the JBIR-22 **2a**-Et_2N salt. $[\alpha]_D^{23}$= +75.0 (*c* 0.1, MeOH); **IR** (thin film) ν $_{max}$: 2920, 2849, 1645, 1616, 1570, 1447, 1387, 1233, 1032, 999, 891, 779; **^{1}H NMR** (500 MHz, Acetone-d_6) δ 5.54 (ddd, *J* = 9.8, 4.5, 2.5 Hz, 1H, C4-H), 5.30 (d, *J* = 9.8 Hz, 1H, C5-H), 3.90 (dd, *J* = 11.5, 5.8 Hz, 1H, C2-H), 3.41 (dd, *J* = 10.0, 1.3 Hz, 1H, C5'-H), 2.73 (s, 3H, C10'-H_3), 2.70 – 2.59 (m, 1H, C3-H), 2.31 (dd, *J* = 13.7, 1.3 Hz, 1H, C6'-H_2), 2.04 – 1.97 (m, 1H, C8'-H), 2.00 – 1.91 (m, 1H, C10-H_2), 1.73 – 1.70 (m, 1H, C7-H_2), 1.70 – 1.67 (m, 1H, C6-H), 1.67 – 1.65 (m, 2H, C8-H_2, C9-H_2), 1.62 (dd, *J* = 13.7, 10.0 Hz, 1H, C6'-H_2), 1.52 – 1.38 (m, 1H, C11-H), 1.30 – 1.28 (m, 2H, C8-H_2, C9-H_2), 1.09 – 0.97 (m, 1H, C7-H_2), 0.91 (d, *J* = 6.6 Hz, 3H, C9'-H_3), 0.90 (d, *J* = 6.6 Hz, 3H, C12'-H_3), 0.77 (d, *J* = 7.2 Hz, 3H, C12-H_3), 0.75 – 0.63 (m, 1H, C10-H_2); **^{13}C NMR** (126 MHz, Acetone-d_6) δ 196.3 (C1), 196.1 (C4'), 180.9 (C11'), 174.7 (C2'), 133.6 (C4), 131.1 (C5), 101.9 (C3'), 80.0 (C7'), 63.3 (C5'), 51.0 (C2), 43.4 (C6), 37.4 (C11), 37.3 (C6'), 36.6 (C8'), 34.4 (C7), 32.2 (C3), 31.1 (C10), 27.7 (C9), 27.6 (C8), 26.9 (C10'), 18.8 (C9'), 18.4 (C12), 16.9 (C12'). ***m/z*** (ES^-) 418.22 ($[M-H]^-$, 100 %); **HRMS** (ES^-) Calcd for $C_{23}H_{32}O_6N$ $[M-H]^-$: 418.2235, found 418.2226.

(1*R*,2*R*,4a*S*,8a*R*,5'*S*,7'*S*) – JBIR-22 (2b)

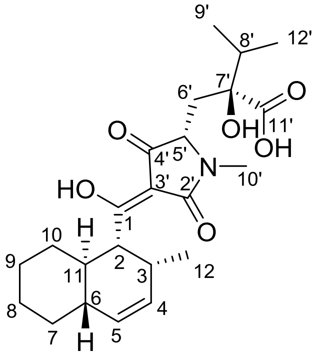

To a solution of **26b** (121 mg, 0.27 mmol, 1.0 eq.) in THF (5 mL) at 0 °C was added ^{t}BuOK (34 mg, 0.30 mmol, 1.1 eq.) and the reaction was stirred for 1 hour. The reaction was slowly warmed to room temperature and stirred at this temperature for 1 hour. The reaction was concentrated and the residue partitioned between DCM (5 mL) and an aqueous solution of HCl (5 mL, 1N). The aqueous layer was separated and extracted with DCM (3 × 10 mL). The organic extracts were combined, washed with HCl (5 mL, 1 N), brine (5 mL), dried over Na_2SO_4, filtered and concentrated *in vacuo* to give JBIR-22 ethyl ester **S3b** as a colourless oil (121 mg, 100%), which was used in the next step without further purification. To a solution of **S3b** (110 mg, 0.25 mmol, 1.0 eq.) in EtOH (3 mL) was added an aqueous solution of NaOH (2 mL, 2N) and the reaction was heated to 110 °C under

microwave irradiation for 20 minutes. The reaction was diluted with an aqueous solution of HCl (5 mL, 1N) and extracted with DCM (3 × 5 mL). The organic layers were combined, washed with brine (10 mL), dried over Na_2SO_4, filtered, concentrated *in vacuo* and purified *via* the Biotage SP4 (Reverse-phase silica-packed SNAP column 4 g; 20-100% H_2O/(MeOH:MeCN)) to give the title product **2b** as an orange solid (78 mg, 74%). A portion of **2b** was treated with Et_2N to form the salt as a colourless amorphous solid. Spectroscopic data was obtained for the JBIR-22 **2b**-Et_2N salt. $[\alpha]_D^{23}$= -42.0 (*c* 0.1, MeOH); **IR** (thin film) ν $_{max}$: 2916, 2849, 1647, 1560, 1437, 1387, 1234, 1065, 1032, 997, 891, 781; **^{1}H NMR** (500 MHz, Acetone-d_6) δ 5.52 (ddd, *J* = 9.8, 4.5, 2.5 Hz, 1H, C4-H), 5.30 (d, *J* = 9.8 Hz, 1H, C5-H), 3.92 (dd, *J* = 11.5, 5.8 Hz, 1H, C2-H), 3.36 (dd, *J* = 10.0, 1.2 Hz, 1H, C5'-H), 2.72 (s, 3H, C10'-H_3), 2.65 – 2.55 (m, 1H, C3-H), 2.31 (dd, *J* = 13.7, 1.2 Hz, 1H, C6'-H), 2.02 – 1.97 (m, 2H, C8'-H, C10-H_2), 1.74 – 1.70 (m, 1H, C7-H_2), 1.70 – 1.68 (m, 3H, C6-H, C8-H_2, C9-H_2), 1.64 (dd, *J* = 13.7, 10.0 Hz, 1H, C6'-H_2), 1.51 – 1.40 (m, 1H, C11-H), 1.29 – 1.27 (m, 2H, C8-H_2, C9-H_2), 1.09 – 0.98 (m, 1H, C7-H_2), 0.91 (d, *J* = 6.6 Hz, 3H, C9'-H_3), 0.91 (d, *J* = 6.6 Hz, 3H, C12'-H_3), 0.75 (d, *J* = 7.3 Hz, 3H, C12-H_3), 0.73 – 0.68 (m, 1H, C10-H_2); **^{13}C NMR** (126 MHz, Acetone-d_6) δ 196.4 (C1), 195.7 (C4'), 180.1 (C11'), 174.8 (C2'), 133.4 (C4), 131.2 (C5), 101.5 (C3'), 79.9 (C7'), 63.2 (C5'), 51.0 (C2), 43.3 (C6), 37.3 (C11), 37.1 (C6'), 36.6 (C8'), 34.4 (C7), 32.3 (C3), 31.0 (C10), 27.7 (C9, C8), 26.8 (C10'), 18.6 (C9'), 18.3 (C12), 16.8 (C12'); ***m/z*** (ES^-) 418.22 ($[M-H]^-$, 100 %); **HRMS** (ES^-) Calcd for $C_{23}H_{32}O_6N$ $[M-H]^-$: 418.2235, found 418.2226.

Diethyl (2-(2,2-dimethyl-4,6-dioxo-1,3-dioxan-5-ylidene)-2-hydroxyethyl)phosphonate (S4)

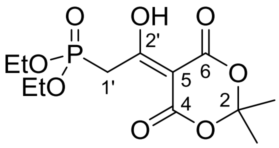

Thionyl chloride (1.11 mL, 15.3 mmol, 3.0 eq.) was added dropwise to diethylphosphoacetic acid (1.0 g, 5.1 mmol, 1.0 eq.) and the reaction was stirred at room temperature for 3 hours. The resulting red solution (diethylphosphoacetyl chloride) was added dropwise to a solution of 2,2-dimethyl-1,3-dioxane-4,6-dione (0.59 g, 4.08 mmol, 0.8 eq.) and pyridine (0.82 mL, 10.2 mmol, 2 eq.) in DCM (25 mL) at 0 °C. The reaction was stirred for 1 hour at 0 °C followed by 1 hour at room temperature. The reaction was washed with an aqueous solution of HCl (3 × 10 mL), brine (10 mL), dried over Na_2SO_4, filtered and concentrated *in vacuo* to yield the title product **S4** as a red oil which solidified at ~4 °C to yield a red amorphous solid (1.26 g, 96%). **S4** was used in the next step without further purification. **IR** (KBr) ν $_{max}$: 2995 (OH), 2928 (OH), 1736 (C=O), 1668 (C=O), 1578, 1396, 1248, 1196, 1146, 1013, 920; **^{1}H NMR** (500 MHz, Chloroform-*d*) δ 4.18 (dq, *J* = 8.1, 7.1 Hz, 4H, OCH_2CH_3), 3.93 (d, *J* = 23.9 Hz, 2H, C1'-H_2), 1.76 (s, 6H, $(CH_3)_2$), 1.33 (td, *J* = 7.1, 0.6 Hz, 6H, OCH_2CH_3); **^{13}C NMR** (126

MHz, Chloroform-*d*) δ 187.6 (C2'), 170.3 (C6), 160.6 (C4), 105.4 (C2), 93.4 (C5), 63.2 (d, *J* = 6.2 Hz, $O\underline{C}H_2CH_3$), 34.5 (d, *J* = 126.9 Hz, C1'), 27.0 ($(\underline{C}H_3)_2$), 16.5 ($OCH_2\underline{C}H_3$); **^{31}P NMR** (202 MHz, Chloroform-*d*) δ 19.0; ***m/z*** (ES^+) 323.09 ($[M+H]^+$, 100 %); **HRMS** (ES^+) Calcd for $C_{12}H_{20}O_8P$ $[M+H]^+$: 323.0890, found 323.0893.

***S*-(*tert*-Butyl) 4-(diethoxyphosphoryl)-3-oxobutanethioate (S5)**[15]

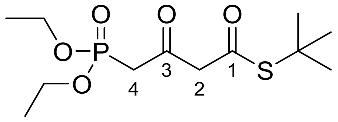

2-Methyl-2-propanethiol (4.46 mL, 39.5, 2.0 eq.) was added to a solution of **S4** (6.37 g, 19.8, 1.0 eq.) in MeCN (120 mL) and the reaction was heated at reflux for 3 hours. The reaction was cooled to room temperature, concentrated *in vacuo* and purified *via* the Biotage SP4 (silica-packed SNAP column 200 g; 0-5% MeOH/DCM) to give the title product **S5** as an orange oil (5.20 g, 85%). **^{1}H NMR** (500 MHz, Chloroform-*d*) δ 4.15 (dq, *J* = 8.1, 7.1 Hz, 4H, $OC\underline{H}_2CH_3$), 3.80 (s, 2H, C2-$\underline{H}_2$), 3.24 (d, *J* = 22.7 Hz, 2H, C4-$\underline{H}_2$), 1.47 (s, 9H, $(C\underline{H}_3)_3$), 1.34 (td, *J* = 7.1, 2.4 Hz, 6H, $OCH_2C\underline{H}_3$); **^{13}C NMR** (126 MHz, Chloroform-*d*) δ 194.3 (d, *J* = 6.4 Hz, C3), 192.4 (C1), 62.9 (d, *J* = 6.4 Hz, $O\underline{C}H_2CH_3$), 58.7 (C2), 49.4 ($\underline{C}(CH_3)_3$), 42.8 (d, *J* = 126.6 Hz, C4), 29.8 ($(\underline{C}H_3)_2$), 16.5 (d, *J* = 6.1 Hz, $OCH_2\underline{C}H_3$); ***m/z*** (ES^+) 333.09 ($[M+Na]^+$, 100 %); **HRMS** (ES^+) Calcd for $C_{12}H_{24}O_5PS$ $[M+H]^+$: 311.1077, found 311.1083. Spectroscopic data are in accordance with the literature.[15]

***S*-(*tert*-Butyl) (4*E*,10*E*,12*E*)-3-oxotetradeca-4,10,12-trienethioate (S6)**

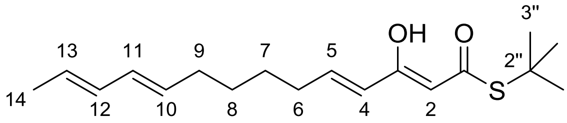

To a solution of **S5** (2.81 g, 9.05 mmol, 1.0 eq.) in THF (70 mL) at -78 °C was added LiHMDS (18.1 mL, 1 M in THF, 2.0 eq.) and the reaction was stirred for 30 minutes before the addition of a solution of **21** (1.24 g, 8.15 mmol, 0.9 eq.) in THF (35 mL). The reaction was stirred for a further 30 minutes at -78 °C before slowly warming to 0 °C over 2 hours. The reaction was quenched by the addition of a saturated aqueous solution of NH_4Cl (30 mL) and extracted with EtOAc (3 × 30 mL). The organic extracts were combined, washed with brine (30 mL), dried over $MgSO_4$, filtered and concentrated *in vacuo* and purified *via* the Biotage SP4 (silica-packed SNAP column 80 g; 0-10% EtOAc/hexanes) to give the title product **S6** as a colourless oil (1.82 g, 72%). In $CDCl_3$ at room temperature the title compound exists as a (2 : 3) *keto* : *enol* mixture. **IR** (thin film) ν_{max}: 2924, 2859, 1653 (C=O), 1582, 1074; **^{1}H NMR** (500 MHz, Chloroform-*d*) δ(*major enol-form*) 12.61 (br s, O$\underline{H}$), 6.76 – 6.54 (m, 1H, C5-$\underline{H}$), 6.05 – 5.90 (m, 2H, C11-$\underline{H}$, C12-$\underline{H}$), 5.74 – 5.61 (m, 1H, C4-$\underline{H}$), 5.61 – 5.43 (m, 2H, C10-$\underline{H}$, C13-$\underline{H}$),

5.30 (s, 1H, C2-$\underline{H}$), 2.21 – 2.14 (m, 2H, C6-$\underline{H}_2$), 2.10 – 2.01 (m, 2H, C9-$\underline{H}_2$), 1.76 – 1.68 (m, 3H, C14-$\underline{H}_3$), 1.51 (s, 9H, (C3''-$\underline{H}_3$)$_3$), 1.46 – 1.36 (m, 4H, C7-$\underline{H}_2$, C8-$\underline{H}_2$); **^{13}C NMR** (126 MHz, Chloroform-*d*) δ(*major enol-form*) 196.5 (C1), 166.8 (C3), 142.6 (C5), 131.6 (C10, C12), 130.7 (C11), 127.2 (C13), 124.4 (C4), 100.5 (C2), 48.42 (C2''), 32.7 (C6), 32.4 (C9), 30.3 ((C3'')$_3$), 29.0 ($\underline{C}H_2$), 28.1 ($\underline{C}H_2$), 18.2 (C14); **^{1}H NMR** (500 MHz, Chloroform-*d*) δ(*minor keto-form*) 6.98 – 6.79 (m, 1H, C5-$\underline{H}$), 6.21 – 6.08 (m, 1H, C4-$\underline{H}$), 6.07 – 5.93 (m, 2H, C11-$\underline{H}$, C12-$\underline{H}$), 5.63 – 5.46 (m, 2H, C10-$\underline{H}$, C13-$\underline{H}$), 3.70 (d, *J* = 1.0 Hz, 2H, C2-$\underline{H}_2$), 2.29 – 2.20 (m, 2H, C6-$\underline{H}_2$), 2.10 – 2.01 (m, 2H, C9-$\underline{H}_2$), 1.76 – 1.68 (m, 3H, C14-$\underline{H}_3$), 1.46 (s, 9H, (C3''-$\underline{H}_3$)$_3$), 1.47 – 1.39 (m, 4H, C7-$\underline{H}_2$, C8-$\underline{H}_2$); **^{13}C NMR** (126 MHz, Chloroform-*d*) δ(*minor keto-form*) 192.9 (C1), 192.0 (C3), 150.6 (C5), 131.7 (C10), 131.4 (C12), 130.9 (C11), 129.8 (C4), 127.3 (C13), 56.3 (C2), 49.1 (C2''), 32.6 (C6), 32.4 (C9), 29.8 ((C3''-$\underline{H}_3$)$_3$), 29.0 ($\underline{C}H_2$), 28.1 ($\underline{C}H_2$), 18.2 (C14); ***m/z*** (ES$^+$) 309.19 ([M+H]$^+$, 100 %); **HRMS** (ES$^+$) Calcd for $C_{18}H_{29}O_2S$ [M+H]$^+$: 309.1883, found 309.1883.

7 NMR spectra of novel compounds

13, $CDCl_3$, 500 MHz

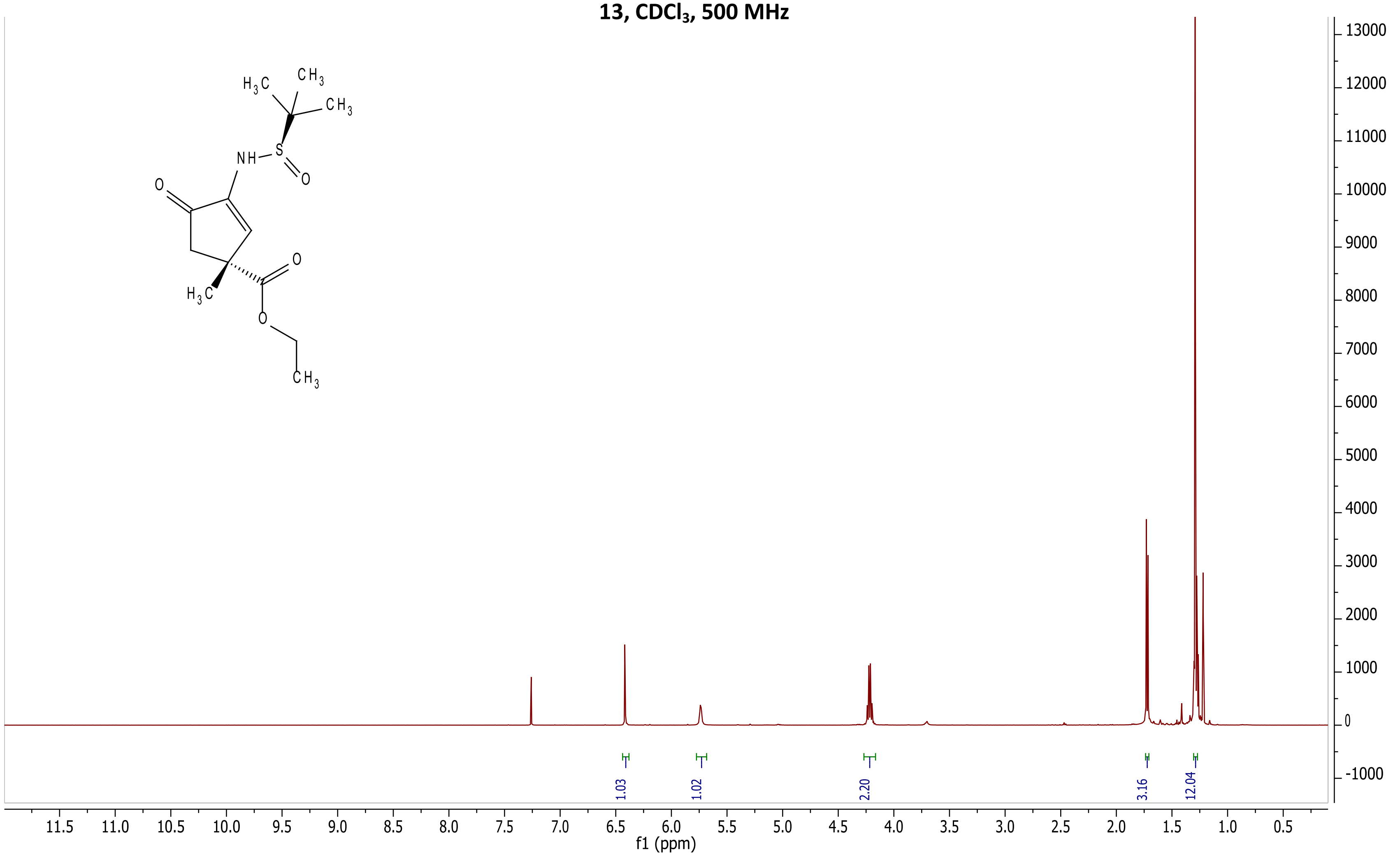

8

13, $CDCl_3$, 125 MHz

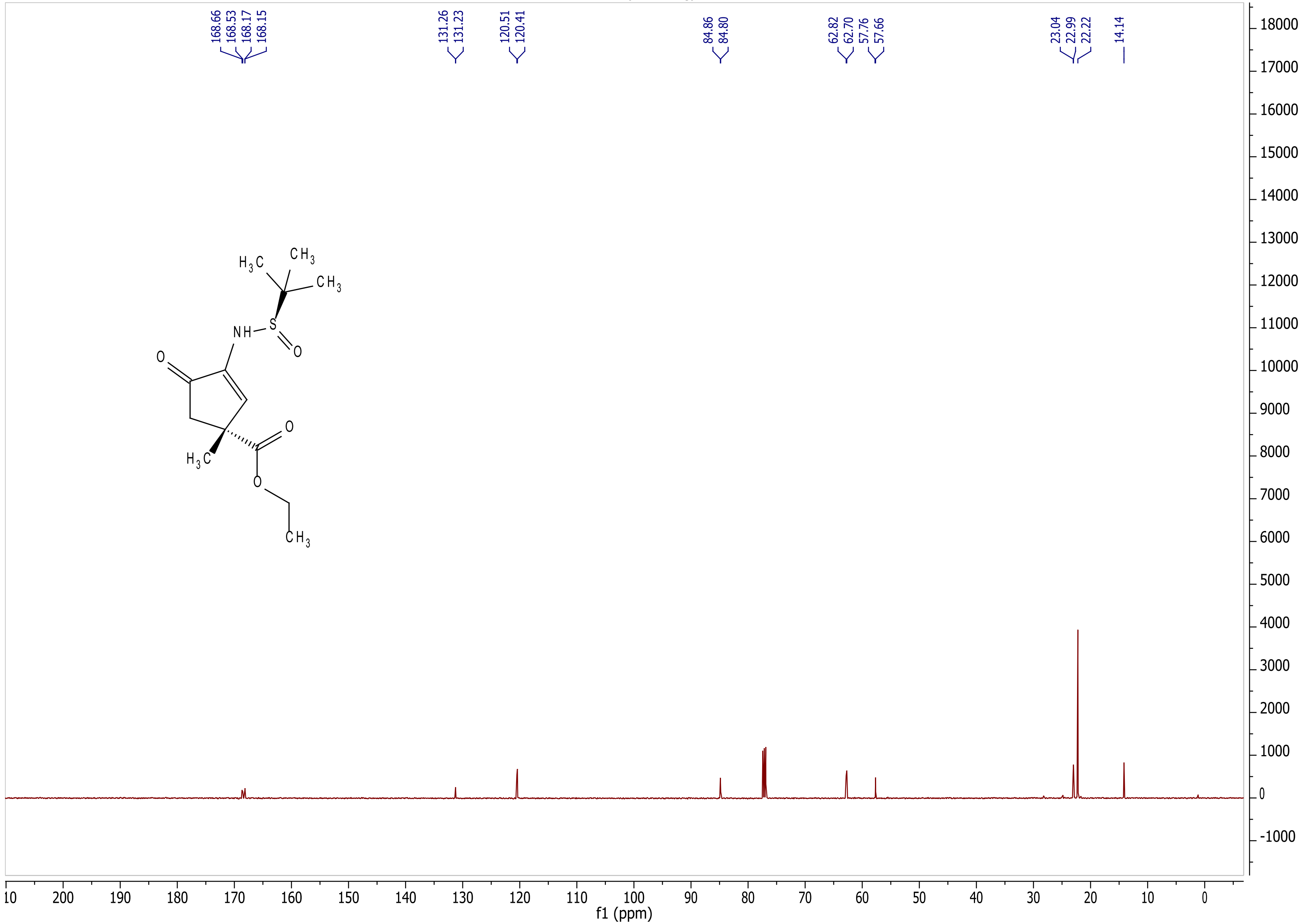

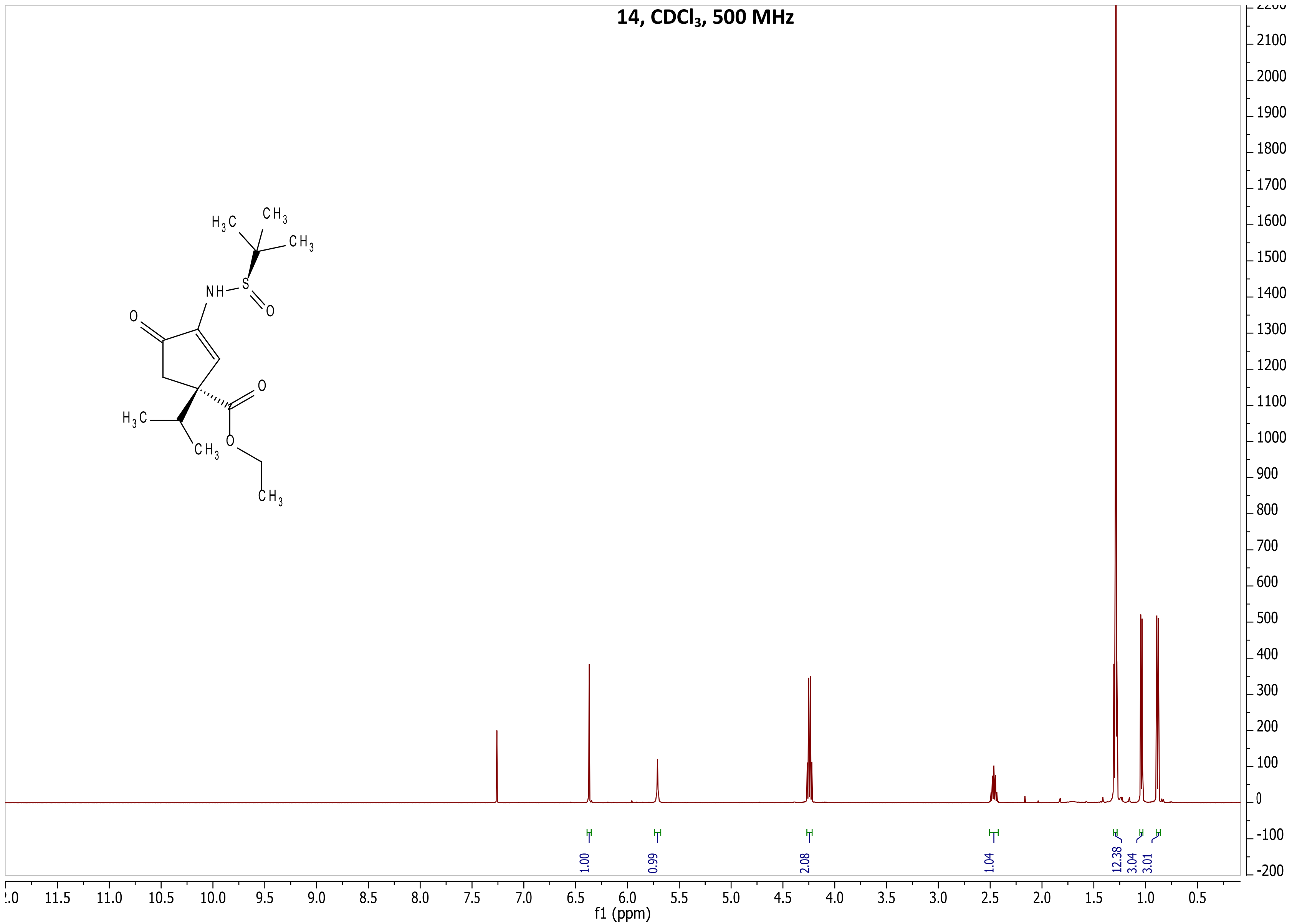

14, CDCl3, 500 MHz
H3C
CH3
CH3
NH
S
O
O
O
H3C
CH3
O
CH3
1.00
0.99
2.08
1.04
12.38
3.04
3.01
f1 (ppm)

14, $CDCl_3$, 125 MHz

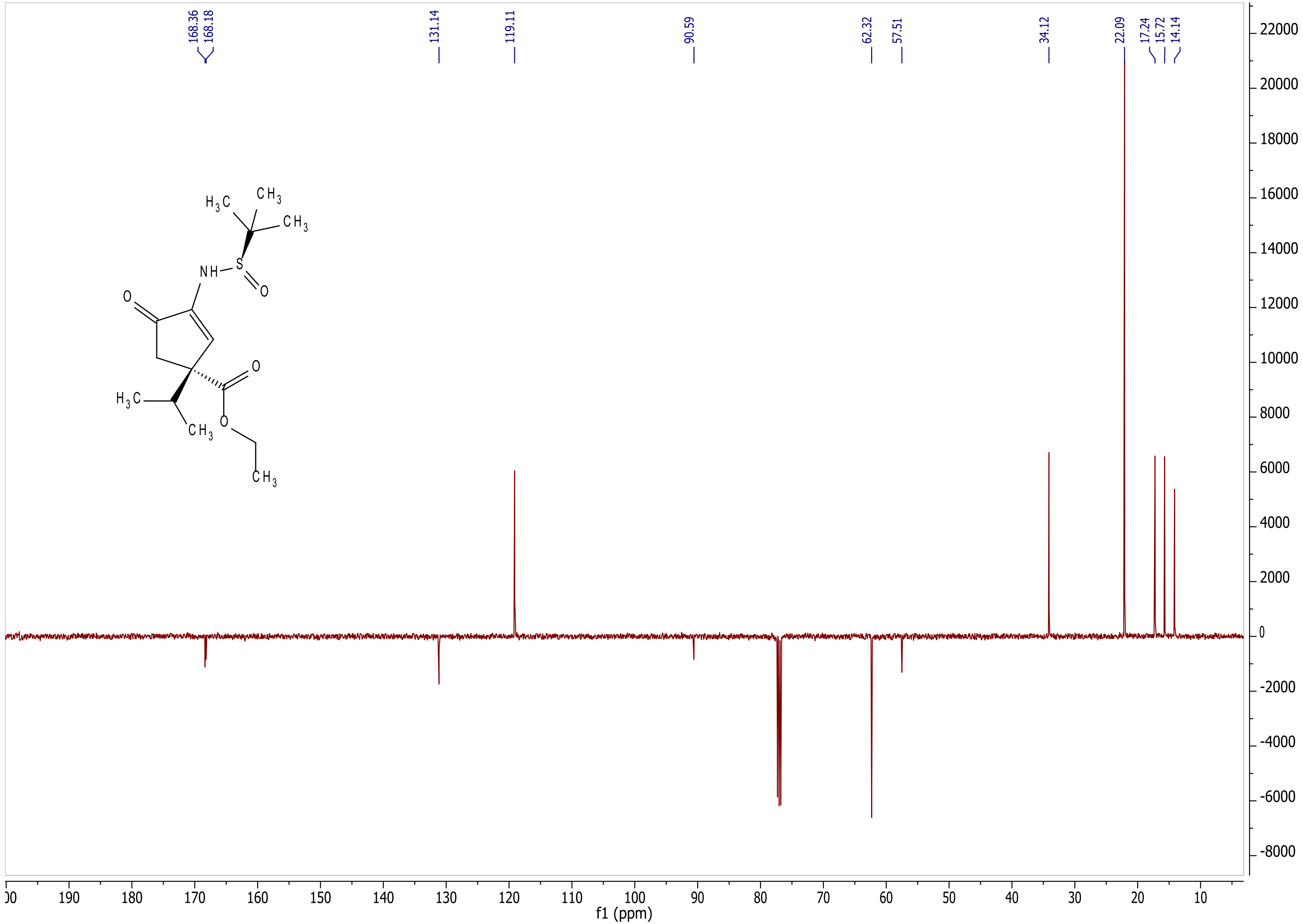

15, $CDCl_3$, 500 MHz

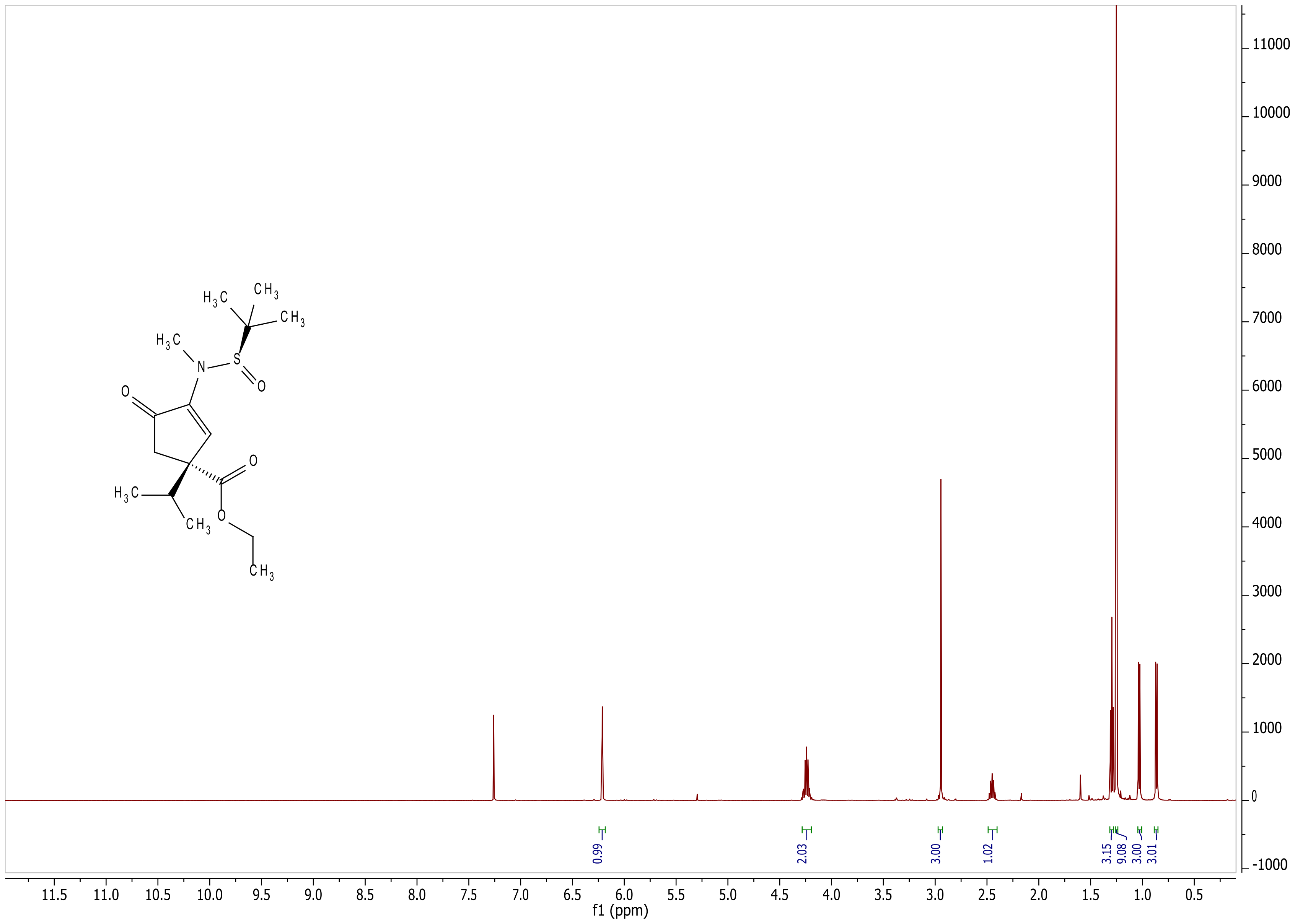

15, $CDCl_3$, 125 MHz

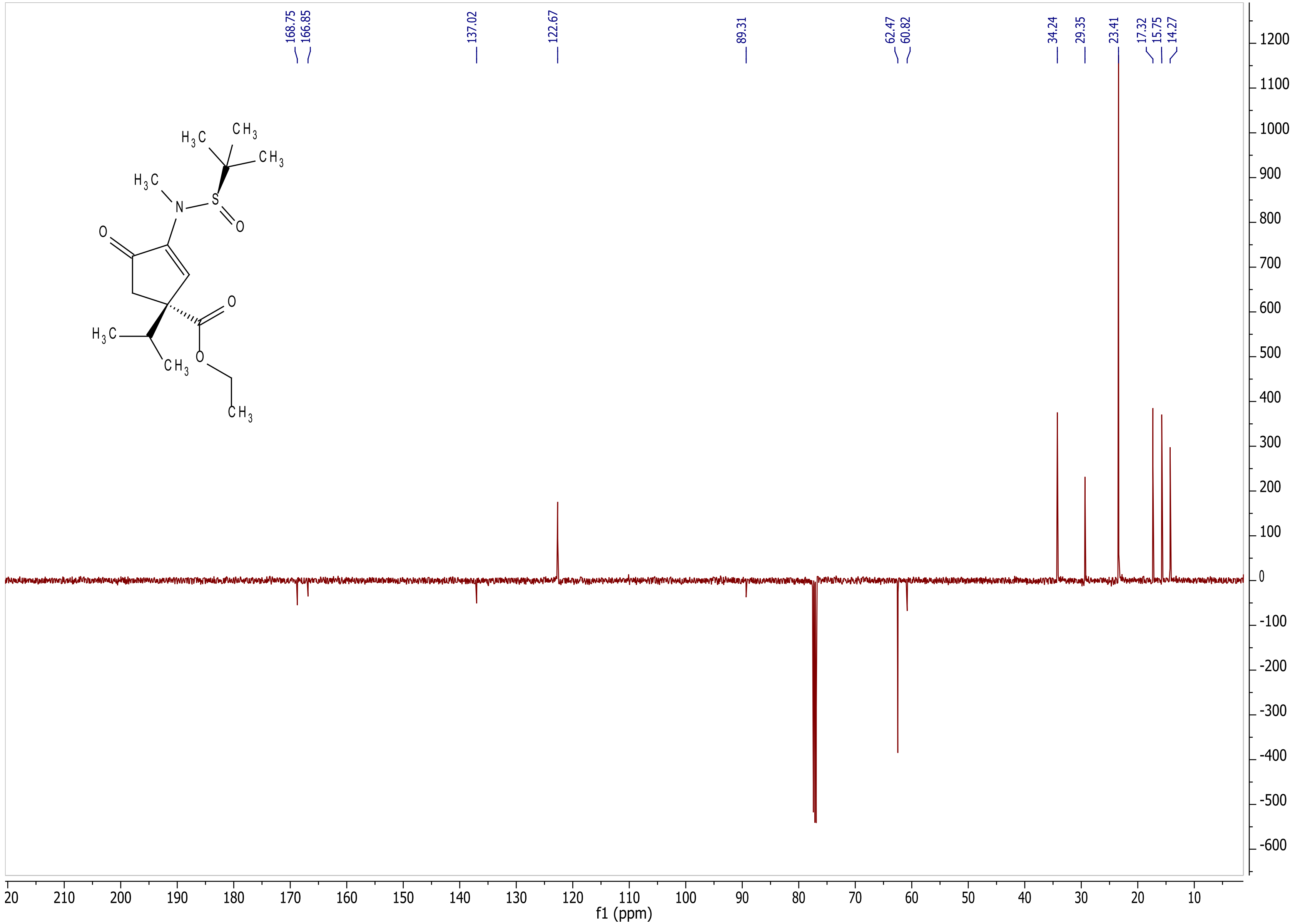

12, $CDCl_3$, 500 MHz

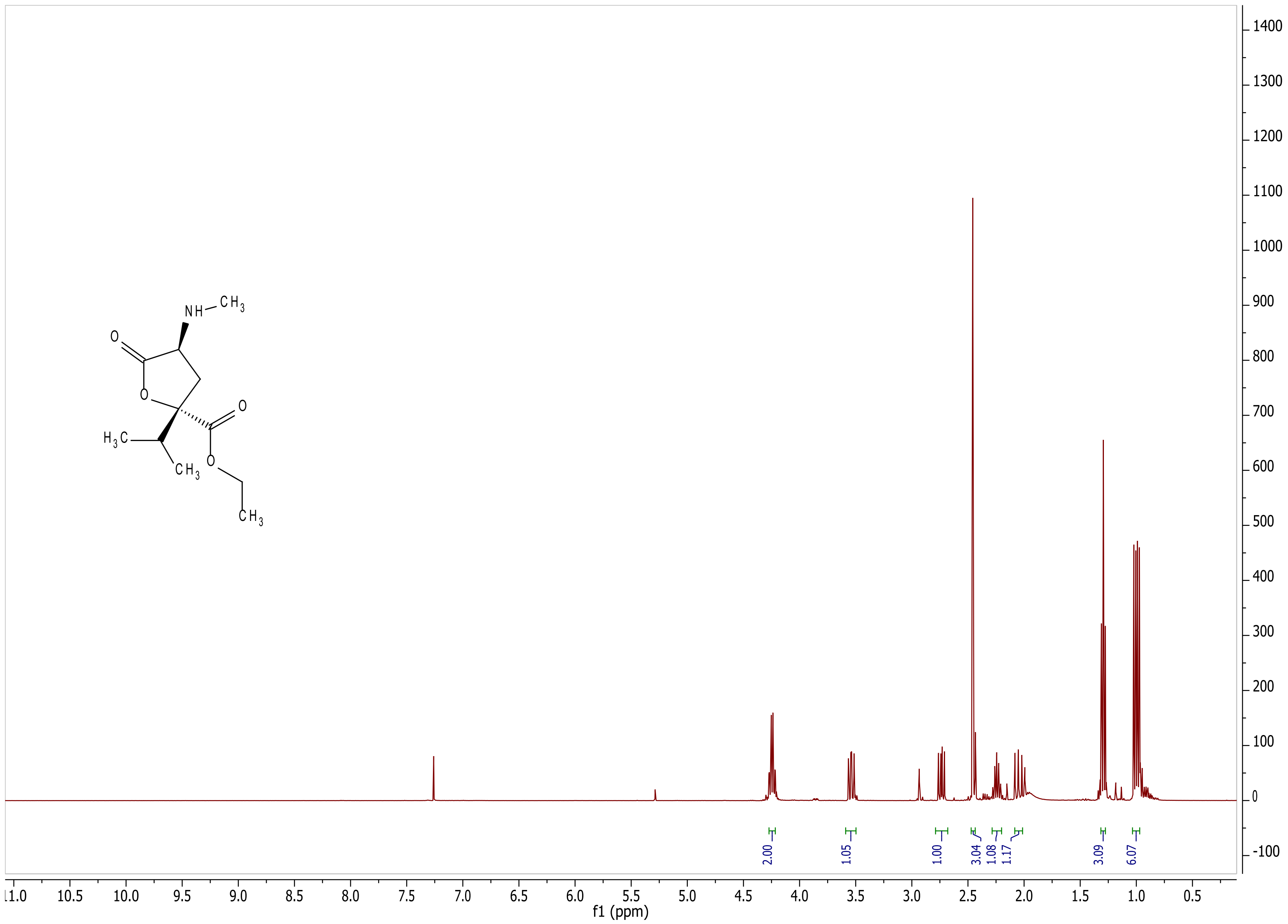

12, $CDCl_3$, 125 MHz

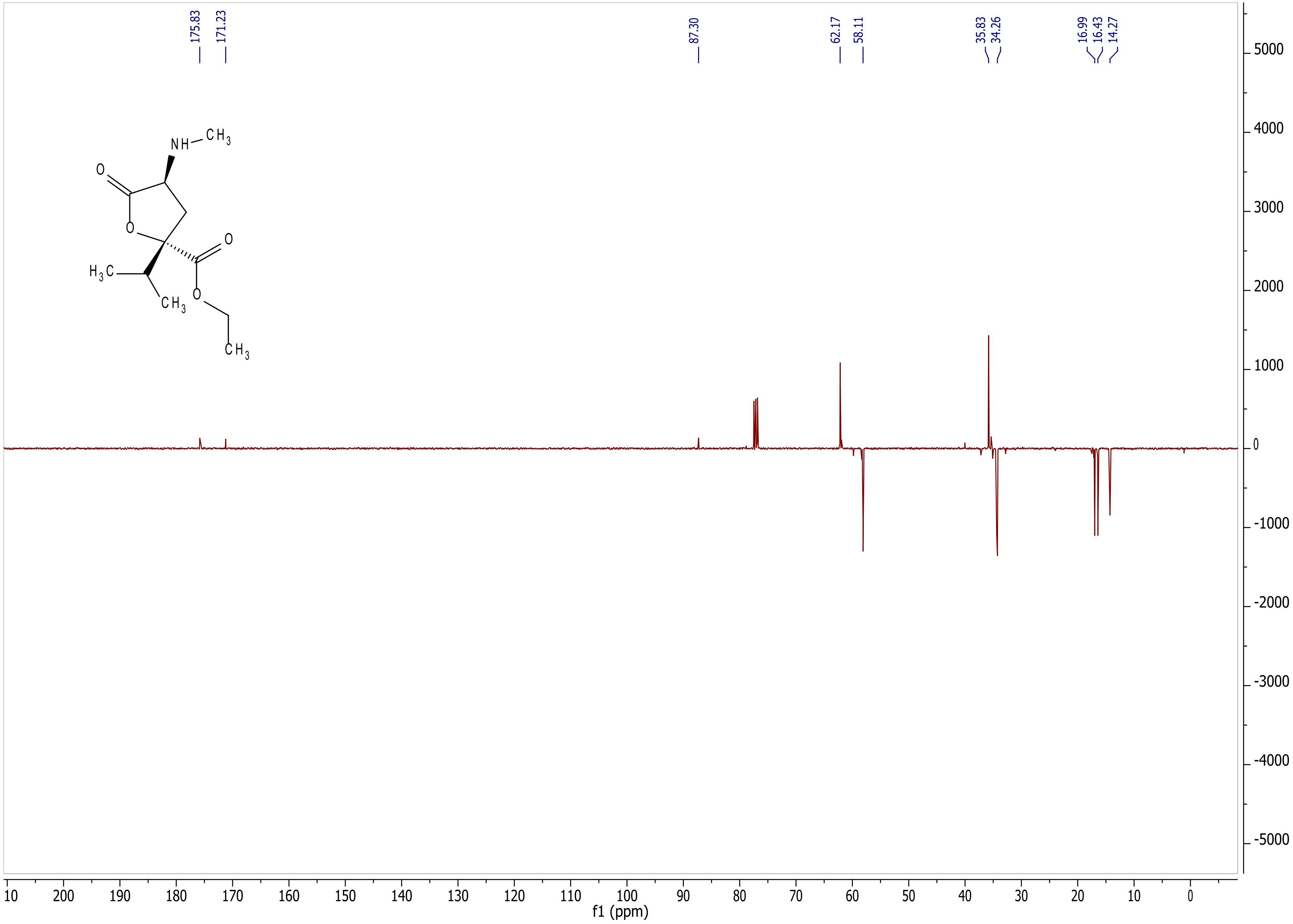

20, $CDCl_3$, 500 MHz

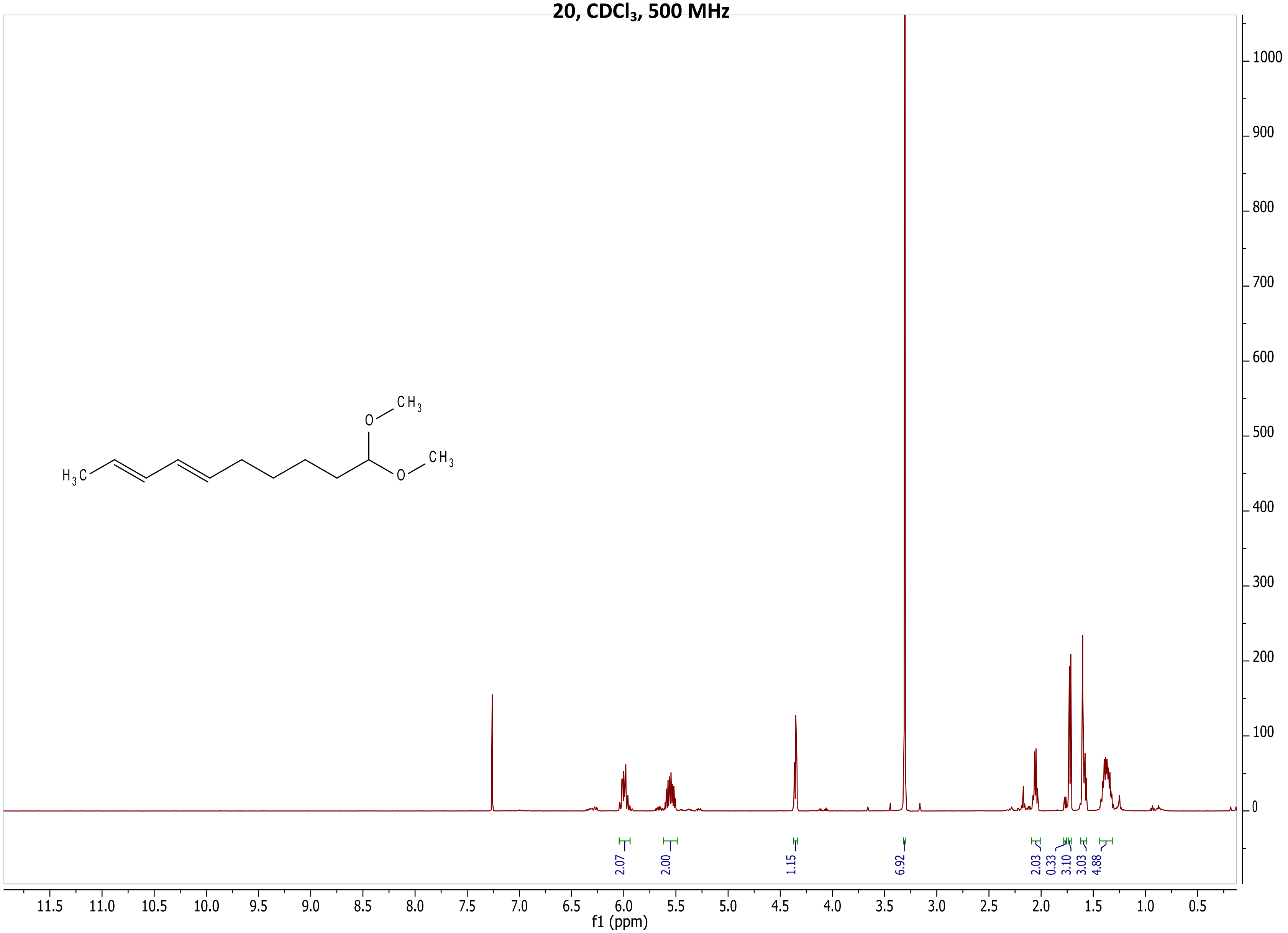

20, $CDCl_3$, 125 MHz

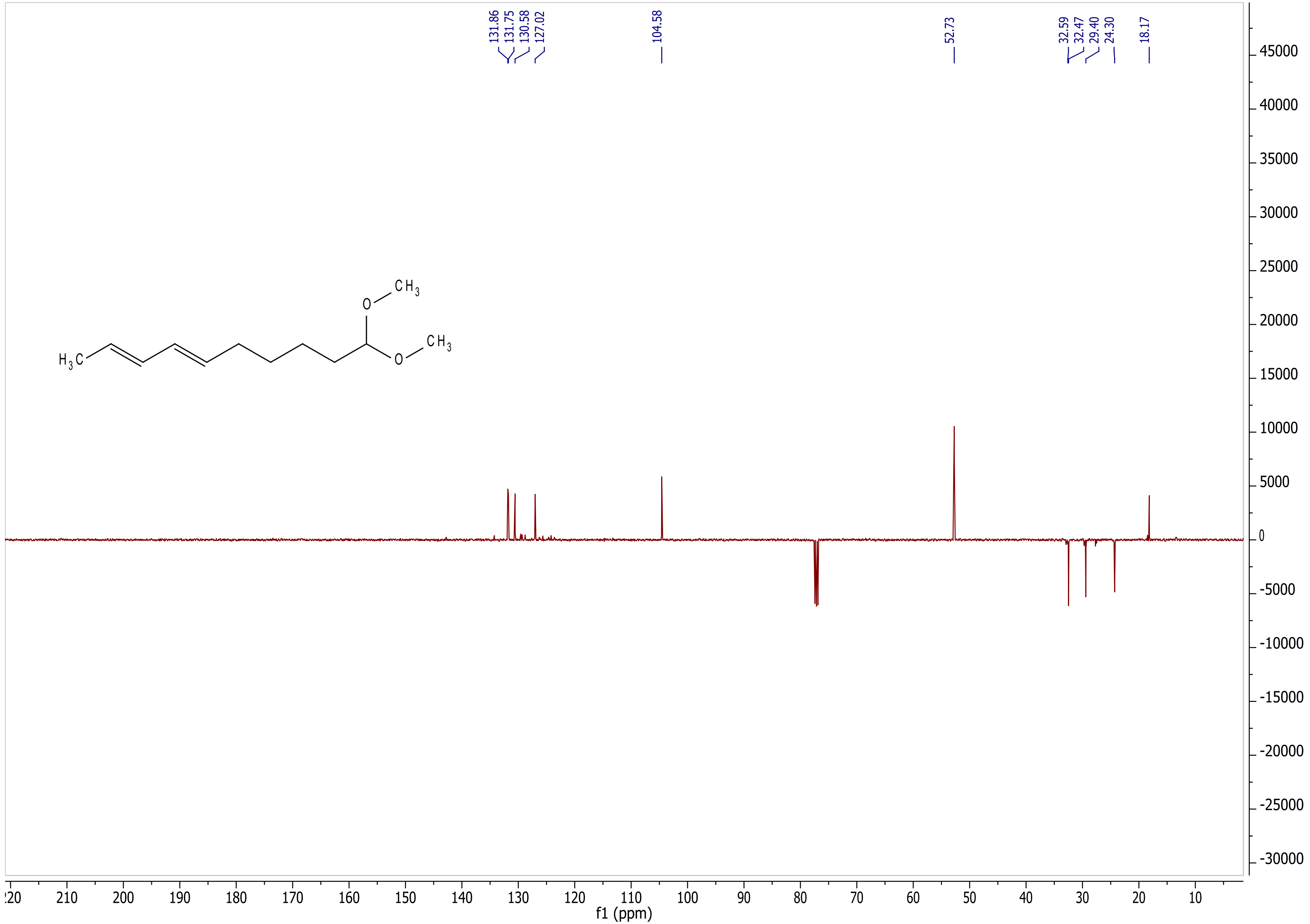

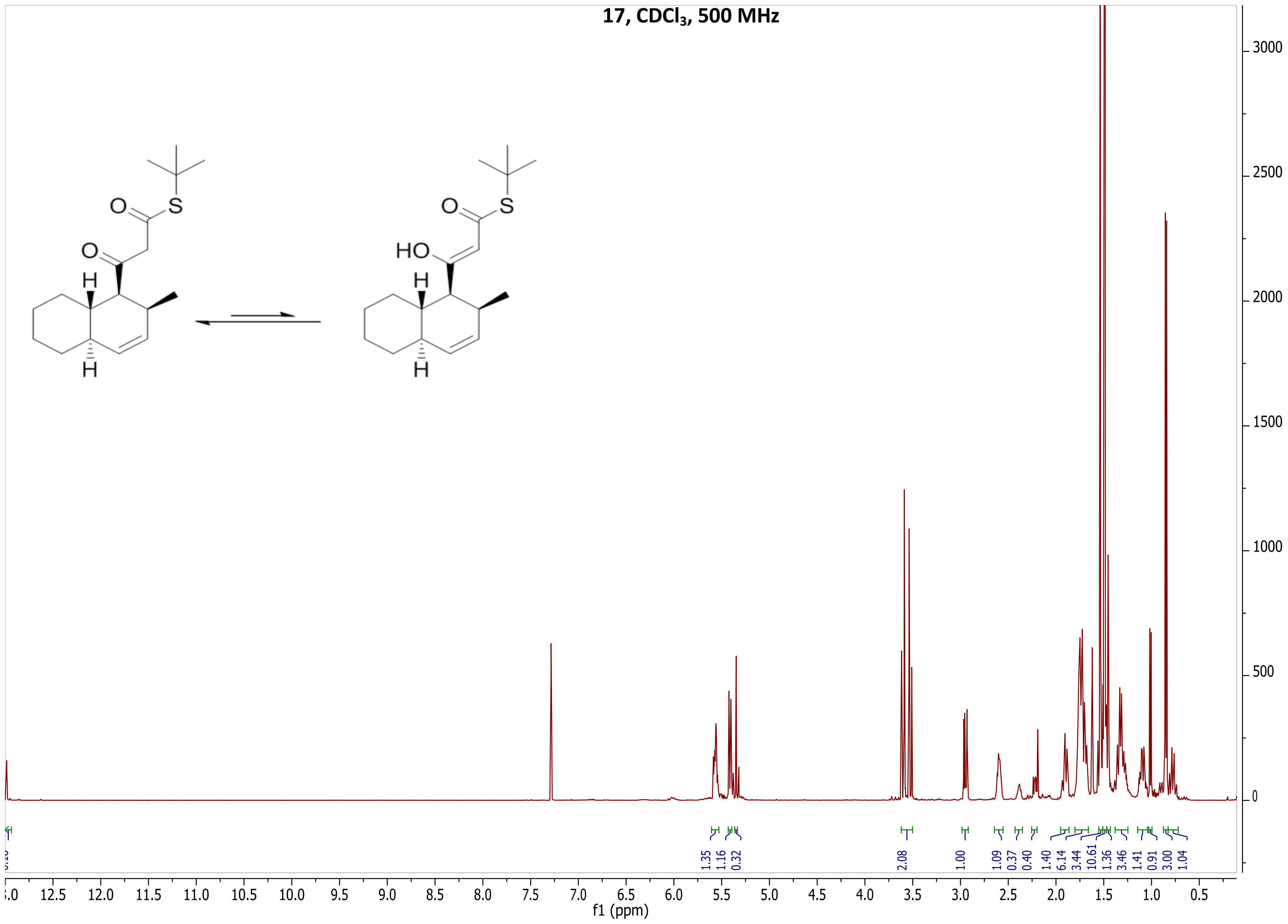

17, CDCl3, 500 MHz
O
S
O
HO
H
H
f1 (ppm)

17, CDCl$_3$, 125 MHz

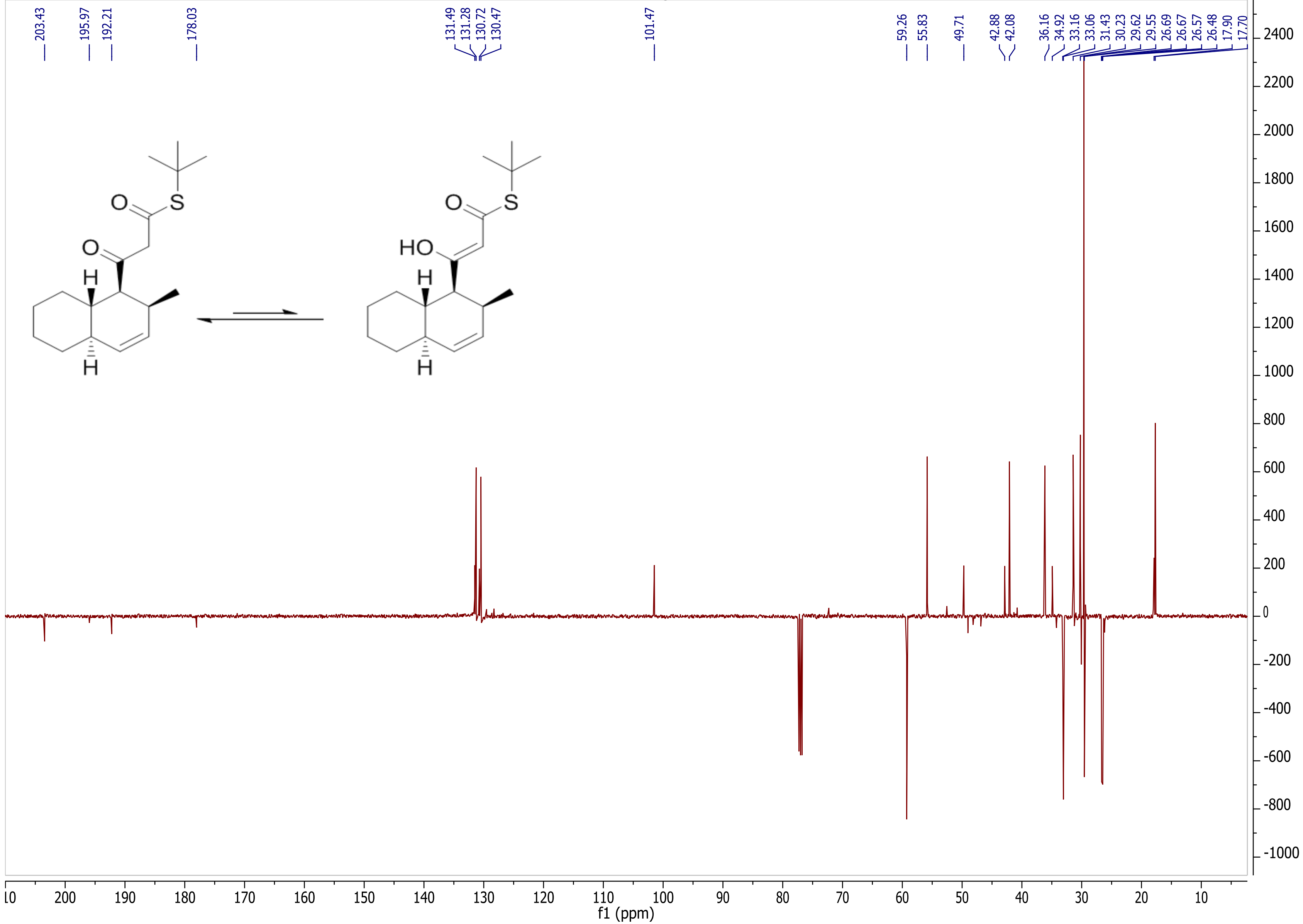

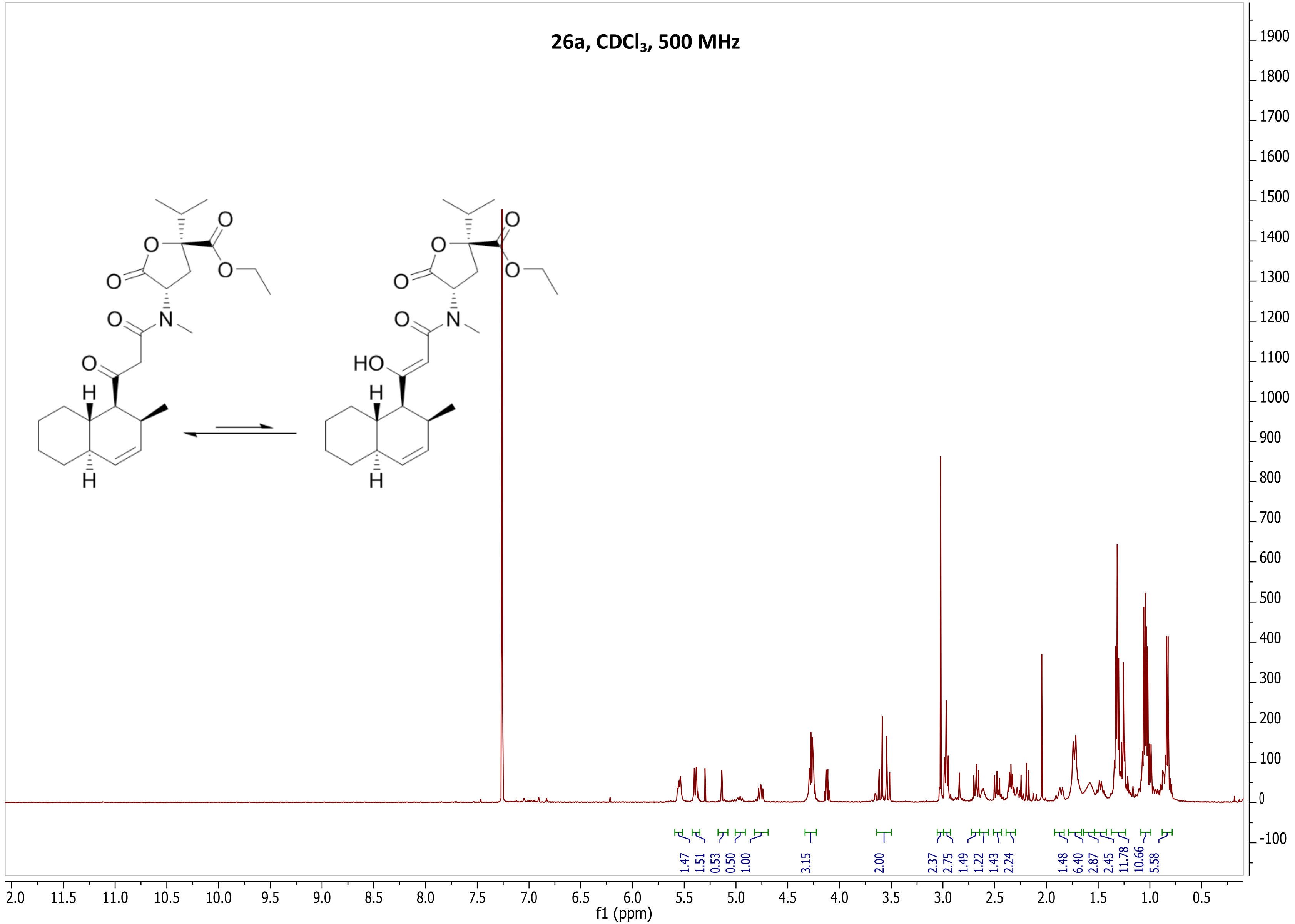

26a, CDCl3, 500 MHz
f1 (ppm)

26a, $CDCl_3$, 125 MHz

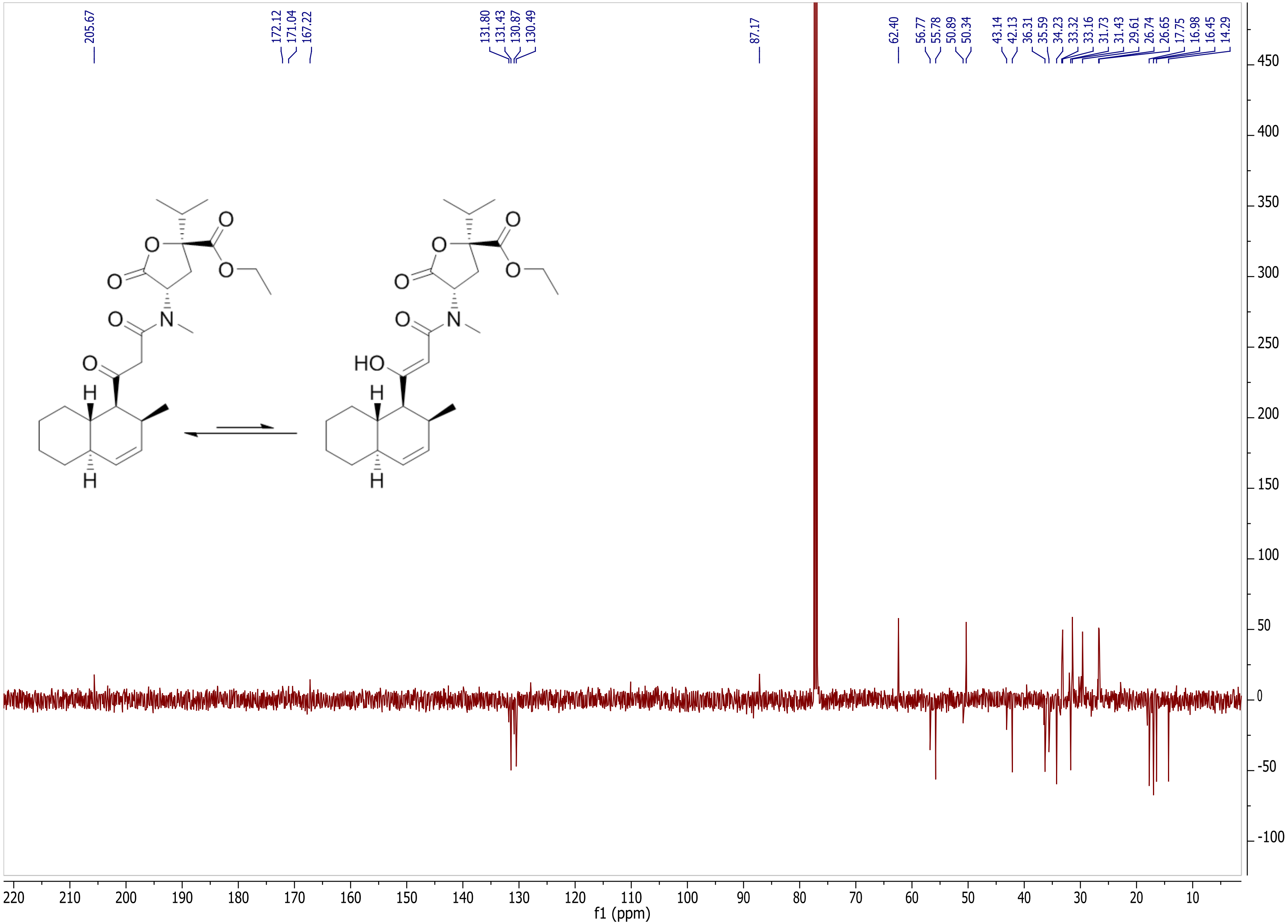

26b, $CDCl_3$, 500 MHz

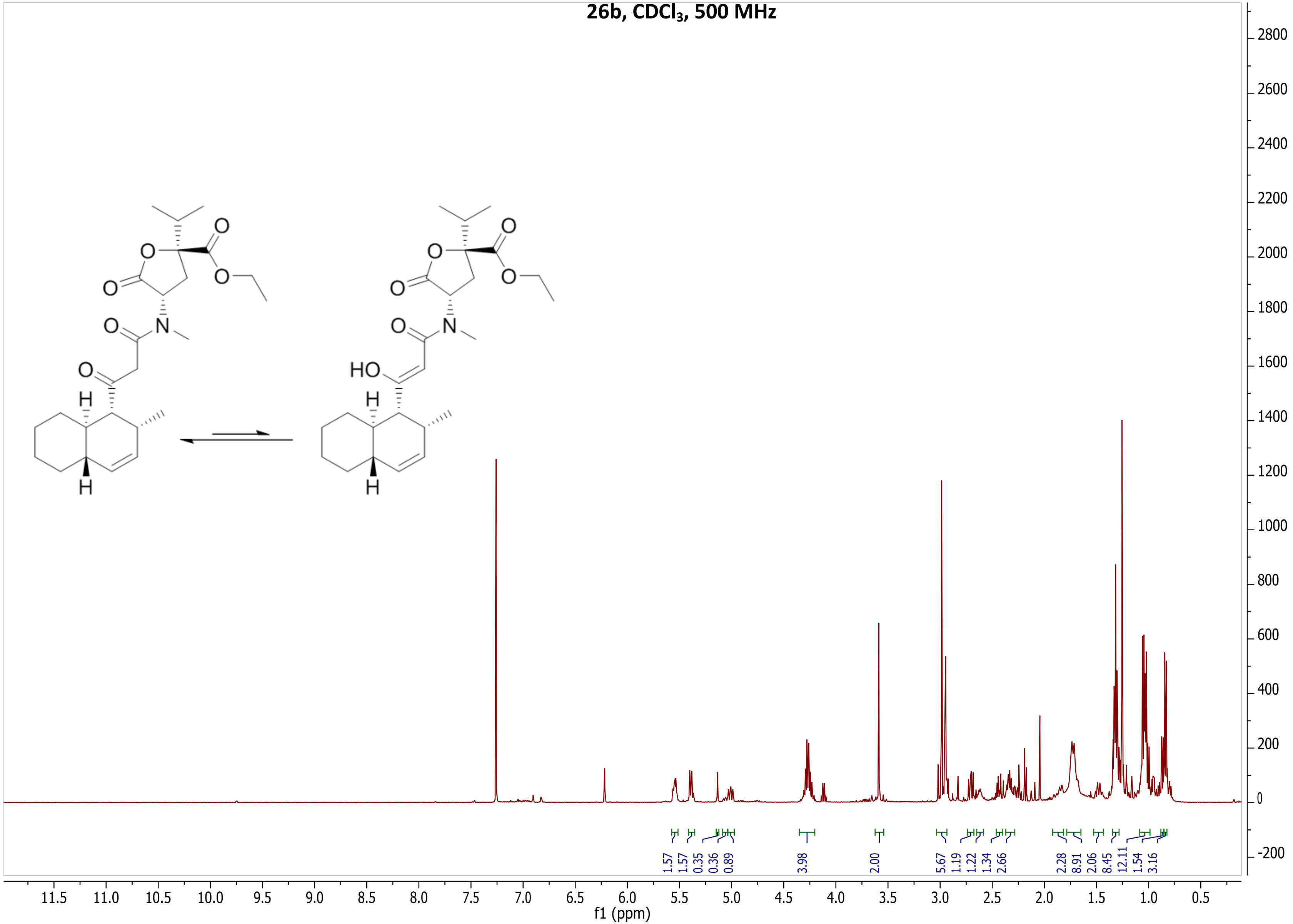

26b, CDCl$_3$, 125 MHz

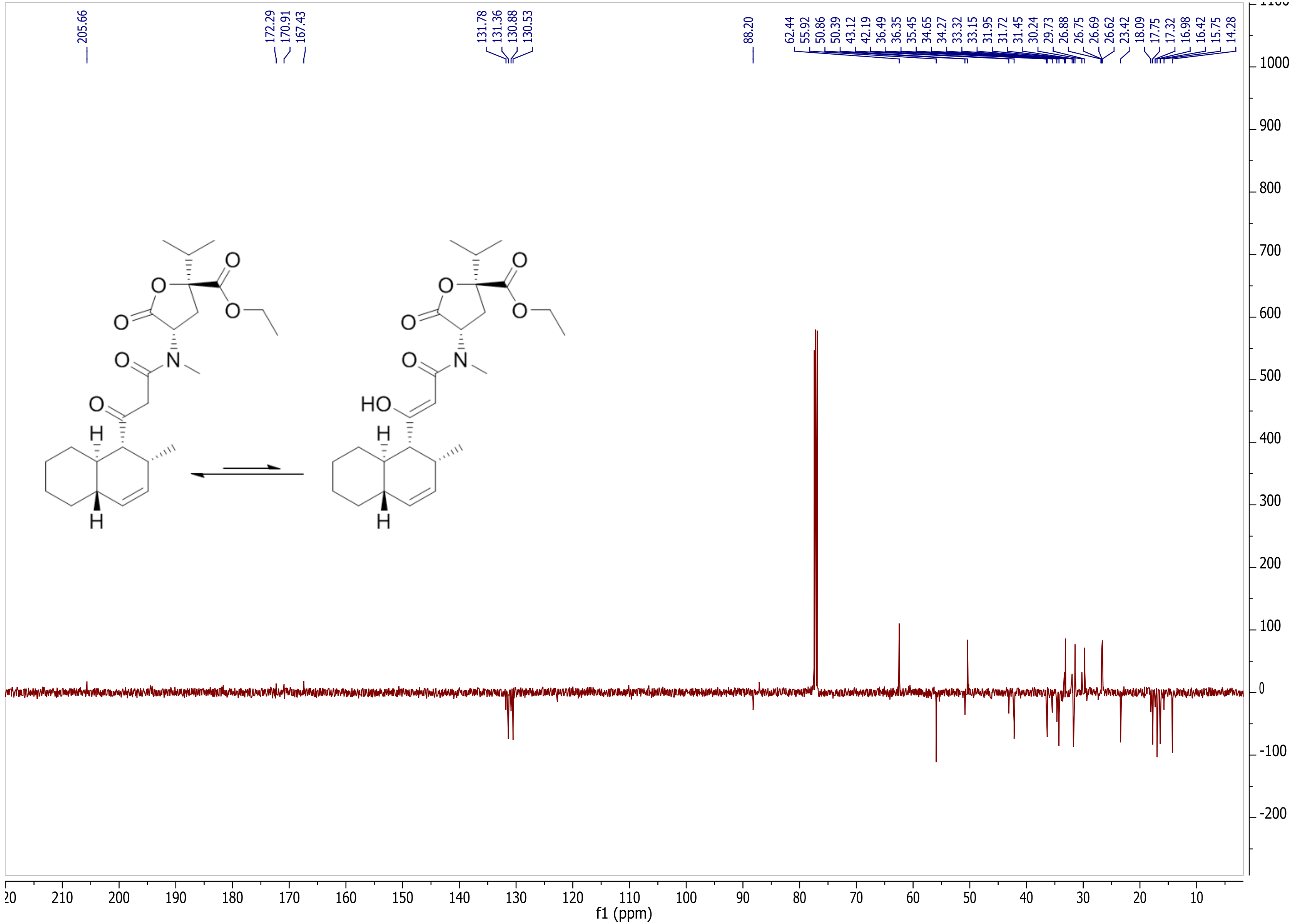

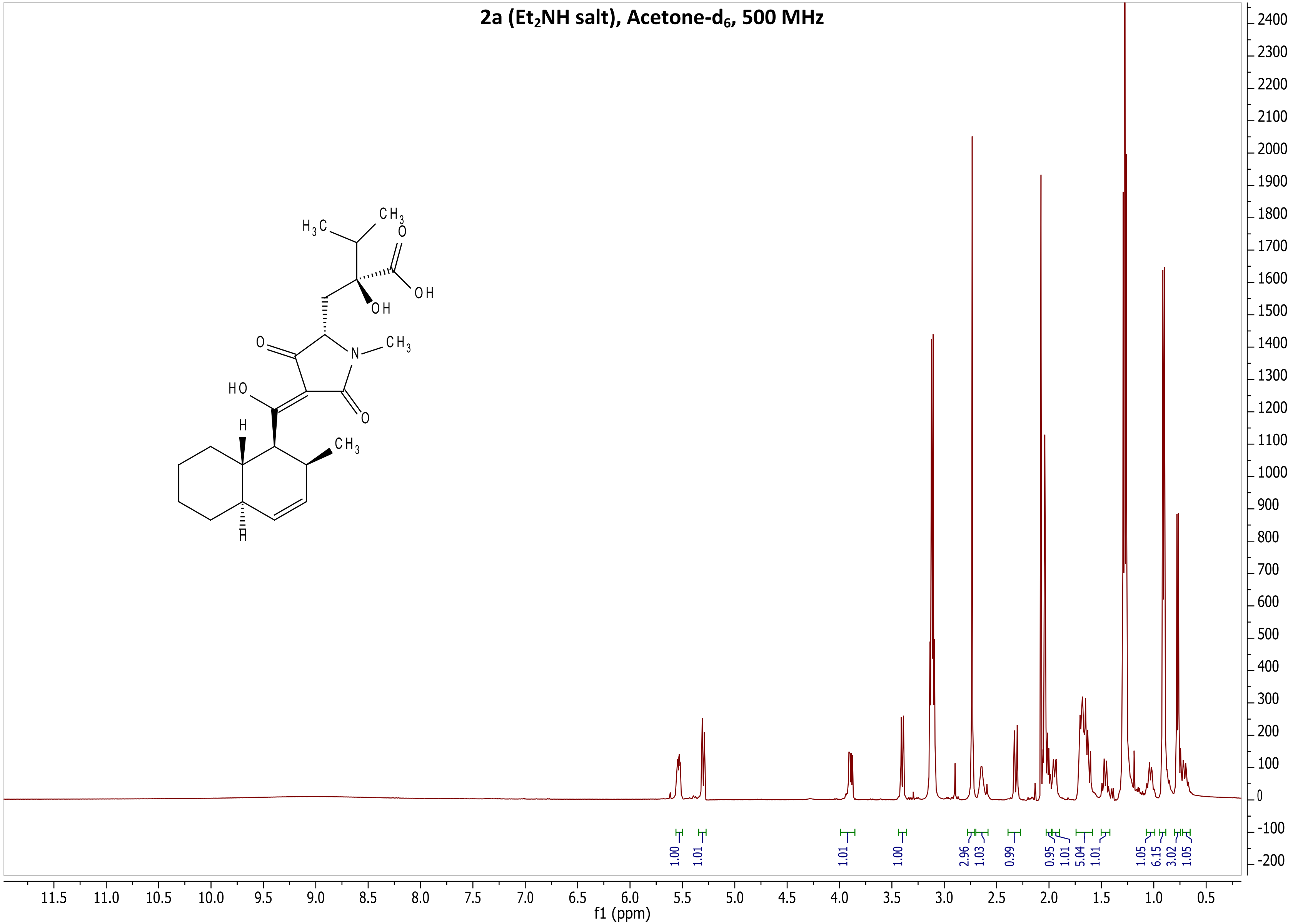
2a (Et2NH salt), Acetone-d6, 500 MHz
f1 (ppm)

2a (Et_2NH salt), Acetone-d_6, 125 MHz

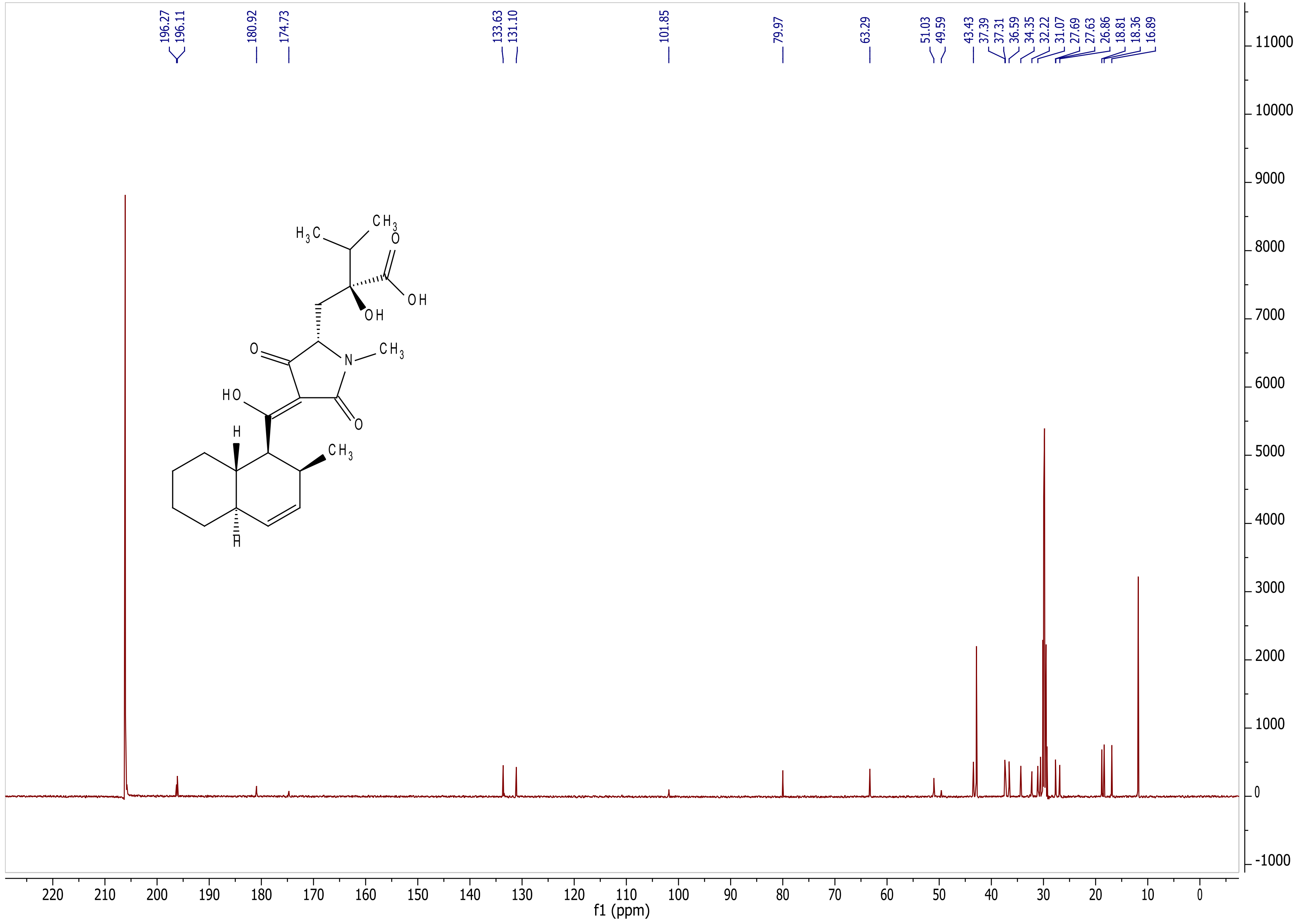

2b (Et_2NH salt), Acetone-d_6, 500 MHz

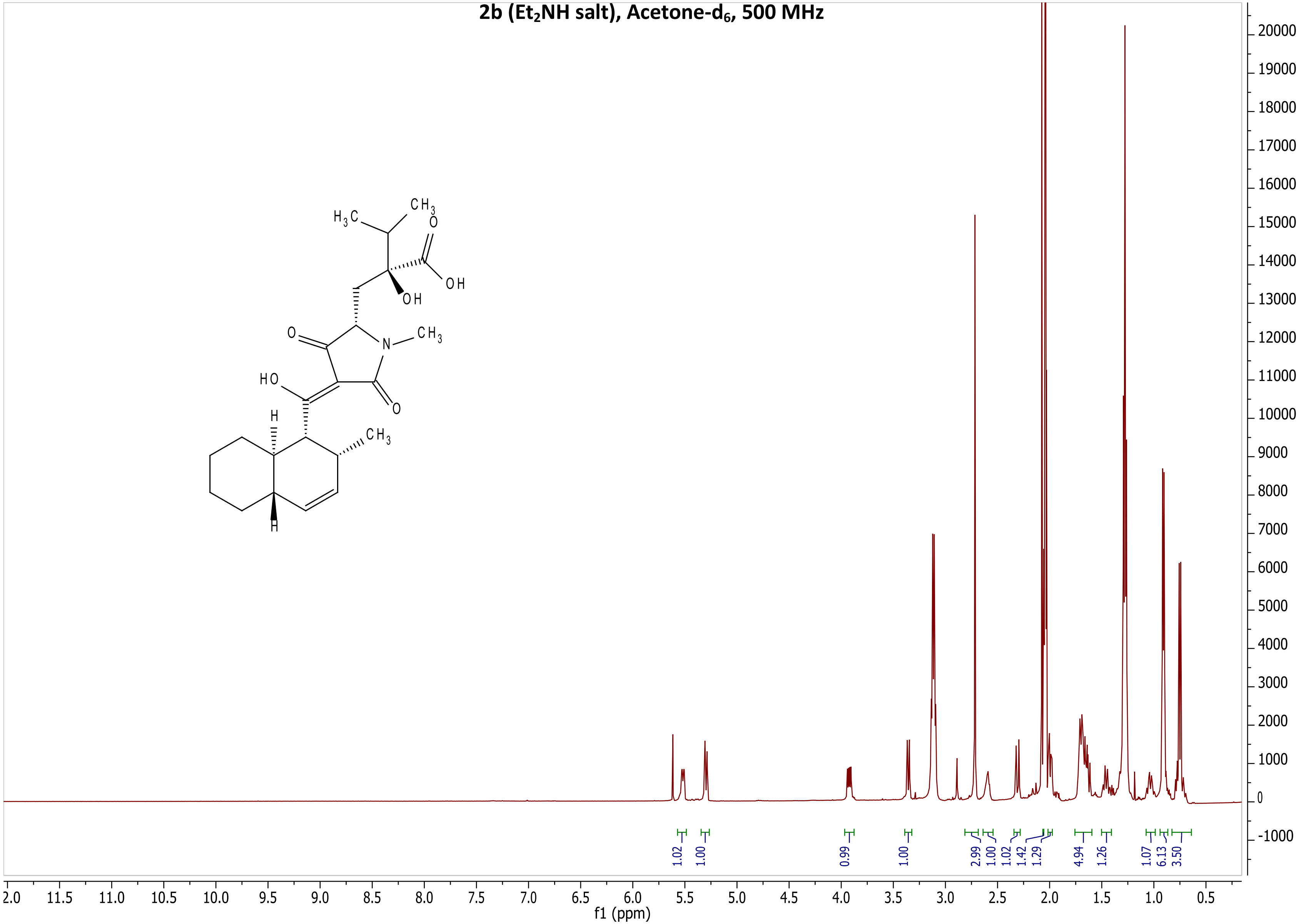

2b (Et_2NH salt), Acetone-d_6, 125 MHz

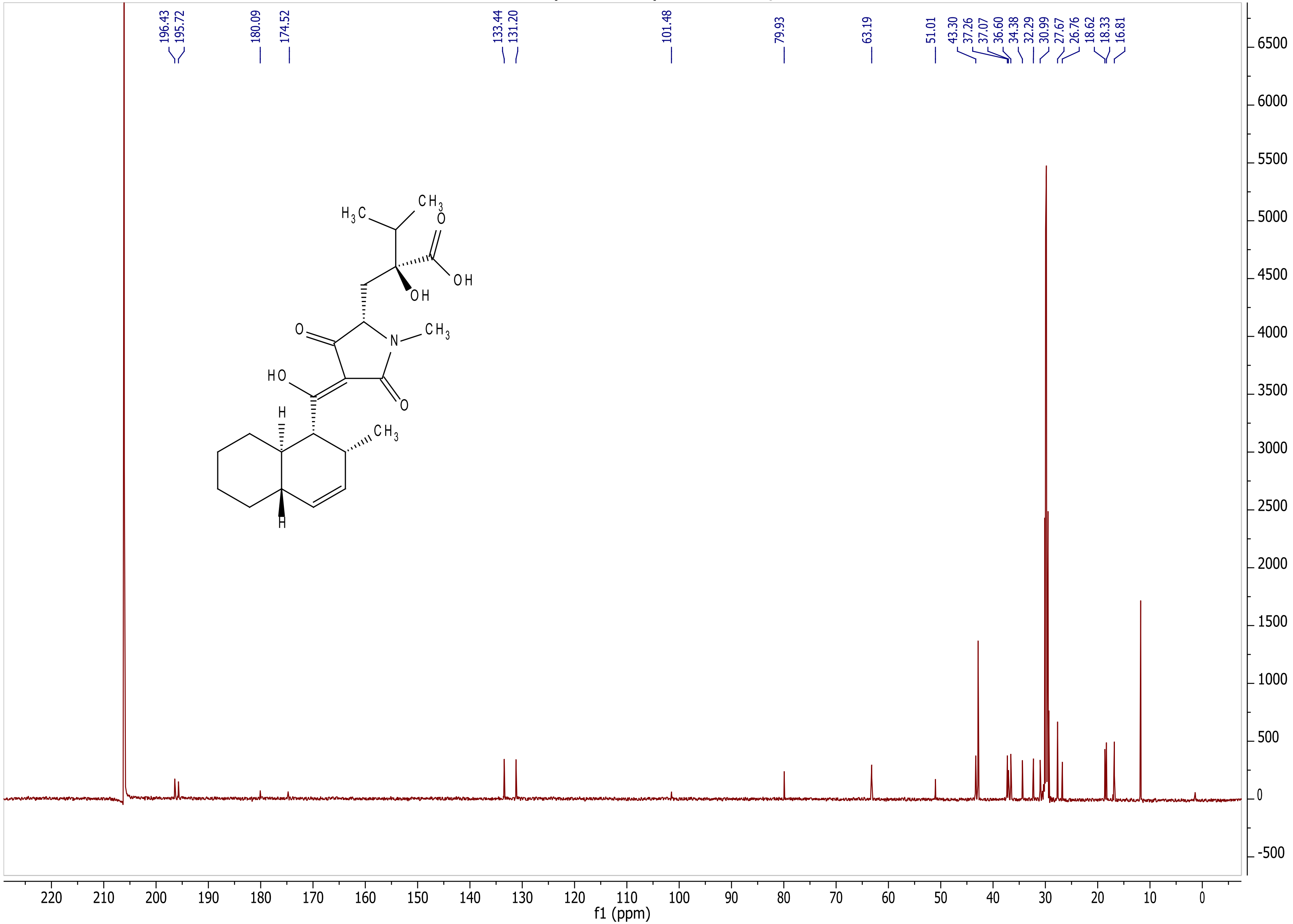

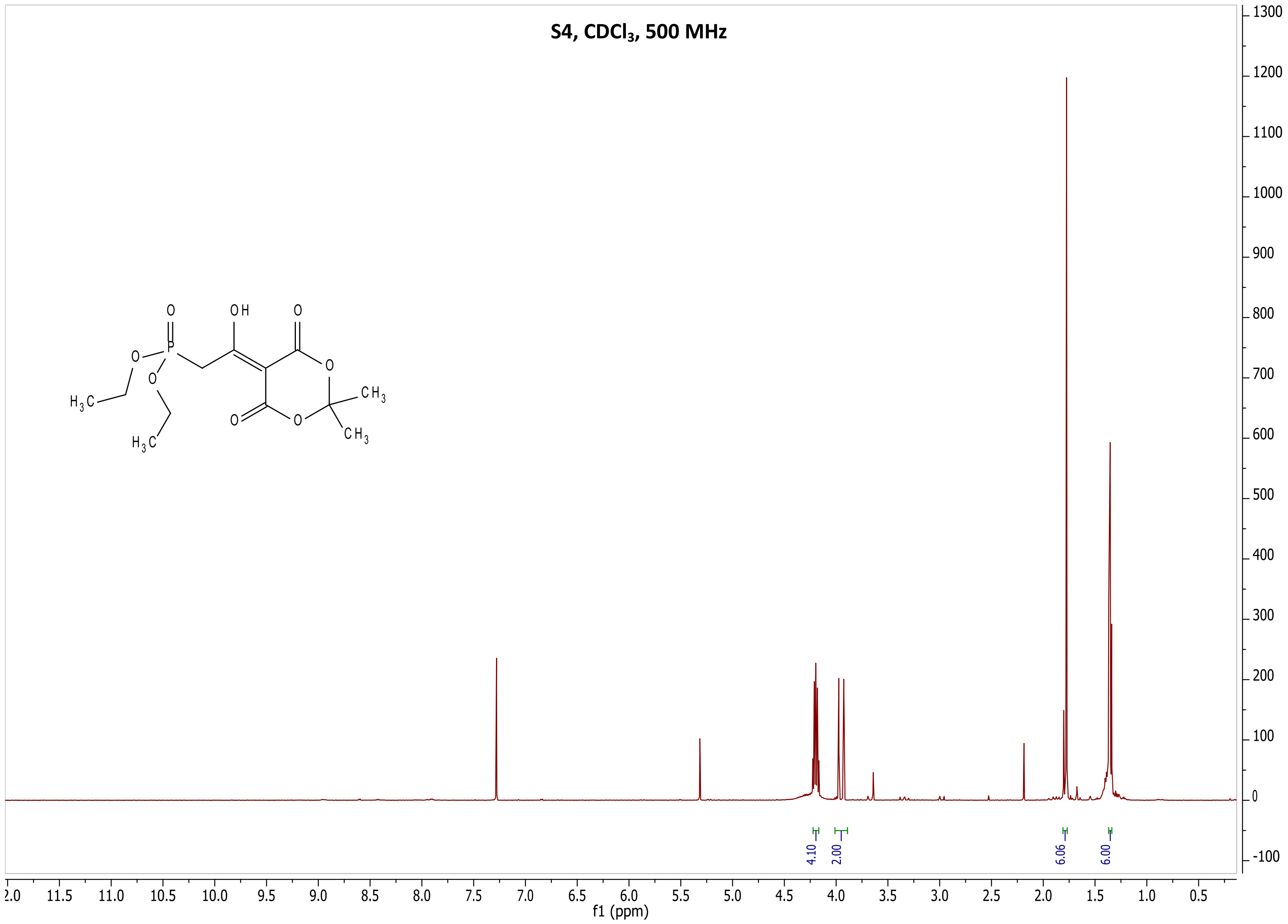
S4, CDCl3, 500 MHz
f1 (ppm)
4.10
2.00
6.06
6.00

S4, $CDCl_3$, 125 MHz

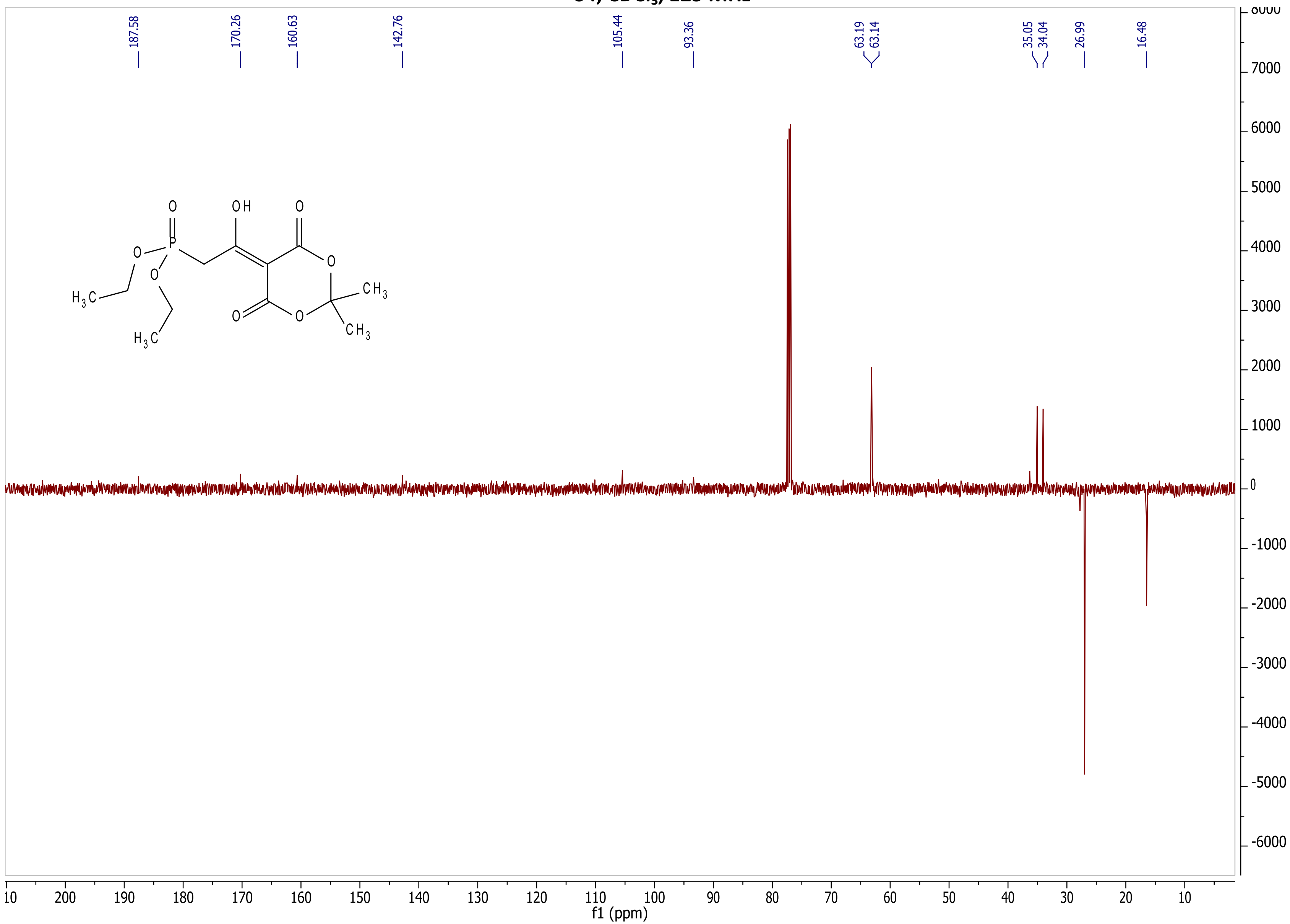

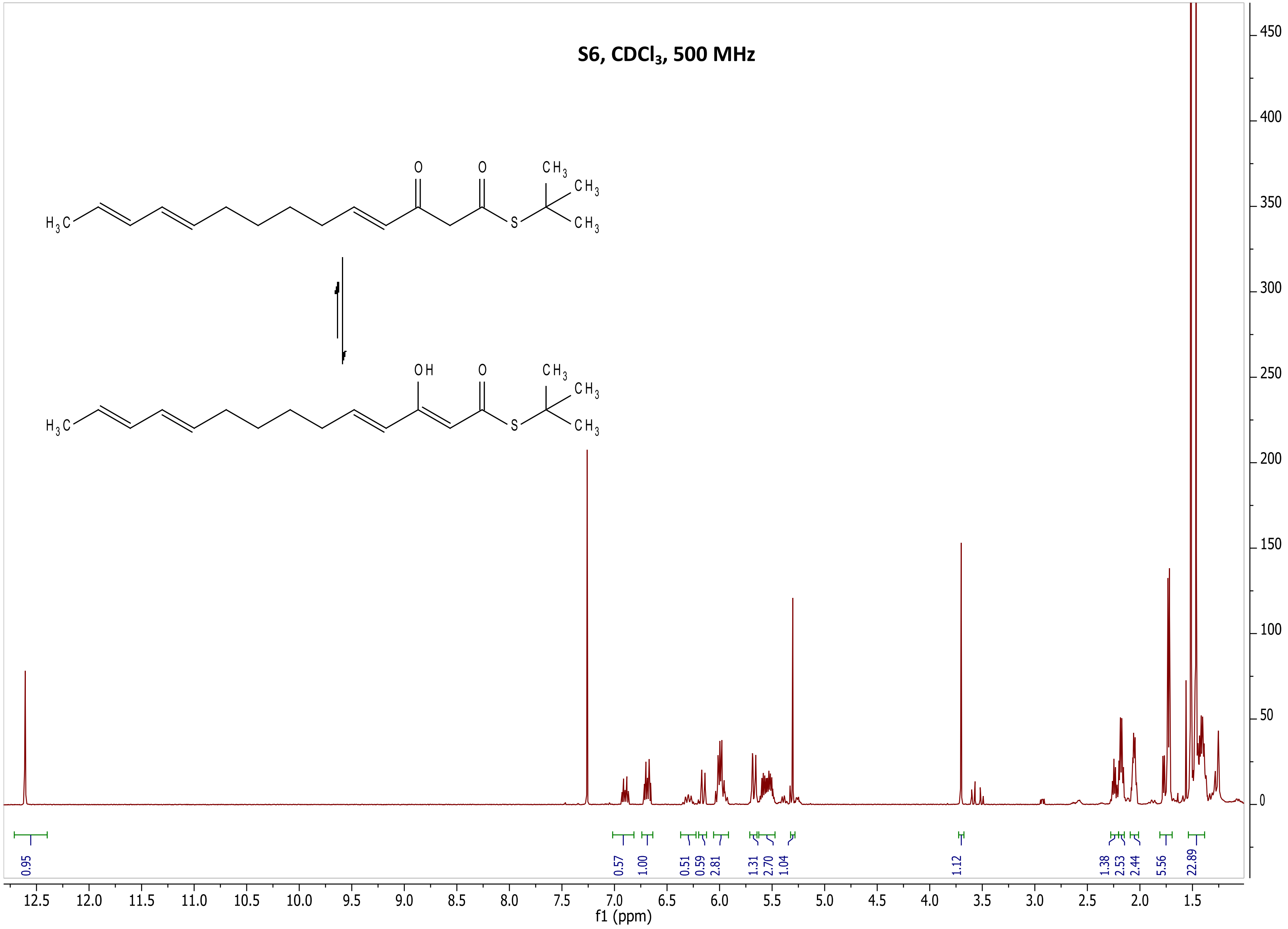
S6, CDCl3, 500 MHz
O
O
CH3
CH3
H3C
S
CH3
OH
O
CH3
CH3
H3C
S
CH3
0.95
0.57
1.00
0.51
0.59
2.81
1.31
2.70
1.04
1.12
1.38
2.53
2.44
5.56
22.89
f1 (ppm)

S6, $CDCl_3$, 125 MHz

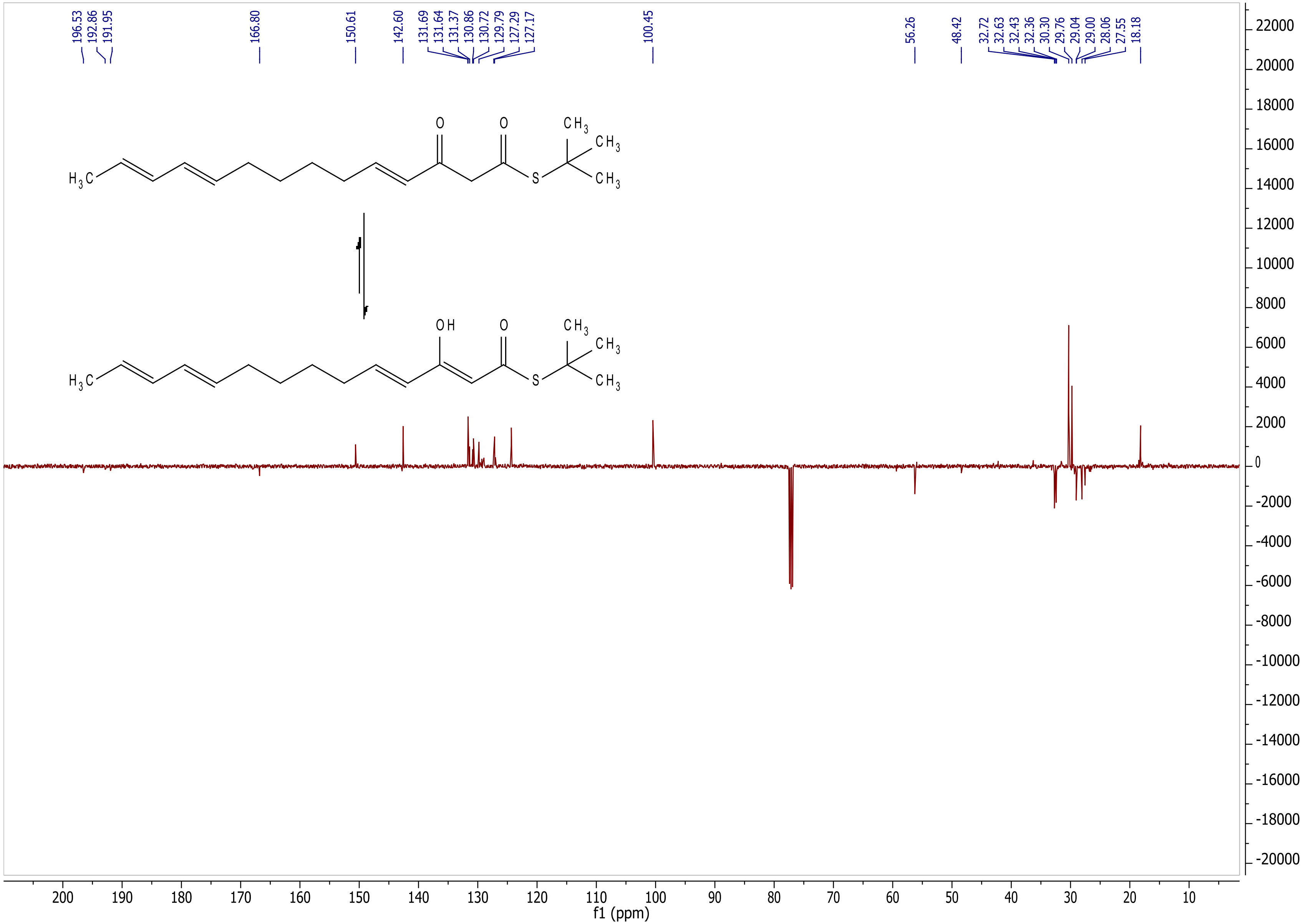

9 Bibliography

[1] T. Kochi, T. Tang, J. Ellman, *J. Am. Chem. Soc.* **2002**, 6518–6519.

[2] T. Kochi, T. P. Tang, J. Ellman, *J. Am. Chem. Soc.* **2003**, *125*, 11276–82.

[3] G. F. Kaufmann, R. Sartorio, S.-H. Lee, C. J. Rogers, M. M. Meijler, J. a Moss, B. Clapham, A. P. Brogan, T. J. Dickerson, K. D. Janda, *Proc. Natl. Acad. Sci. U. S. A.* **2005**, *102*, 309–14.

[4] C. A. Lowery, J. Park, C. Gloeckner, M. M. Meijler, R. S. Mueller, H. I. Boshoff, R. L. Ulrich, Barry Clifton E., D. H. Bartlett, V. V Kravchenko, et al., *J. Am. Chem. Soc.* **2009**, *131*, 14473–14479.

[5] G. F. Kaufmann, R. Sartorio, S.-H. Lee, J. M. Mee, L. J. Altobell, D. P. Kujawa, E. Jeffries, B. Clapham, M. M. Meijler, K. D. Janda, *J. Am. Chem. Soc.* **2006**, *128*, 2802–2803.

[6] R. M. Wilson, W. S. Jen, D. W. C. Macmillan, *J. Am. Chem. Soc.* **2005**, *127*, 11616–7.

[7] D. Könning, W. Hiller, M. Christmann, *Org. Lett.* **2012**, *14*, 5258–5261.

[8] R. M. de Figueiredo, M. Voith, R. Fröhlich, M. Christmann, *Synlett* **2007**, *2007*, 391–394.

[9] S. M. Ma, J. W.-H. Li, J. W. Choi, H. Zhou, K. K. M. Lee, V. a Moorthie, X. Xie, J. T. Kealey, N. a Da Silva, J. C. Vederas, et al., *Science* **2009**, *326*, 589–92.

[10] R. Sawa, Y. Mori, H. Iinuma, *J. Antibiot. (Tokyo).* **1994**, *47*, 731–732.

[11] L. Reddy, A. Gupta, Y. Liu, *J. Org. Chem.* **2011**, 3409–3415.

[12] S. Schreiber, R. Claus, J. Reagan, *Tetrahedron Lett.* **1982**, *23*, 3867–3870.

[13] K. Uchida, T. Ogawa, Y. Yasuda, H. Mimura, T. Fujimoto, T. Fukuyama, T. Wakimoto, T. Asakawa, Y. Hamashima, T. Kan, *Angew. Chem. Int. Ed. Engl.* **2012**, *51*, 12850–3.

[14] B. B. Snider, Q. Lu, *J. Org. Chem.* **1996**, *61*, 2839–2844.

[15] P. M. Booth, H. B. Broughton, M. J. Ford, C. M. J. Fox, S. V. Ley, A. M. Z. Slawin, D. J. Williams, P. R. Woodward, *Tetrahedron* **1989**, *45*, 7565–7580.
